# SBMLsqueezer 2: context-sensitive creation of kinetic equations in biochemical networks

**DOI:** 10.1186/s12918-015-0212-9

**Published:** 2015-10-09

**Authors:** Andreas Dräger, Daniel C Zielinski, Roland Keller, Matthias Rall, Johannes Eichner, Bernhard O Palsson, Andreas Zell

**Affiliations:** Systems Biology Research Group, University of California, San Diego, 9500 Gilman Drive, La Jolla, 92093-0412 CA USA; Center for Bioinformatics Tuebingen (ZBIT), University of Tuebingen, Sand 1, Tübingen, 72076 Germany; Novo Nordisk Foundation Center for Biosustainability, Kogle Allé 6, Hørsholm, 2970 Denmark

**Keywords:** Biological networks, Information extraction, Mathematical modeling, Metabolic engineering, Regulatory networks, Software engineering

## Abstract

**Background:**

The size and complexity of published biochemical network reconstructions are steadily increasing, expanding the potential scale of derived computational models. However, the construction of large biochemical network models is a laborious and error-prone task. Automated methods have simplified the network reconstruction process, but building kinetic models for these systems is still a manually intensive task. Appropriate kinetic equations, based upon reaction rate laws, must be constructed and parameterized for each reaction. The complex test-and-evaluation cycles that can be involved during kinetic model construction would thus benefit from automated methods for rate law assignment.

**Results:**

We present a high-throughput algorithm to automatically suggest and create suitable rate laws based upon reaction type according to several criteria. The criteria for choices made by the algorithm can be influenced in order to assign the desired type of rate law to each reaction. This algorithm is implemented in the software package SBMLsqueezer 2. In addition, this program contains an integrated connection to the kinetics database SABIO-RK to obtain experimentally-derived rate laws when desired.

**Conclusions:**

The described approach fills a heretofore absent niche in workflows for large-scale biochemical kinetic model construction. In several applications the algorithm has already been demonstrated to be useful and scalable. SBMLsqueezer is platform independent and can be used as a stand-alone package, as an integrated plugin, or through a web interface, enabling flexible solutions and use-case scenarios.

**Electronic supplementary material:**

The online version of this article (doi:10.1186/s12918-015-0212-9) contains supplementary material, which is available to authorized users.

## Background

Models of biochemical networks are being constructed on increasingly large scales [1, 2]. Automatic procedures have been suggested to derive draft networks from annotated genomes, such as the Model SEED [3]. These models promise to serve as *in silico* experimentation platforms to probe complex biological systems. The reconstruction of genome-scale networks is a highly laborious long-term effort, which requires intensive curation [4]. However, when building kinetic models, the determination of the underlying network structure is just the first step [5]. For each reaction within the network, a specific kinetic equation, or rate law, needs to be derived. These rate laws typically contain parameters such as Michaelis constants that must be defined [6].

A large number of software suites exist that allow users to specify rate laws for kinetic modeling. Programs, such as COPASI [7], CellDesigner [8], the MASS Toolbox^1^, and Cellerator [9], provide pre-defined lists of kinetic equations and also allow the user to modify these rate laws or to even create customized equations. CellDesigner 4.4 provides a dialog that assists the user to obtain rate laws from the kinetics database System for the Analysis of Biochemical Pathways Reaction Kinetics (SABIO-RK) [8, 10]. The MASS-Toolbox focuses on the creation of elementary rate laws and automatically derives pseudo-elementary rate constants with their units. Inference programs, such as Net*Gene*rator [11, 12], estimate a topology and generate specific rate laws for gene-regulatory processes. Odefy [13] converts discrete Boolean networks into quantitative differential equation systems by applying Hill-type rate laws [14] to each transition.

However, existing software tools are not designed to quickly construct rate laws for large models. Manually deriving both kinetic equations and all units brings several problems with it, because it is *a*) highly error-prone, and *b*) time-consuming, and thus is undesirable in large-scale or automated approaches. For these reasons, automatic procedures are highly desirable for the assembly of rate laws. Furthermore, standard data formats would be useful for encoding of networks and provide a formalization of concepts and data structures that enable cross-platform use of created models [15].

We introduce SBMLsqueezer 2, a software package designed for rapid, consistent prototyping of large-scale biochemical kinetic models. SBMLsqueezer 2 aids the user in the model construction process by applying several criteria to automatically suggest appropriate equations for each reaction. The user can influence these criteria and choose which rate law to apply. The aims of this approach are *a*) to ensure that only applicable rate laws can be selected and thus ensure the consistency of the model, and *b*) to reduce the required manual labor and error checking to a minimum.

SBMLsqueezer 2 is intended to be useful for modeling not only metabolic networks but also signal transduction processes and gene-regulatory mechanisms.

This article describes the details of a method that we call *context-sensitive rate law assignment*. We explain the implementation of this approach in the software SBMLsqueezer 2 and discuss possible use-case scenarios.

## Implementation

### Software architecture

**Fig. 1:**
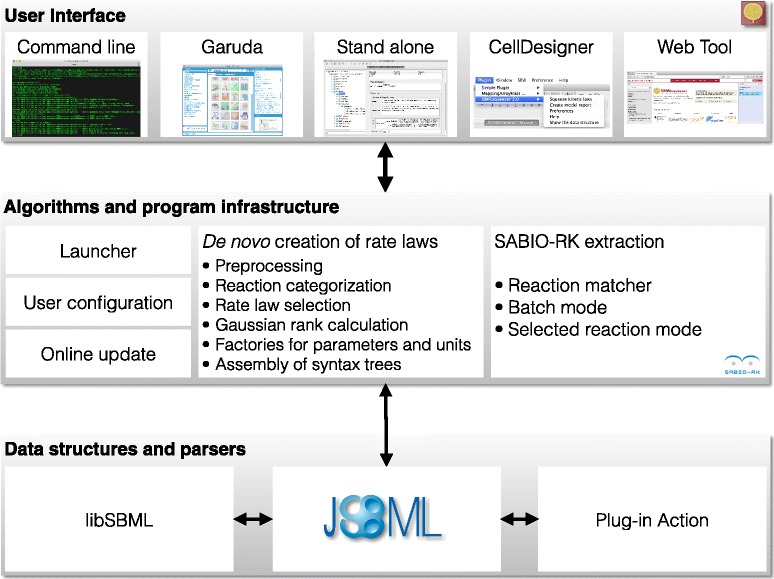
Architecture of SBMLsqueezer 2. An important design principle of the program is to be compatible with various frameworks. To this end, the program is modularized in three layers. The first layer is the user interface. This layer allows users to utilize the program in multiple ways, including a stand alone version with an own GUI that can also be launched from Garuda’s dashboard [24], a fully featured command-line interface, as a plugin of CellDesigner [21], or as an online program in Galaxy [22]. The web version of the program enables users to easily build complex workflows with other programs in the Galaxy framework. Similar pipelines can be achieved when using SBMLsqueezer as a Garuda gadget. No matter how the program is launched, each mode has access to the identical algorithms and program infrastructure in the second layer. The only exception is that the SABIO-RK [10] has been deactivated in the CellDesigner plugin because CellDesigner provides its own module for this purpose [8]. For software developers, this second layer can be accessed directly through its API. Hence, the algorithms can be embedded in more complex processes and be used as a module in third-party programs. The third layer contains the data structures. SBMLsqueezer highly relies on the library JSBML [18] for model representation. When being used as a plugin from CellDesigner or with libSBML [20] as model parsing and writing engine, an additional synchronization step is required: In both cases all data structures that the program receives from either CellDesigner or libSBML are mapped to a corresponding JSBML representation. All changes made by the program must then be reported to the original source

SBMLsqueezer has been planned and implemented as a modular program that follows established software design patterns, such as the Model-View-Controller pattern, and hence strictly discriminates between its (graphical or command-line) user interface, its data model, algorithms, etc. A schematic of the program’s design can be seen in Fig. 1.

SBMLsqueezer contains a core package that provides a general infrastructure for the program. The core deals with user preferences and command-line arguments (see Additional file 1), and searches for online updates. Furthermore, the core is responsible to launch the program. This can be done in diverse ways, e.g., in command line mode, as a plugin of CellDesigner, as a gadget in Garuda, etc.

A graphical user interface can be launched from the core and has then control over all functions of the program. For details about which functions are available and how to use the user interface, see Additional file 1.

All implemented rate laws are gathered in the kinetics package and are grouped by twelve interfaces that are described in the next section. For version 2, a new SABIO-RK package has been implemented that obtains kinetic equations from the rate law database SABIO-RK [10]. A mathematics package contains an implementation of the Gaussian rank calculation, which is required for convenience rate laws [16].

### Data structures and dependencies

SBMLsqueezer 2 is based on the data format Systems Biology Markup Language (SBML) [17]. The internal data structure of the program is provided by JSBML [18, 19]. Converters can read and write input SBML documents through libSBML [20] or CellDesigner’s Application Programming Interface (API) [21], or JSBML can directly parse these files. In this way, JSBML acts as an abstraction layer between diverse forms of input and synchronizes all changes made by the program back to the original source. In case that JSBML is being directly used, the synchronization step can be omitted. It is entirely implemented in Java™ and runs on every platform for which a Java™ is available. Reading and writing of SBML files is done with JSBML [18], which also acts as the internal data structure. SBMLsqueezer 2 can also be launched using a libSBML [20] back-end. The online program version is based on the command-line interface of the stand-alone tool, which is wrapped in a Galaxy [22] framework. For writing model reports, SBMLsqueezer 2 contains a development release of SBMLLaTeX [23]. The Garuda gadget [24] is implemented based on the back-end API for Java™. The CellDesigner plugin uses the communication interface between CellDesigner’s plugin API and JSBML. Changes made by SBMLsqueezer 2 are synchronized with CellDesigner through a change listener interface. SBMLsqueezer 2 determines the type of reaction by interpreting Systems Biology Ontology (SBO) and Minimal Information Required In the Annotation of Models (MIRIAM) annotations [25] of all components as well as the number and kind of reaction participants. Access to SABIO-RK [10] requires an active Internet connection and uses the Representational State Transfer (RESTful) API provided by SABIO-RK through a Java™ Uniform Resource Locator (URL) connection.

It should be noted that for some levels and versions of SBML numbers cannot be associated with units and that some rate laws can under certain conditions not be evaluated to reaction extend per time units.

When being used as a CellDesigner plugin, the following special cases apply: *a* SBO terms are inferred from the CellDesigner-specific annotations of modifiers and further elements. *b* a special observer class synchronizes all changes from the submodel to the data model of CellDesigner.

### Preprocessing

The *de novo* rate law selection algorithm performs several preprocessing steps, iterating through all reactions within the submodel *M*^′^ (see Algorithm 3 in Additional file 2:

1. If the user defines a list of Kyoto Encyclopedia of Genes and Genomes (KEGG) [26] Identifiers (IDs) for species whose contribution to rate laws should be neglected, species with such terms in their MIRIAM annotation [27] are removed. Table 1 shows the predefined list of those entities.

**Table 1:** KEGG compound ID of small molecules and ions. This table gives the default list of all small molecules and ions that SBMLsqueezer ignores when creating kinetic equations. This list was created according to [68] and can be changed by the user through command-line options as well as a preferences dialog

Chemical formula	Common name	KEGG ID
H_2_O	Water	C00001
Zn^2+^	Zinc ion	C00038
Cu^2+^	Copper ion	C00070
Ca^2+^	Calcium ion	C00076
H^+^	Proton	C00080
Co^2+^	Cobalt ion	C00175
K^+^	Potassium ion	C00238
H_2_	Hydrogen	C00282
Ni^2+^	Nickel ion	C00291
Cl^−^	Chloride ion	C00698
HCl	Hydrochloric acid	C01327
H_2_Se	Hydrogen selenide	C01528
Fe^2+^	Iron (II) ion	C14818
Fe^3+^	Iron (III) ion	C14819
2. The stoichiometry of each reaction participant is analyzed in order to obtain the accumulated stoichiometry of reactants and products and to check if all values are integers. In this step the algorithm also analyzes the SBO term [25] attribute of each reaction participant. Further top-level SBO terms with relevance to the algorithm can be found in Table 2. The aim of this step is to get hints if the reaction represents a transcription or translation:
  If a reactant represents a gene or gene-coding region, the reaction could be a transcription.

**Table 2:** SBO terms with relevance for the categorization of reactions. The preprocessing algorithm (see Algorithm 3 in Additional file 2) uses the SBO terms listed in this table in order to distinguish between different types of species and modification in order to categorize a each reaction in the submodel as well as the role of individual reaction participants. This also includes further relevant material entities of reaction participants. Note that the algorithm always checks if the SBO term of an element is a child of a certain reference term in order to also include all more specific sub-terms

Definition	SBO term
Catalyst	SBO:0000013
Empty set	SBO:0000291
Enzymatic catalyst	SBO:0000460
Gene	SBO:0000243
Gene-coding region	SBO:0000335
Generic	SBO:0000252
Inhibition	SBO:0000169
Inhibitor	SBO:0000020
MRNA	SBO:0000278
Necessary stimulation	SBO:0000171
Protein	SBO:0000297
RNA	SBO:0000250
Stimulation	SBO:0000170
Stimulator	SBO:0000021
Transcriptional activation	SBO:0000459
Transcriptional inhibition	SBO:0000020
Translational activation	SBO:0000459
Trigger (necessary stimulator)	SBO:0000461
Translation	SBO:0000184
Transcription	SBO:0000183
  If the reaction involves a reactant that stands for a Ribonucleic Acid (RNA) or Messenger RNA (mRNA) molecule it could be a translation.
  If at least one modifier represents a gene or RNA molecule, the reaction could represent a translation.
  Figure 2. A depicts these processes in a simple schematic based on the Systems Biology Graphical Notation (SBGN) recommendations [28]. The algorithm recognizes these these reaction patterns only based on the stoichiometry and SBO term annotation of all participants.

**Fig. 2:**
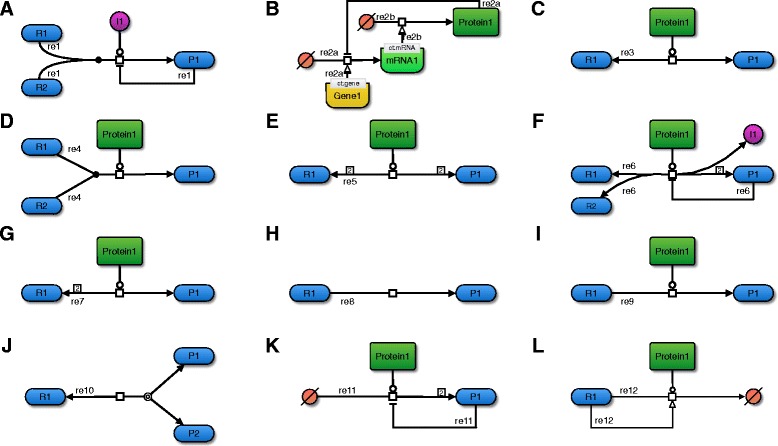
Examples for general reaction categories. This figure displays example reactions in SBGN for each of the twelve categories that are used to determine applicable rate equations. **a**) Reaction with a non-enzyme catalyst. The ion I1 catalyzes this association reaction, which can therefore not be considered enzyme catalyzed. In addition, this reaction is also modulated in a feedback inhibition loop, has an integer stoichiometry, and two reactants. **b**) Gene-regulatory processes. Reaction re2a assembles an mRNA molecule from a source of bases (transcription), enabled by the a specific gene. This mRNA in turn (re2b) enables the assembly of a protein from a source of amino acids (translation). In a feedback inhibition loop, the protein interferes with the transcription of its own gene. **c**) Uni-uni enzyme reaction. This schematic conforms the classical Michaelis-Menten mechanism. **d**) Bi-uni enzyme reaction. This association reaction has an integer stoichiometry. **e**) Bi-bi enzyme reaction. In this example, two molecules of identical type act as reactants and also as products, respectively. **f**) Arbitrary enzyme reaction. This reversible reaction involves a feedback inhibition and a complex stoichiometry, in which an ion and two identical molecules are created from two distinct reactants. **g**) Integer stoichiometry. This reversible, enzyme-catalyzed reaction has two identical reactants and one product. **h**) Irreversible reaction. This reaction has no explicit catalyst assigned to it. Depending on user-settings, the algorithm can still consider this an enzyme-catalyzed reaction, assuming that the omission of the catalyst is for the sake of simplicity. **i**) Modulated reaction. Both, a stimulator and an inhibitor interfere with this reaction. **j**) Reversible reaction. This dissociation reaction can also be seen as an association when the equilibrium shifts to the reverse reaction. **k**) Zeroth reactant order reaction. The two product molecules lower the velocity of their own creation. **l**) Zeroth product order reaction. The reactant stimulates its own degradation
3. Based on their SBO term [25] attribute all modifiers of the reaction are grouped into the following sets: *a*) enzymes; *b*) activators; *c*) inhibitors; and *d*) non-enzyme catalysts.
  Since it is not always clear if a catalyst of a reaction is an enzymatic catalyst, the user can define which kinds of species may be considered enzymes in the specific context. 3 presents a list of all kinds of species that the algorithm can potentially accept as enzymes of a reaction. The algorithm checks if any modifier of the reaction corresponds to a species with one of the SBO terms in this list. If the modifier is annotated as *catalyst* (SBO:0000013) and its corresponding species belongs to the list of potential enzymes, the algorithm assigns the SBO term *enzymatic catalyst* (SBO:0000460) to the modifier. Based on the user’s selection the algorithm hence solves contradictions between the SBO term of a modifier and the corresponding species. See Table 3.

**Table 3:** Kinds of species that can potentially act as enzymes and their top-level SBO terms. In the preprocessing step (see Algorithm 3 in Additional file 2), the algorithm for the *de novo* creation of kinetic equations categorizes reaction modifiers into four different groups: enzymes *E*, activators and stimulators *A*, inhibitors *I*, and non-enzyme catalysts *C*. To this end, it first analyzes the SBO term assigned to the modifier itself (see Table 2). It then obtains the actual species that acts as a modifier in the current reaction and analyzes this species’ SBO terms, assuming that this annotation defines the material classes of the species. The SBO terms in this table represent the top-level terms, i.e., the algorithm also accepts each more specialized term for a specific category. Note that the user can exclude elements from this list and therefore influence the algorithm’s choices. If the material class of a modifier’s species falls into one of the allowable SBO categories, the algorithm will categorize this modifier as an enzyme and potential contradictions with the modifier’s SBO term will be resolved

Material entity	SBO term
Antisense RNA (asRNA)	SBO:0000317
Complex	SBO:0000253
Generic protein	SBO:0000252
Macromolecule	SBO:0000245
Receptor	SBO:0000244
RNA	SBO:0000250
Simple molecule	SBO:0000247
Truncated protein	SBO:0000248
Unknown molecule	SBO:0000285
4. In order to ensure that the algorithm can process each reaction within submodel *M*^′^, it checks the following semantic rules:
  If a reaction involves a gene or gene-coding region as reactant or its set of reactants is *empty* and all products are RNA molecules, the reaction is recognized as transcription.
  If the substrate species of a reaction are RNA molecules or the reaction does not have any reactants and all products are forms of protein or poly-peptide chains, the reaction is recognized as translation.
  If the stoichiometry of reactants and products is unity, the reaction can only be categorized as transcription if the only reactant is a gene or gene-coding region and it is only a valid translation if the only reactant is an RNA molecule.

More formally, a reaction is recognized as a transcription if

(1) $$ \begin{aligned} \left(r_{\text{allGenes}} \wedge (r_{\text{Stoichiometry}} = 1) \wedge (p_{\text{Stoichiometry}} = 1)\right) \\[-3pt] \vee \left((R_{r} = \varnothing) \wedge p_{\text{allRNA}}\right) \end{aligned}  $$

and the reaction will be considered a translation if

(2) $$ \begin{aligned} \left(r_{\text{allRNA}} \wedge (r_{\text{Stoichiometry}} = 1) \wedge (p_{\text{Stoichiometry}} = 1)\right) \\[-3pt] \vee \left((R_{r} = \varnothing) \wedge p_{\text{allPolypeptides}}\right). \end{aligned}  $$

Here, the Boolean variables *r*_allGenes_ and *r*_allRNA_ are true if all reactants represent genes or RNA, respectively, and the values *r*_Stoichiometry_ and *p*_Stoichiometry_ denote the accumulated stoichiometry of all reactants or products, respectively, in sets of reactants *R*_*r*_ and products *P*_*r*_ of reaction *r*. The Boolean variables *p*_allRNA_ and *p*_allPolypeptides_ are true if the reaction produces RNA or polypeptide molecules, respectively. A set of reactants or products is said to be *empty* if either *a*) no such set exists in reaction *r*; *b*) no element has been assigned to this set; *c*) the stoichiometry of each element within the list is zero; or *d*) if each element in the list is annotated with an SBO term derived from the term for *empty set* (SBO:0000291).

Depending on user preferences, the algorithm can set the boundary condition for each gene as part of this step.

### Rate law selection

After having reaction preprocessing and semantic checking completed, the algorithm assigns a list of applicable categories to each reaction within the submodel *M*^′^. Algorithm 4 in Additional file 2 depicts this procedure in detail. Thereby the algorithm distinguishes between twelve such categories that are defined in Table 4. Figure 2 displays examples for each category. These categories are not necessarily exclusive.

**Table 4:** Reaction categories. This table lists and describes reaction categories with relevance to automatic rate law assignment. Graphical examples for each category can be seen in Fig. 2. These categories are not mutually exclusive. A reaction can therefore belong to multiple categories. Similarly, all available rate laws are also assigned to these categories (see Table 5). Since also each rate law can belong to multiple categories, some of these categories are used to refine the selection of rate laws for a reaction, i.e., some categories are exclusive. A rate law may belong to the category of reversible (J) and irreversible (H) reactions if it is possible to apply the rate law to both types of reactions, but a rate law from category G can only be applied to a reaction in which all participants have a strictly integer stoichiometry. Rate laws that have mechanisms for modulation can also be applied to non-modulated reactions, but rate laws that do not belong to category I cannot be applied if activators or inhibitors interfere with the reaction

${\text {N}}^{\underline {o}}$	Category	Description
A	Non-enzyme reactions	Spontaneous reactions and reactions with a catalyst that is no enzyme.
B	Gene-regulatory processes	Reactions that produce RNA or produce polypeptide molecules from an empty set of reactants or whose reactants are genes or RNA molecules and that have genes or RNA molecules as modifiers.
C	Uni-uni enzyme reactions	Enzyme-catalyzed reactions with one reactant of stoichiometry one and if reversible also one product of stoichiometry one.
D	Bi-uni enzyme reactions	Enzyme-catalyzed reactions with two reactants, i.e., an integer stoichiometry two on the reactant side, and if reversible one product of stoichiometry one.
E	Bi-bi enzyme reactions	Enzyme-catalyzed reactions with two reactants, i.e., an integer stoichiometry two on the reactant side, and if reversible two products that also have a stoichiometry of two.
F	Arbitrary enzyme reactions	Enzyme-catalyzed reactions with an arbitrary number of reactants and products.
G	Integer stoichiometry reactions	Reactions whose participants have only integer stoichiometric values.
H	Irreversible reactions	Reactions whose net flux proceeds only in forward direction.
I	Modulated reactions	Reactions whose velocity is influenced by modifiers, such as activators (stimulators), inhibitors, an (enzymatic) catalysts
J	Reversible reactions	Reactions that can proceed in forward and reverse direction.
K	Zeroth reactant order reactions	Reactions in which the effects of reactants do not contribute to the velocity.
L	Zeroth product order reactions	The effects of products do not influence the velocity of these reactions.

**Table 5:** Rate laws and their reaction categories. This table gives an overview of all rate laws that are currently implemented in the software SBMLsqueeezer 2. All rate laws are described in detail in [67, p. 17–38]. The key idea of the algorithm in this paper is to separate the list of available rate laws from more general reaction categories to which these rate laws can be applied. With this separation, extending this list and to add more specific rate laws becomes very straightforward to do as long as each such rate law specifies to which categories it can be assigned. Table 4 provides a detailed overview of all categories. Example reactions for all categories can be seen in Fig. 2. The rate laws mentioned in this table are again families of equations. The precise structure of the equation that is generated for a specific reaction can vary, depending on several reaction properties, e.g., how many and which kind of modifiers participate in this reaction, if it is reversible, etc. The creation of the specific equation including its parameters and required units is therefore the next step of the algorithm

Rate laws	Categories	Citation
Additive Model Linear	B, H, I, J, K, L	[45]
Additive Model Non Linear	B, H, I, J, K, L	[46]
Common Modular Rate Law (CM)	C, D, E, F, I, J	[35]
Convenience Kinetics	C, D, E, F, H, I, J	[16]
Direct Binding Modular Rate Law (DM)	C, D, E, F, I, J	[35]
Enzymatic Rate Law for Competitive Inhibition of Irreversible Uni-reactant Enzymes by Non-Exclusive Non-Cooperative Inhibitors	C, H, I	SBO
Enzymatic Rate Law for Irreversible Non-modulated Non-interacting Reactant Enzymes	C, D, E, F, G, H	SBO
Force Dependent Modular Rate Law (FM)	C, D, E, F, I, J	[35]
Generalized Mass Action	A, H, I, J	[29], [30, p. 14–17]
Hill Equation	B, C, G, H, I, J	[14], [70, p. 314]
Hill-Hinze Equation	B, H, I, J, K, L	[42]
Hill-Radde Equation	B, H, I, J, K, L	[43, 44]
H-System	B, H, I, J, K, L	[51]
Michaelis-Menten	C, H, I, J	[71]
Net*Gene*rator Linear	B, H, I, J, K, L	[46]
Net*Gene*rator Non-Linear	B, H, I, J, K, L	[46]
Ordered Mechanism (compulsory-order ternary-complex mechanism)	D, E, H, I, J	[72, 73], [70, p. 167]
Ping-Pong Mechanism (substituted enzyme mechanism)	E, H, I, J	[72, 73], [70, p. 169]
Power Law Modular Rate Law (PM)	C, D, E, F, I, J	[35]
Random Order Ternary-Complex Mechanism	D, E, H, I, J	[72, 73], [70, p. 169]
Simultaneous Binding Modular Rate Law (SM)	C, D, E, F, I, J	[35]
S-System	B, H, I, J, K, L	[50, 51, 74–76]
Vohradský’s equation	B, H, I, J, K, L	[47, 48]
Weaver’s equation	B, H, I, J, K, L	[49]
Zeroth Order Forward Generalized Mass-Action	A, H, I, J, K, L	[70, p. 6]
Zeroth Order Reverse Generalized Mass-Action	A, H, I, J, K, L	[70, p. 6]

All kinetic equations are also assigned to one or multiple of these categories. The algorithm may now either collect all appropriate rate laws for the obtained reaction categories (types) or just the one rate law with highest priority. While the first method allows users to interactively select rate laws of choice, the latter option is important for the automatic selection of the most appropriate equation. See Table 2. The selection of one or multiple appropriate categories and in turn suitable kinetic equations for a reaction is based on a set of defined rules, which are here summarized and simplified for the sake of better comprehensiveness. The algorithm distinguishes the following three basic cases, which are not necessarily exclusive:

1. The list of reactants is *empty* or the reaction is reversible and the list of products is *empty*. If the reaction does neither involve genes, gene-coding regions, nor RNA molecules, then the algorithm can assign it to the zeroth reactant order reactions if also the list of reactants is *empty*, and to the zeroth product order reactions if it is reversible with an empty list of products. If it does involve genetic components, it can be assigned to the gene-regulatory reactions depending on its directionality. More formally, this category is selected if
  (3) $$ \begin{aligned} \neg \left(r_{\text{allGenes}} \wedge r_{\text{allRNA}} \wedge p_{\text{allRNA}} \right) (\wedge R_{r} = \varnothing)\\ \vee \left(\text{isReversible}(r) \wedge (P_{r} = \varnothing)\right) \end{aligned}  $$
  and the category for zeroth reactant order rate laws will be assigned if $R_{r} = \varnothing $, otherwise with $P_{r} = \varnothing $ the category for zeroth product order rate laws will be assigned.
2. The reaction has at least one reactant and if it is reversible also at least one product. If the reaction does neither have any enzymatic catalyst nor any non-enzymatic catalyst, it is assigned to the category of non-enzyme reactions with respect to its directionality. In case of unity stoichiometry on both sides of the reaction, and if the reaction follows the pattern of transcription or translation reactions, it is added to the category of gene-regulatory reactions. The pattern is satisfied if the reactant is a genetic element or an empty set and the product is an RNA molecule or protein. More formally, the algorithm first evaluates the condition
  (4) $$ \begin{aligned} &(r_{\text{Stoichiometry}} \geq 1) \wedge \left(\neg \text{isReversible}(r)\right.\\ &\vee \left.\left(\text{isReversible}(r) \wedge (p_{\text{Stoichiometry}} \geq 1)\right)\right)\,. \end{aligned}  $$
  If then ($E = \varnothing) \wedge (C = \varnothing) \wedge \neg e$ the reaction can be assigned to the non-enzyme reaction, where *e* is the user preference that decides if reactions without explicit enzyme can be considered enzyme catalyzed. Otherwise, if *r*_Stoichiometry_=1∧*p*_Stoichiometry_=1∧(*r*_allGenes_∨*r*_allRNA_) the reaction will be considered a gene-regulatory reaction.
3. The preprocessing has revealed that the reaction is catalyzed by an enzyme. Depending on user preferences, a reaction can also be recognized as enzyme-catalyzed process if no catalytic modifier is assigned to it. If the reaction is reversible with at least one product, the category of arbitrary enzyme reactions is assigned. Next, the stoichiometry and directionality of the reaction are taken into account in order to determine if the reaction also belongs to the uni-uni, bi-uni, or bi-bi reactions. Formally, this condition can be described as
  (5) $$ \left((E \neq \varnothing) \vee e\right) \vee \text{isReversible}(r) \wedge p_{\text{Stoichiometry}} \geq 1.  $$

Each reaction category is represented with one interface that can be implemented by rate laws that are applicable for this category. Since one rate law can be useful for multiple categories, rate laws can also implement several of these interfaces. For all categories that can be applied to a reaction, the algorithm then compiles a list of concrete kinetic equations.

The list of applicable kinetic equations is hence generated on the fly and only based on the general reaction categories. In this way, the program can easily be extended, because additional kinetic equations only need to declare the categories to which they can be applied and will automatically be available when the program is executed.

Additional rules apply when the algorithm compiles the list of rate laws based on reaction categories, because some rate laws can only be applied to certain combinations of categories. For instance, the *enzymatic rate law for irreversible non-modulated non-interacting uni-reactant enzymes* (SBO:0000150) can only be applied to irreversible reactions with integer but arbitrary stoichiometry, but does neither allow any stimulators nor inhibitors. Thus, some categories exclude certain rate laws from being assigned to a reaction.

The user can select one default rate law for almost all categories. No specific default rate law can be selected for the categories irreversible or reversible reactions, modulated reactions, or integer stoichiometry, because these cases mainly refine the other categories. Instead, the selection of default rate laws is split into three major groups: *a*) gene-regulatory reactions (including reactions with zeroth order reactants or products); *b*) reversible reactions; and *c*) irreversible reactions.

This is necessary because some rate laws can only be applied to reversible reactions, others only to irreversible reactions.

### Prioritization

The identification of the category with highest priority works very similarly. For reactions with empty list of reactants a gene-regulatory reaction has higher priority than the zeroth reactant order (but requires that the reaction follows the right pattern). Similarly, the algorithm first tries to assign a reaction to the gene-regulation category if it is reversible with an empty list of products, before assigning it to the zeroth product order reactions. Non-enzymatic reactions have higher priority than any enzymatic reaction. If the reaction is enzyme-catalyzed, the algorithm tries to first assign the most detailed category before it chooses an arbitrary enzyme reaction category. Hence, the algorithm determines one category for each reaction and applies the default rate law from this category to the reaction. In case of conflicts there are two final fall-back rate laws that can be applied if no other rate law can be selected: *a* for non-enzyme reactions, the generalized mass-action rate law [29, 30]; and *b* the convenience rate law [16] for any kind of enzyme-catalyzed reaction.

Both rate laws can be applied to reversible and irreversible reactions with arbitrary stoichiometry and can be combined with pre-factors for modification (activation or inhibition) as needed. When creating rate laws for individual reactions, a complete list of all applicable rate laws for the reaction of interest is compiled.

When convenience rate laws are used, the algorithm prefers the simple form and applies the thermodynamically independent form only if the system does not have full column rank. To this end, the program calculates the rank of the stoichiometric matrix using the Gaussian algorithm. This rank check is performed only once for the given model and only executed if at least one reaction exists, for which a convenience rate law is selected. Note that the algorithm calculates the rank for the full stoichiometric matrix and not just for the current submodel. This is crucial, because in many cases submodels would only contain one reaction, and thus the rank would be full.

### Rate law creation

**Fig. 3:**
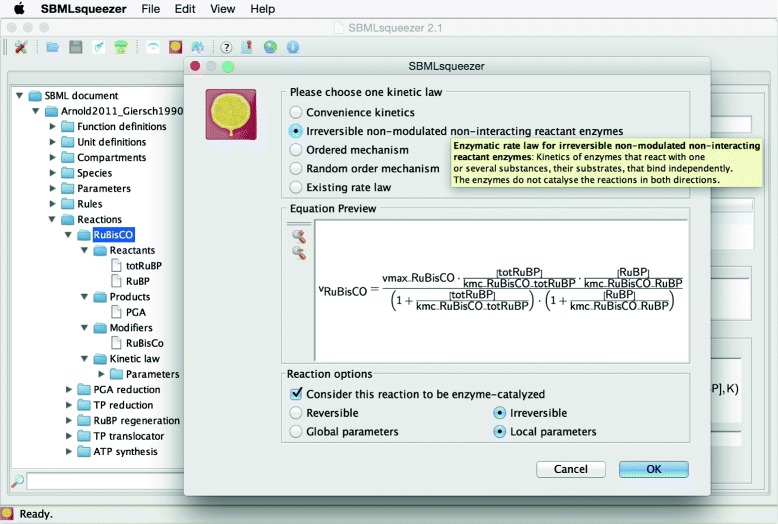
Reaction context dialog of SBMLsqueezer 2. When using SBMLsqueezer as a stand-alone program, this dialog pops up upon right-clicking on a reaction in the model data structure. All available kinetic equations that can be potentially applied to the selected reaction are listed and can be selected via radio buttons. A tool-tip displays detailed information about each rate law and an equation renderer displays a preview of the equation. Furthermore, this dialog also provides a few particularly important settings: a) it allows users to choose whether newly generated parameters should be created as local parameters within the kinetic law or global model parameters, b) if the reaction’s directionality should be changed, and c) if the reaction should be considered an enzyme-catalyzed reaction. In situations where a catalyst is assigned to the reaction that is recognized as an enzyme (see Table 3) or as a non-enzyme this option will not be accessible. The model used in this example is described in [77]

Applying a rate law to the reaction means that the algorithm has to construct an abstract syntax tree, which symbolically represents the kinetic equation for the reaction. To this end, the reaction needs to be analyzed again and all of its components need to be taken into account as relevant for the selected rate law (irreversible equations, for instance, tend to ignore effects of products). An example for such a syntax tree can be seen in Fig. 3, which has been created for the Phosphoglucomutase (PGM) reaction

(6) $$ \mathrm{D}\hbox{-} \mathrm{glucose}\ 1\hbox{-} \mathrm{phosphate}\overset{\mathrm{phosphoglucomutase}}{\rightleftharpoons}\mathrm{D}\hbox{-} \mathrm{glucose}\ 6\hbox{-} \mathrm{phosphate}\kern0.3em , $$

whose schematic corresponds to the diagram displayed in Fig. 2.C. This tree represents a rate law for non-modulated enzymes in reactions with only one substrate molecule, which reads

(7) $$ v_{\text{PGM}}\left(\vec{x}(t), t, \mathbf{S}, \mathbf{W}, \vec{p}\right) = \frac{\frac{v_{\mathrm{m}+}}{K_{\mathrm{M}1}} \cdot \left[\mathrm{g1p}\right] - \frac{v_{\mathrm{m}-}}{K_{\mathrm{M}2}} \cdot \left[\mathrm{g6p}\right]}{1 + \left(\frac{[g1p]}{K_{\mathrm{M}1}} + \frac{[\mathrm{g6p}]}{K_{\mathrm{M}2}}\right)}.  $$

In order to ensure unit consistency of the equation, it can be necessary to multiply or divide reactive species with/by their surrounding compartment. User preferences and the units of the species determine if and which of those operations is required, because in SBML, each reaction should yield units of extent per time. Two cases need to be distinguished:

1. If a species has only substance units and the user decides to bring all species to concentration units, the species must be divided by its surrounding compartment.
2. If a species is given in concentration units and the user wants to bring all species to substance units, then a multiplication of the species with its surrounding compartment is required.

Depending on the type of rate law that is being created and the structural composition of the reaction, a certain number of parameters needs to be constructed. This can include forward or backward rate constants, limiting velocity rates, inhibition or stimulation constants, thermodynamic properties, and many more. These parameters can be incorporated as local or global parameters. To this end, the algorithm first collects all parameters in a separate list and transfers them to the submodel only when a rate law is to be applied. Just as for species, parameter objects need to be equipped with appropriate units. The units of many parameters also depend on the structure of the reaction, for instance, the number and units of all reactants. Because of this connection, it can be necessary to also take the units of compartments into account when deriving the units of parameters in order to obtain extend by time units for the overall rate law. The algorithm equips each newly created parameter with a meaningful name and identifier as well as an appropriate SBO term.

If during this step additional units or unit definitions need to be added to the submodel, these are simplified as much as possible, annotated with a MIRIAM identifier pointing to the Units Ontology, and equipped with meaningful names and identifiers as appropriate. The algorithm avoids creating duplicate unit definitions by checking the model for identical existing unit definitions before adding a new one. If possible, the newly created kinetic equation is also annotated with a corresponding SBO term. Due to the large number of SBO terms that can be created by the algorithm, a comprehensive list of all cases is omitted in this document.

Whenever a kinetic equation that involves stoichiometry values is created for models in SBML Level 3 [31], the algorithm inserts the ID of the corresponding species reference rather than the actual numerical value of the stoichiometry into the rate law. This gives the advantage that model changes can be directly reflected in the rate law, hence increasing the consistency of models. At the same time, it avoids the problem that units and meaning of single numerical values might not always be clear.

### Model merging

Finally, all relevant changes in the submodel are synchronized to the original model. This includes all newly created units and unit definitions, local and global parameters, annotations, mathematical equations, reversibility flags, and boundary condition flags. If species have been deleted from reactions, because their MIRIAM annotation was on the ignore list, this change is skipped and not synchronized, so that the structure of the model will remain identical. In the graphical user interface, the user can also disregard the changes.

### Details of extraction of rate laws from SABIO-RK

The algorithm first generates a URL that is used to query the SABIO-RK database. This URL comprises the search terms and the respective KEGG reaction ID. The URL begins with the prefix http://sabio.h-its.org/sabioRestWebServices/searchKineticLaws/sbml?q=. Each search term and its given value extend this base URL with keyword:value. If a search term is associated with a range (e.g., the temperature), the URL is extended with keyword:[min␣TO␣max]. The keywords for the terms are presented here: http://sabio.h-its.org/layouts/content/docuRESTfulWeb/SearchKeyVoc.gsp. The operator ␣AND␣ connects multiple keyword-value pairs in the query.

The URL for querying points to an Extended Markup Language (XML) document for download. In the case of success, the XML document will be an SBML document with all kinetic laws found for the query. Otherwise, SABIO-RK returns an XML document with an error message and the algorithm terminates with a user message.

Since this algorithm mainly operates on models obtained from SABIO-RK it is not necessary to create a submodel copy of the local model beforehand (as this is done for the *de novo* creation of rate laws). Changes are only applied upon user agreement or in batch mode. This is done by merging required components from the downloaded model into the local model. For this reason, the local model does not change, before rate laws are applied.

## Results and discussion

In this section we will describe: *a* the rate law selection algorithm implemented by SBMLsqueezer 2, *b* the process implemented for extraction of rate laws from SABIO-RK [10], and *c* the new features of the SBMLsqueezer 2 stand-alone software over the previously published SBMLsqueezer plugin for CellDesigner.

The approaches described in this article are based on the model definition in SBML format [32], but similar approaches would also be possible for other modeling formats that support kinetic equations.

Both the rate law construction and extraction algorithms assume that the structure of the systems biology model *M* is known and encoded in the two matrices **S** and **W**, whose interplay can be described by the following equation [33, 34]:

(8) $$ \frac{\mathrm{d}\vec{x}}{\mathrm{d} t} = \mathbf{S}\cdot \vec{v}\left(\vec{x}(t), t, \mathbf{S}, \mathbf{W}, \vec{p}\right),  $$

where *t* denotes the time, $\vec {x}$ the reactive species, **S** the stoichiometric matrix, and $\vec {v}$ the vector of kinetic equations that are to be generated. The modulation matrix **W** [16, 35] and the parameters $\vec {p}$ influence the mathematical structure of the equations in $\vec {v}$. The algorithm also assumes that the user has defined a set *R* of reactions for which rate laws are to be created or extracted from SABIO-RK. *R* may comprise all reactions in *M*, or a selection of particularly interesting reactions. An overview of these two methods to build kinetic equations for *M* can be seen in Algorithm 1 in Additional file 2 for the *de novo* construction and in Algorithm 2 in Additional file 2 for the SABIO-RK extraction. We now take a closer look at both methods and their algorithmic details.

### *De novo* rate law generation

The main idea of the algorithm for *de novo* rate law creation is that the vast majority of biochemical reactions can be grouped into a limited number of categories (see Table 4 and Fig. 2). The rate law selection algorithm takes several features of the reaction into account in order to discriminate these categories. The most important sources of information in determining these categories are MIRIAM [27, 36–38] and SBO annotations [25] of model components, although models can also be evaluated if no such information is given. For each category, the algorithm either determines all kinds of principally applicable rate laws, or automatically selects the most suitable rate law. This decision process is performed by a prioritization function (Algorithm 1 in Additional file 2. The prioritization may require user interaction but can also be done as a fully automatic selection. During automatic selection, the algorithm ranks the reaction categories and applies the user-selected default rate law from the set *D* for the most significant category. The algorithm then equips all newly generated parameters with units in order to ensure consistency.

To go into additional detail (with the full algorithm described in the methods), the algorithm creates a submodel *M*^′^ that only comprises those reactions in the set *R* for which rate laws are to be created. All relevant model components, such as species, compartments, units, etc. are copied into this submodel. Operating on this trimmed copy of the full model has the advantage that changes of the algorithm do not affect the original data structure and can be easily disregarded. When creating this submodel, the algorithm also checks if fall-back units are defined for all components. This is crucial in order to avoid problems in later steps. Depending on which units are missing, it generates units for area, reaction extent, length, substance, time, and volume just as the default units in SBML Level 2 Version 5 [39] would be defined. All subsequent steps can hence assume that every model component has a defined unit.

The algorithm then iterates through all reactions within the submodel *M*^′^ and performs several preprocessing steps, before an appropriate type of rate law can be selected and created. The preprocessing steps (Algorithm 3 in Additional file 2 take as input the current reaction *r* together with an optional set of KEGG [26] compound identifiers and returns a set of characteristic features *R*_Features_ for this reaction. Passing KEGG identifiers, e.g., those listed in Table 1, to this procedure allows users to reduce the complexity of the generated equations by neglecting the contribution of the given compounds. The next step is to select all potentially applicable rate laws for reaction *r* based on its features *R*_Features_ and the user’s choice *e* that decides whether or not reactions without an explicit catalyst should be interpreted as being simplified representations of enzyme-catalyzed reactions. The result of this rule-based selection procedure is a set *K* of kinetic equations (Algorithm 4 in Additional file 2. From this set *K* one rate law *k* needs to be chosen, for which the algorithm then generates the actual equation and parameter set in submodel *M*^′^. This decision can either be done interactively or based on a set of priority rules and default rate laws *D* for the most general reaction categories.

When creating the actual rate law, an abstract syntax tree is assembled that takes the specific features of the reaction into account and also generates additional units for newly generated parameters as necessary (Fig. 3). Thereby, the algorithm avoids recreating already existing units. When the algorithm has processed all reactions, the modifications in submodel *M*^′^ need to be merged back into the original model *M*.

### Extraction of rate laws from SABIO-RK

The extraction of rate laws from the database SABIO-RK requires a model *M* (given as an SBML document) and query terms *Q* as input. All possible values for the query terms can be found in Additional file 1. Just like for the *de novo* creation of rate laws the algorithm can either process all reactions within the model or one particular reaction. To this end, the algorithm creates one query URL for each reaction, for which a rate law should be extracted from SABIO-RK. After obtaining a model *M*^′^ in form of an SBML document from SABIO-RK, the algorithm extracts all kinetic laws from *M*^′^ and tries to match all elements contained in a kinetic law to elements in the input model *M*. This matching is based on the MIRIAM annotations of model components and involves the search for one corresponding

- species in the local model for each species that participates in the kinetic law.
- compartment in the local model for each compartment addressed in the kinetic law (this can be the compartment of a participating species or the reaction itself can have a compartment assigned to it).
- reaction in the local model for each reaction in the kinetic law.
- species reference for each species reference in the kinetic law within each such identified local reaction. This species reference needs to refer to a species with an annotation similar to that of the species referenced by the species reference in the found rate law.

In this context, an annotation of two SBML elements is considered similar and hence these elements are considered a *match* if both have *controlled vocabulary terms* in common that are linked through qualifiers *has version* or *is*.

In the batch mode, the algorithm always selects the first kinetic law in the query results for which all elements can be matched to respective elements in the model. The algorithm adds this rate law to the reaction in the local model *M*. This merging involves

1. substituting all elements in the found kinetic law with the matched elements in the model; and
2. adding unit definitions, function definitions, global and local parameters contained in the kinetic law to the model.

When rate laws are obtained from SABIO-RK for individual reactions, the algorithm presents a list of all rate laws found for the given query to the user, who can then select the most appropriate equation. In cases when the selection of the first law with a successful matching does not lead to a satisfying outcome, the single reaction mode might yield better results.

### New features in SBMLsqueezer 2

The original version of SBMLsqueezer was developed as a plugin for CellDesigner [40]. Version 1.3 was later released with additional features [41]. The work presented here describes the expansion of the plugin to a full stand-alone software package alongside numerous algorithmic and technological advances. SBMLsqueezer 2 has been significantly refactored and provides a large number of new features, which are described in this section.

The number of supported rate equations has been greatly extended. For example, the program now includes all five modular rate laws described in recent work in kinetic equations for large-scale kinetic modeling [35]. Additionally, ten specific rate laws for gene-regulatory processes have been added:

- Hill-Hinze equation [42]
- Hill-Radde equation [43, 44]
- Linear additive network models (general form and Net*Gene*rator form) [45, 46]
- Non-linear additive network models (general form [46], Net*Gene*rator form [46], Vohradský’s equation [47, 48], Weaver’s equatio [49])
- S-systems [50]
- H-systems [51]

Table 5 lists all available rate laws.

Furthermore, SBMLsqueezer 2 contains a new module that automatically derives units for all new parameters. This feature is among the most complex capabilities of the program, because numerous aspects of the reaction need to be taken into account in order to ensure unit consistency. This comprises, for instance, the diverse fallback units in the model (depending on level/version combination of the SBML file), if the species involved are declared in concentration or amount units together with size and unit of their compartment, if reaction participants reside in different compartments, etc. The program needs to set units off against each other in order to cancel out terms, which is not trivial. Handling these issues manually can become an immensely time-consuming task, and thus automatic unit handling is one of the most valuable features of the package.

The algorithms are now entirely based on SBO- and MIRIAM annotations [25] rather than on CellDesigner-specific information. This change was necessary in order to create a stand-alone version of the program. SBMLsqueezer now not only understands annotations, it also annotates created objects (parameters, kinetic equations, units, etc.) with SBO terms and where possible also with MIRIAM controlled vocabulary terms. This new feature significantly increases quality and reusability of models.

**Fig. 4:**
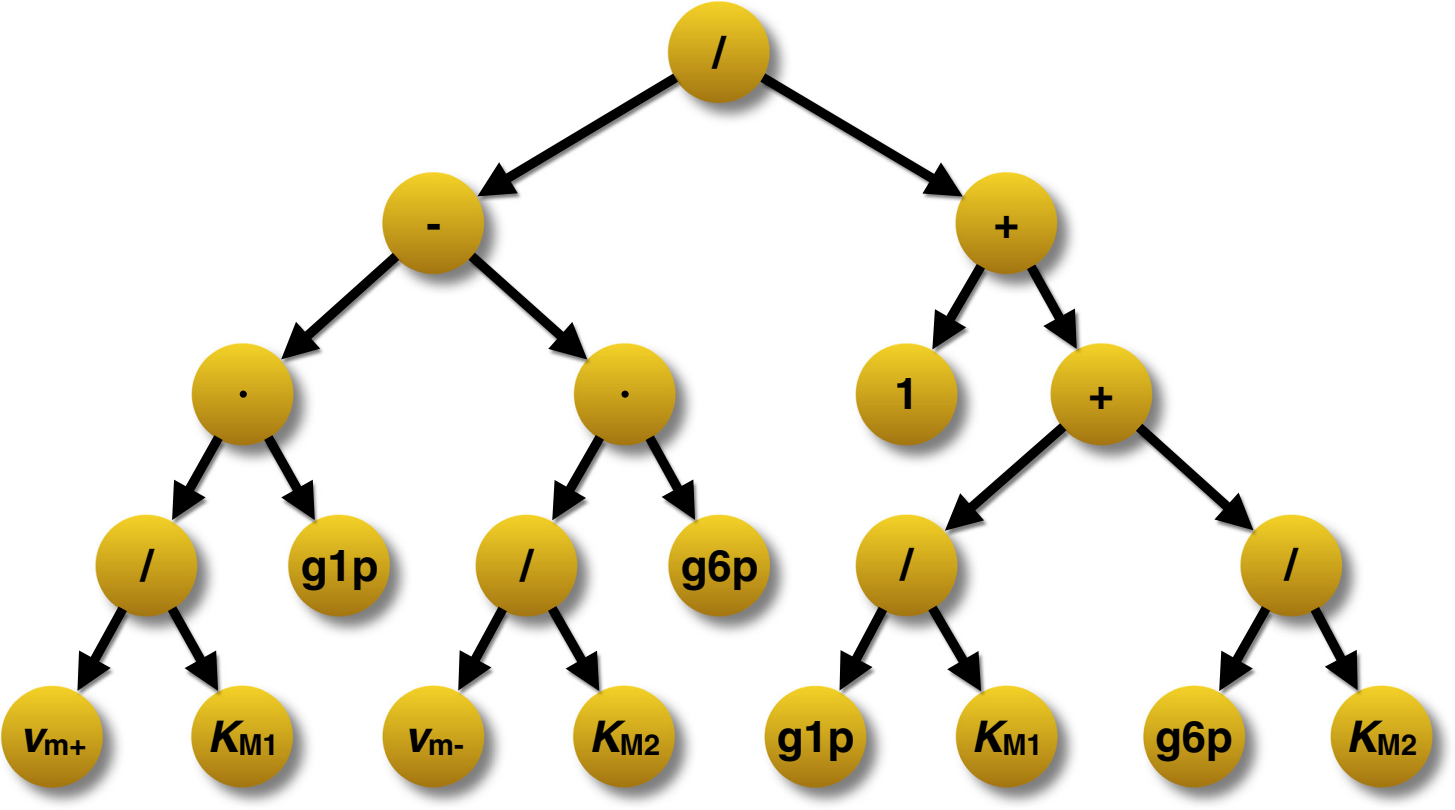
Abstract syntax tree for an enzymatic rate law for non-modulated unireactant enzymes (SBO:0000326). This tree has been constructed for the phosphoglucomutasereaction in model *i*JO1366 [69], in which \textsc{d}-glucose1-phosphate (g1p) is reversibly converted to \textsc{d}-glucose6-phosphate (g6p). The program internally constructs all equations in form of syntax trees, which can contain references to objects in the SBML document. In this example, the tree contains the parameters *v*_m+_ and *v*_m−_ for the forward and reverse maximal reaction velocity (both in mol ·*s* ^−1^), *K*_M1_ and *K*_M2_ for the Michaelis constants of the reactant and product (both in mol), the plain numerical value 1 (dimensionless in SBML Level 3, unit undefined for earlier versions of SBML), as well as references to the species g1p and g6p (both in mol). All internal nodes represent mathematical operators

A connection to the rate law database SABIO-RK [10] has been added and allows users to directly insert experimentally obtained rate laws from this database in addition of deriving generic equations. In particular, the access to SABIO-RK now enables the program to contribute to bottom-up knowledge-based model development as a complementary feature to the extended top-down rate law generation. The dialog for access to SABIO-RK is similar to the online database service and uses the annotation of the reaction and its components to identify the best match in SABIO-RK. Then, rate laws, parameters, units, and annotations are transferred from SABIO-RK to the local model. Rate law generation and extraction from SABIO-RK can be performed for individual reactions (Fig. 4) or for the entire model in a single step. Algorithms 1 and 2 in Additional file 2 show how both methods of the program interact. SBMLsqueezer facilitates the rate law prioritization by presenting an equation preview, which assists the user to make this decision.

The CellDesigner plugin mode is now only one of many ways to use the program. SBMLsqueezer can now be used as *a* online program (via a Galaxy webservice as well as a Java™ Web Start program), *b* stand-alone tool via graphical user interface or command-line, *c* plugin for CellDesigner, *d* Garuda gadget, and *e* through its API in complex workflows and algorithms.

The comprehensive Users’ Guide (see Additional file 1) gives code examples and details how to benefit from all program features in each described environment.

Additionally, SBMLsqueezer can now deal with all levels and versions of the SBML format, from Level 1 Version 1 through Level 3 Version 1, whereas CellDesigner is restricted from SBML Level 1 Version 2 up to Level 2 Version 4.

## Conclusions

SBMLsqueezer 2 is a mature and stable application that can be applied in diverse ways. SBMLsqueezer can easily be integrated into versatile model construction workflows. As an example, draft models can be first obtained from the model database BiGG [52] or be generated with the program KEGGtranslator [53, 54]. Second, SBMLsqueezer can be used to generate kinetic equations for all reactions in the draft models. Finally, the program SBMLsimulator [55, 56] can estimate the unknown values of the model parameters by fitting the models to experimental data. We note that while the *de novo* creation method guarantees that a rate law can always be created, the extraction from SABIO-RK depends on existing biochemical data and might therefore not always yield results. A combination of both methods would therefore be recommended to quickly create an initial kinetic model. The Users’ Guide (Additional file 1^2^) explains in detail how to utilize all program functions and provides several sample use-cases.

While SBMLsqueezer 2 has made substantial progress in addressing the challenges of automated rate law generation, there are still many possible extensions. For example, in order to support mathematical equations for transition functions in logical models, it would be possible to derive similar methods based on the SBML extension for qualitative models [57, 58]. Furthermore, as network reconstructions continue to expand beyond metabolism, more detailed rate laws applicable to transcription and translation modeling may continue to be implemented as part of the capabilities of the package. The advantages of SBMLsqueezer lie in the interfaces to established systems biology databases and data standards, features that will remain useful even as the preference in rate laws shift as the field develops.

The software has already been proven to be useful in diverse applications. Examples include synthetic biology [59], mechanistic modeling of methicillin-resistance in bacteria [60], explaining dynamic damage response in human fibroblasts after exposing them to *γ* radiation [61], drug discovery [62] and drug effect modeling [63, 64], and modeling complex signaling cascades [65]. Endeavors such as the path2models project [66] have additionally demonstrated the usefulness of automated rate law assignment. Furthermore, the internal data structure of SBMLsqueezer has become a separate large-scale community effort leading to the development of JSBML [67], which is now a separate project used by numerous other research groups. Utilizing SBMLsqueezer 2, rigorous kinetic modeling efforts involving complex try-and-evaluate cycles, for example where the most suitable rate law for a certain reaction needs to be identified in repeated simulation runs [67], become increasingly manageable tasks.

## Availability and requirements

Program, source code, and documentation can be obtained under the terms of the GPL version 3 from the website. *Project name:* SBMLsqueezer *Project homepage:*http://www.cogsys.cs.uni-tuebingen.de/software/SBMLsqueezer/*Contact:*sbmlsqueezer@googlegroups.com*Operating systems:* Platform independent, i.e., for all systems for which a Java^TM^ is available. *Programming language:* Java™ *Other requirements* Java™ Runtime Environment (JRE) 1.6 or above *License:* GNU General Public License (GPL) version 3 *Any restrictions to use by non-academics:* None

## Endnotes

^1^http://opencobra.github.io/MASS-Toolbox/

^2^ Up-to-date versions of the Users’ Guide can be found at the project website.

## Additional files

Additional file 1
**Users’ Guide.** This PDF file contains a comprehensive description of the program SBMLsqueezer. It includes details about all of its functions of the GUI, command-line options, example use cases, and source code examples for using SBMLsqueezer as an API library. (4239 Kb)

Additional file 2
**Algorithms of SBMLsqueezer.** This PDF document explains all main algorithms of SBMLsqueezer in detail using pseudocode and additional descriptions. (141 Kb)

## Abbreviations

API: Application Programming Interface
asRNA: Antisense RNA
BioPAX: Biological pathway exchange language
FAQ: Frequently asked questions
GPL: GNU General Public License
GUI: Graphical User Interface
HTML: Hypertext Markup Language
ID: Identifier
IDE: Integrated development environment
JDK: Java™
development kit; JRE: Java™
Runtime Environment; JVM: Java™
Virtual Machine; JAR: Java™
Archive; LGPL: GNU Lesser General Public License
KEGG: Kyoto Encyclopedia of Genes and Genomes
MIRIAM: Minimal Information Required In the Annotation of Models
mRNA: Messenger RNA
PDF: Portable Document Format
PGM: Phosphoglucomutase
RESTful: Representational State Transfer
RNA: Ribonucleic Acid
SBGN: Systems Biology Graphical Notation
SBML: Systems Biology Markup Language
SABIO-RK: System for the Analysis of Biochemical Pathways –
Reaction Kinetics; SBO: Systems Biology Ontology
URL: Uniform Resource Locator
XHTML: Extended HTML
XML: Extended Markup Language
*A*: Set of activators and stimulators
*C*: Set of non-enzyme catalysts
*D*: Set of default rate laws for each category
*e*: Boolean variable that is true if all reactions should be assumed enzyme-catalyzed
*E*: Set of enzymes
*I*: Set of inhibitors
*k*: Selected rate law for the current reaction
*K*: Set of potentially applicable rate laws for a reaction
M: Model
*M*^′^: Trimmed model copy
$\vec {p}$: Vector of model parameters
*p*_allPolypeptides_: Boolean variable that is true if all products represent polypeptides
*p*_Stoichiometry_: Accumulated stoichiometry value of all products
*P*_*r*_: Set of products
*Q*: Set of query terms for SABIO-RK
*r*_allGenes_: Boolean variable that is true if all reactants represent genes
*r*_allRNA_: Boolean variable that is true if all reactants represent RNA molecules
*r*_Stoichiometry_: Accumulated stoichiometry value of all reactants
*R*: Set of all reactions in a model
*R*_Features_: Set of characteristic reaction features
*R*_*r*_: Set of reactants
**S**: Stoichiometric matrix
*t*: Time
$\vec {v}$: Vector of reaction velocities
**W**: Modulation matrix
*x*: Species from vector $\vec {x}$
