# Supplementary material for: SBMLsqueezer 2: context-sensitive creation of kinetic equations in biochemical networks
Source: BMC Syst Biol. 2015 Oct 9;9:68. doi: 10.1186/s12918-015-0212-9 (PMC4600286; doi:10.1186/s12918-015-0212-9)
Supplement: Additional file 1 — Users’ Guide. This PDF file contains a comprehensive description of the program SBMLsqueezer. It includes details about all of its functions of the GUI, command-line options, example use cases, and source code examples for using SBMLsqueezer as an API library. (4239 Kb) [file 12918_2015_212_MOESM1_ESM.pdf]

EBERHARD KARLS
UNIVERSITÄT
TÜBINGEN

MATHEMATISCH-
NATURWISSENSCHAFTLICHE
FAKULTÄT

Users' Guide

SBMLsqueezer 2.1

A context-based generator of kinetic equations

Andreas Dräger[1,2,*] Daniel C. Zielinski[1] Roland Keller[2]
Matthias Rall[2] Johannes Eichner[2] Bernhard O. Palsson[1]
Andreas Zell[2]

Monday 10th August, 2015

Institutional affiliations:
[1]Systems Biology Research Group, University of California, San Diego, La Jolla, CA, USA
[2]Center for Bioinformatics Tuebingen (ZBIT), University of Tuebingen, Tübingen, Germany

[*]Corresponding author: andraeger@eng.ucsd.edu

The tool SBMLsqueezer facilitates the task of assigning kinetic equations to reactions within biochemical network models that are given in SBML format. It analyzes each reaction of interest, selects applicable equations, and derives appropriate units of all parameters contained in these equations. If possible, components that SBMLsqueezer adds to your model are annotated with Systems Biology Ontology (SBO) and Minimal Information Required In the Annotation of Models (MIRIAM) tags. Besides *de-novo* creation of rate equations, SBMLsqueezer also offers online access to the reaction kinetics database System for the Analysis of Biochemical Pathways – Reaction Kinetics (SABIO-RK), where you can extract experimentally determined rate laws for your model. In both modes (*de-novo* creation and database lookup), you can assign kinetic equations to all reactions of the model in one single step, or select individual reactions of interest. Several settings allow you to customize the behavior of the program. In particular, all choices made by SBMLsqueezer can be changed or influenced. The program can be used in multiple ways: as a plug-in for the program CellDesigner, as a gadget for Garuda, as a stand-alone tool via its graphical user interface, or as a command-line based tool. Furthermore, the application programming interface allows you to integrate SBMLsqueezer as an equation generating core into your end-user application. An export function based on the integrated program SBML2LaTeX allows you to generate a comprehensive model report for scientific writing or further processing.

Contents

1 Introduction **1**
1.1 Overview 1
1.2 General program features 3

2 Installation **5**
2.1 Requirements 5
2.2 Stand-alone application 6
2.3 Plug-in for CellDesigner 8
2.4 Integration into Garuda 9
2.5 Online program 9
2.6 Java™ Web Start 9

3 How to get started **10**
3.1 Starting the application 10
3.2 Adjusting the preferences 11
3.3 Open a model 13
3.4 Equation generation one by one 15
3.5 Generate kinetic equations in a single step 17
3.6 Extraction of rate laws from SABIO-RK 20
3.7 Viewing and saving the results 24
3.8 Interaction with further Garuda gadgets 29
3.9 Using SBMLsqueezer as an online program 30

4 Advanced program features **33**
4.1 Using SBMLsqueezer as an equation generator library 33
4.2 Querying SABIO-RK and merging the results into a local SBML document 38
4.3 Command-line arguments 40

5 Supported rate laws **59**
5.1 Rate laws for metabolic processes 59
5.2 Rate laws for gene-regulatory processes 60

6 FAQ and troubleshooting 62

7 License 64
7.1 Disclaimer 64
7.2 Included third-party libraries 64

8 Acknowledgments 65
8.1 Core developers 65
8.2 Principal Investigators 65
8.3 Alumni 65
8.4 Collaborators and partners 66

A Release Notes 67
A.1 Version 1.0 67
A.2 Version 1.1 67
A.3 Version 1.2 68
A.4 Version 1.2.1 70
A.5 Version 1.3 71
A.6 Version 2.0 76
A.7 Version 2.0.1 78
A.8 Version 2.1 78

B Acronyms 80

Bibliography 82

Index 89

Figures

3.1 Basic configuration 11
3.2 Rate law selection 12
3.3 SABIO-RK search options 13
3.4 SABIO-RK search preferences 14
3.5 LaTeX options 15
3.6 Reaction context menu in CellDesigner 16
3.7 Generating a kinetic equation for a single reaction 16
3.8 Launching SBMLsqueezer in CellDesigner 18
3.9 Generating kinetic equations in one single step with the kinetic law wizard 18
3.10 Results of the kinetics wizard displayed in a table 19
3.11 Changing selected rate laws in the kinetics wizard 19

3.12 Starting the SABIO-RK wizard . . . 20
3.13 Selection of reactions in the SABIO-RK wizard . . . 21
3.14 Selection of search terms for SABIO-RK . . . 21
3.15 Removal of search terms for SABIO-RK . . . 22
3.16 Window with kinetic equations found for all reactions . . . 22
3.17 Summary of necessary changes . . . 23
3.18 Starting the SABIO-RK application for a selected reaction . . . 24
3.19 Selection of search terms . . . 25
3.20 Window with two search terms . . . 25
3.21 Window with kinetic equations found for a single reaction . . . 26
3.22 Window for matching elements in the equation to model elements . . . 27
3.23 Summary of necessary changes . . . 27
3.24 Viewing SBMLsqueezer's results . . . 28
3.25 Selecting a Garuda gadget . . . 29
3.26 Starting the online version of SBMLsqueezer . . . 30
3.27 Adjusting preferences in the online version . . . 31
3.28 Obtaining the results from the online version . . . 32

Tables

5.1 KEGG Identifiers (IDs) of small molecules and ions . . . 61

Listings

2.1 Example Bash script that launches SBMLsqueezer with a libSBML back-end . . . 7

4.1 Generating rate laws for all reactions in a model via the API of SBMLsqueezer 2.1 34
4.2 Loading preferences in SBMLsqueezer 2.1 . . . 35
4.3 Initializing SBMLsqueezer 2.1 with a libSBML back-end . . . 36
4.4 Assignment of a kinetic law to a reaction via the API of SBMLsqueezer 2 . . . 37
4.5 SABIO-RK query for the reactions of a model . . . 38
4.6 Calling the SABIO-RK wizard . . . 39
4.7 Launching SBMLsqueezer from the command-line . . . 40
4.8 Launching SBMLsqueezer from the command-line with increased heap size . . . 41
4.9 Launching SBMLsqueezer with an alternative language pack . . . 41

A.1 Usage of SBMLsqueezer 1.3 via its API . . . 72

1 Introduction

The program SBMLsqueezer is a versatile tool for the assembly and assignment of complex kinetic equations of reactions and processes in biochemical networks (Dräger *et al.*, 2008, 2010; Dräger, 2011). The purpose of SBMLsqueezer is hence to assist you when creating kinetic equations for a biochemical network, whose structure is already defined. SBMLsqueezer can add missing information, such as undefined initial concentration values or set some reacting species that represent genes to the boundary of the reaction system. But its main purpose is the creation of rate laws, parameter objects, and their units. The integrated SBML2LaTeX can create an exhaustive model report that highlights all model features (Dräger *et al.*, 2009b). A list of currently implemented rate laws that can be used for the *de novo* creation of kinetic laws can be found in chapter 5 on page 59.

1.1 Overview

The data format understood by SBMLsqueezer is the Systems Biology Markup Language (SBML)[1] in all of its levels and versions (Hucka *et al.*, 2001, 2003a,b, 2007, 2008, 2010, 2015; Finney and Hucka, 2003; Finney *et al.*, 2006). When using SBML Level 1 (not recommended), all generated equations are stored in form of infix formula strings. For all other versions of SBML, the generated equations are stored in form of MathML (Buswell *et al.*, 1999) expressions.

Kinetic equations contain a set of parameters, whose units must be derived to ensure that the overall equation can be interpreted in units of the extent of the respective reaction per time units. To give an example for the most common scenario, you can assume that the units of the rate law will simplify to a substance unit per time unit, for instance, mole per second. Other constellations, however, are possible, too. When using SBMLsqueezer you do not have to care much about units because the program does this for you. Since Level 3, the MathML subset understood by SBML has been extended and also supports units for actual numbers (integers or real values, which are not parameter values in this sense). When using a model in this more recent format, SBMLsqueezer will also assign units to numbers where necessary.

The program also annotates all created equations and parameters with appropriate terms from the SBO[2] (Systems Biology Ontology) and also with MIRIAM-compliant[3] controlled vocabulary terms (Minimal Information Required In the Annotation of Models), where appropriate (Le Novère *et al.*, 2005; Le Novère *et al.*, 2006; Laible and Le Novère, 2007; Courtot *et al.*, 2011).

[1] Various information about SBML can be found at `http://sbml.org`.
[2] For more information about SBO see `http://www.ebi.ac.uk/sbo/main/`.
[3] The MIRIAM registry is described at `http://co.mbine.org/standards/miriam`.

The selection of the kinetic equations is done automatically by the program, where several settings allow users to influence the algorithm's choice. In many cases, SBMLsqueezer suggests multiple equations for the same process, from which the user can choose as desired. This behavior of the program is important to ensure that only appropriate equations can be selected at any time.

In order to decide, which equations can be applied to a reaction of interest, SBMLsqueezer analyzes several properties, such as the number and type of reactants, products, and modifiers, if the reaction is reversible, etc. Here, reaction of interest means that the user can either select individual reactions from a larger model and equip these with kinetic equations, or rate equations can be created in one go for the entire model. Thereby, already existing equations can either be kept or overwritten, depending on your choice. You can influence and change all choices of the program, even in the single-step-mode.

If you are connected to the Internet, you can also use SBMLsqueezer to extract experimentally determined kinetic equations from the rate law database SABIO-RK[4] (Wittig *et al.*, 2006; Rojas *et al.*, 2007; Krebs *et al.*, 2007; Wittig *et al.*, 2012). To this end, SBMLsqueezer provides several settings, such as the organism, temperature, or pH value under which the reaction's rate was determined. Again, you can extract equations from SABIO-RK for an entire network in one single step, or individually select reactions of interest.

The program itself can be customized and used in several ways. It remembers your last opened files, the window's size, your rate law selection and so forth. You can run it as a stand-alone tool, or as a plug-in for the well-known program CellDesigner[5] (Funahashi *et al.*, 2003, 2006, 2007, 2008). By default, the stand-alone version is based on the JSBML back-end (Dräger *et al.*, 2011; Rodriguez *et al.*, 2015), but you can also use libSBML (Bornstein *et al.*, 2008) as its SBML back-end instead if this library's more elaborated off-line model validation matters.

If you neither like to download SBMLsqueezer, nor to install any software on your local computer, you can even use SBMLsqueezer as a Galaxy-based web service[6] Goecks *et al.* (2010). This web-service runs on the servers of the University of Tuebingen and therefore do not require any local installation. In this setting, you can benefit from Galaxy's capability to easily combine SBMLsqueezer with other programs in a more complex work-flow. You can, for instance, convert a BioPAX file to SBML using the also included program BioPAX2SBML (Büchel *et al.*, 2012) and populate the model with kinetic equations afterwards. In this way, SBMLsqueezer integrates well in standard exchange formats in systems biology (Dräger and Palsson, 2014). Another option would be to launch it as a Java™ Web Start application directly from your web browser[7]. In this case, your browser will download the executable program SBMLsqueezer as a temporary file and run it locally on your computer.

In addition, SBMLsqueezer implements the Garuda specification and can therefore also be used as a gadget within this powerful framework for systems biology (Ghosh *et al.*, 2011). In this

[4] You can access SABIO-RK at `http://sabio.h-its.org`.

[5] For more information about CellDesigner see `http://www.celldesigner.org`.

[6] Galaxy web service available at `http://webservices.cs.uni-tuebingen.de`.

[7] You can use SBMLsqueezer as a Java™ Web Start application by clicking on `http://www.cogsys.cs.uni-tuebingen.de/software/SBMLsqueezer/downloads/SBMLsqueezer.jnlp`.

configuration, the output of tools that assist you to create your models, such as KEGGtranslator (Wrzodek *et al.*, 2011, 2013) can directly be piped as the input to SBMLsqueezer. Furthermore, SBMLsqueezer's output can be forwarded to further programs, such as SBMLsimulator (Keller *et al.*, 2013; Dörr *et al.*, 2014) for further analysis.

Finally, the command-line mode of SBMLsqueezer provides all capabilities of the program without any restriction. Furthermore, the fully accessible Application Programming Interface (API) can be used to integrate SBMLsqueezer as an equation generating core into your end-user program. You can hence equip a large number of files with kinetic equations without the need to open each file in a Graphical User Interface (GUI). The usefulness of this approach has recently been demonstrated as part of the path2models project[8], in which more than 142,000 SBML models have been processed with SBMLsqueezer (Büchel *et al.*, 2013).

For the documentation of your model, SBMLsqueezer includes the program SBML2LaTeX[9], which generates a comprehensive report of your model, including a detailed description of all components and equations (Dräger *et al.*, 2009b; Dräger *et al.*, 2010). These reports can be very handy to support scientific writing, because you can easily copy the formulas into your scientific paper.

1.2 General program features

Depending on in which variant you use SBMLsqueezer and your preferences (see section 3.2 on page 11), SBMLsqueezer can

- ✔ generate kinetic equations for all reactions in your model, or only for those reactions that are currently lacking a rate law. This gives you the option to replace existing rate laws with new ones. Thereby, you can define a number of arbitrary chemicals (usually small molecules or ions) that you like to exclude from the rate law generation process in order to keep the generated equation simple. The program offers you a large variety of generic and specific rate laws for several standard cases and allows you to create all rate laws in a reversible manner if desired.

- ✔ detect reactive species, whose annotation indicates that these are genes. Since usually the concentration or amount of genes does not change, SBMLsqueezer offers you to set the boundary condition of these species to `true`. This means that even if these species participate in reactions, their amount or concentration will always remain constant, irrespective of their participation in reactions.

- ✔ assume that all reactions in your network are enzymatically catalyzed and hence change the selection of rate laws. This means that even reactions without explicit catalyst can be modeled with an enzymatic rate law. Hence, a simplified model structure can be recognized

[8] You can access and download all pathway models of the path2models project from `http://www.ebi.ac.uk/biomodels-main/path2models`.

[9] Download SBML2LaTeX at `http://www.cogsys.cs.uni-tuebingen.de/software/SBML2LaTeX/`.

by SBMLsqueezer and interpreted as a more complex structure. For catalyzed reactions, you have the choice of a large variety of species types to be interpreted as enzymes.

✔ define the units of all species and compartments if necessary and derive the units for all newly created local and global parameters and numbers in order to ensure unit consistency of the entire model. Thereby you can choose if species should be brought to amount or concentration units. This influences if in rate equations division by the compartment sizes or multiplication with the compartment will be performed where this is necessary.

✔ check the model for global and local parameters as well as unit definitions that are never used and addressed. SBMLsqueezer can automatically remove these from the model, i.e., perform *model cleaning*.

✔ import experimentally determined rate equations from SABIO-RK and include them into your model. To this end, it offers you several options to make the lookup of rate laws as precise as possible and to narrow down the database entries as much as possible.

✔ equip your model with default values where no values are defined. For instance, if it was forgotten to define the spatial dimensions or the size of a compartment, SBMLsqueezer will insert a default value for you. The same is also done for species and all newly created (local) parameters.

✔ summarize all features of the model in an exhaustive LaTeX-based model report. To this end, SBMLsqueezer brings with it a full version of the model documentation tool SBML2LaTeX including all of its options.

This document is intended to guide you through installation and use of SBMLsqueeer by introducing its various interfaces to you. In the following, we will describe how you can benefit from the features of SBMLsqueezer and how to customize the program. The next chapter describes how you can install and access SBMLsqueezer on your operating system (see p. page 5). Chapter 3 introduces the GUI of the stand-alone version of SBMLsqueezer to you and explains how to access all of its functions. If you are interested to run SBMLsqueezer in a batch or shell script, the command-line interface will be interesting for you, which is described in section 4.3 on page 40.

Just as every other scientific software, also SBMLsqueezer is work in progress. If you encounter any difficulties or bugs, please do not hesitate to contact the mailing list, ✉ `sbmlsqueezer@googlegroups.com`.

2 Installation

To obtain a local copy of SBMLsqueezer, you can download it in form of a Java™ Archive (JAR) file from the project's website[1].

In the most common scenario, you might want to launch the program as a stand-alone tool and access its GUI. To do so, start SBMLsqueezer with a simple double click on the icon of the downloaded JAR file. Provided that a Java™ Virtual Machine (JVM) is installed on your system (see section 2.1.2 on the next page), you will see the main window of SBMLsqueezer as soon as the splash screen has finished.

2.1 Requirements

SBMLsqueezer can be be used in multiple ways, depending on your preferences:

- As a stand-alone tool
  - via its GUI (see chapter 3 on page 10)
  - via its command-line interface (see section 4.3 on page 40)

  In both cases, you can choose between JSBML or libSBML as your backend for SBML (see section 2.2 on the following page).

- As a plug-in for CellDesigner (see section 2.3 on page 8)
- As a gadget for Garuda (see section 2.4 on page 9)
- As an online program embedded in the Galaxy web service environment Goecks *et al.* (2010)
- As a Java™ Web Start application
- As a rate law core in an end-user application via its API

Depending on how you like to use SBMLsqueezer, different requirements must be fulfilled before launching the program for the first time.

Note that the extraction of kinetic equations from SABIO-RK requires an active Internet connection. For this program features, the security settings of your operating system must also allow SBMLsqueezer to establish a connection to this online database.

[1] Project website of SBMLsqueezer: `http://www.cogsys.cs.uni-tuebingen.de/software/SBMLsqueezer/`.

When launching SBMLsqueezer, it will connect to the program homepage in order to check if a newer version of the program is available. This feature can also only be used if an Internet connection is available and if your operating system allows SBMLsqueezer to establish such a connection. Note that no further information is transmitted. SBMLsqueezer only compares the version number of the local program with the version number of the latest release. If you do not want to use this feature, you can switch it off. See section 4.3 on page 40 for instructions how to do so.

2.1.1 Hardware

With at least 1 GB main memory, you should be able to perform most tasks without any problem. For large models, you should have at least 2 GB of main memory. An active Internet connection is required for some program features, but not mandatory to run the program.

2.1.2 Software

SBMLsqueezer is entirely implemented in Java™ and runs on any operating system, where a suitable Java™ Virtual Machine (JVM), Java™ Development Kit (JDK) version 1.6 or newer, is installed. For instructions how to obtain an up-to-date Java™ Virtual Machine (JVM) for your system, see, for example, the Java™ SE download page[2].

SBMLsqueezer has successfully been tested with

- Microsoft Windows 7 Professional (32 bit with SP1 as well as 64 bit with and without SP1),
- Mac OS X (versions 10.8.2 through 10.10.4), and
- Ubuntu Linux (version 12.04, 64 bit).

See chapter 6 on page 62 if you encounter any problems.

2.2 Stand-alone application

Besides what is explained above, no further requirements are necessary if you like to use SBMLsqueezer as a stand-alone application. The program SBMLsqueezer does not have to be installed in order to be executed. Just copy the JAR file of SBMLsqueezer to your preferred path on your hard-disk to launch the application. On Mac OS X, you may like to copy the JAR file into your `Macintosh HD ▸ Applications` folder. On Microsoft Windows systems, the preferred position for the JAR file could be, for instance, `C: ▸ Program Files`. For Linux, we propose to copy the JAR file to the `opt` folder.

[2] http://www.oracle.com/technetwork/java/javase/downloads/

Using the JSBML back-end

No special actions are necessary if you like to use JSBML, because this is the default and JSBML is already included in the JAR file that you have downloaded.

Launching SBMLsqueezer with libSBML as back-end

Follow the installation instructions of the Java™ binding for libSBML for your platform, which you can find at the website of libSBML[3]. Please note that when using SBMLsqueezer with a libSBML backend, it requires libSBML version 5.9.0. On some platforms, you may have to define an environment variable pointing to the installation directory of libSBML before being able to use its Java™ binding. On the most Unix and Linux platforms, this variable is called `LD_LIBRARY_PATH`. On Mac OS X, you should instead define the variable `DYLD_LIBRARY_PATH`. Follow the instructions at libSBML's website[4] before launching SBMLsqueezer. On Windows you should add the libSBML directory to the `PATH` environment in the Control Panel. The following script can help you to to run SBMLsqueezer on your Unix platform:

```
#!/bin/bash

VM_ARGS="-Xms32M␣-Xmx512M␣-Djava.library.path="
# The following lines depend on your system's configuration;
# so here is just an example:
VM_ARGS="${VM_ARGS}\
/usr/lib/jvm/java-6-sun/jre/lib/i386/client:\
/usr/lib/jvm/java-6-sun/jre/lib/i386:\
/usr/lib/jvm/java-6-sun/lib:\
/usr/local/lib"
#:[path to xerces]/xerces/lib
CLASS_PATH="/usr/local/share/java/libsbmlj.jar:\
[path␣to␣]SBMLsqueezer_v2.1.jar"

# Set the environment variable; under Linux or most Unix systems this is
LD_LIBRARY_PATH="${LD_LIBRARY_PATH}:/usr/local/lib"
# On Mac OS you have to use the following code instead:
#DYLD_LIBRARY_PATH="${DYLD_LIBRARY_PATH}:/usr/local/lib"

# Start SBMLsqueezer using the command-line options:
MAIN_CLASS=org.sbml.squeezer.SBMLsqueezer
java ${VM_ARGS} -cp ${CLASS_PATH} ${MAIN_CLASS} --try-loading-libsbml=true\
[further options]
```

Listing 2.1: Example Bash script that launches SBMLsqueezer with a libSBML back-end

[3] `http://sbml.org/Software/libSBML`

[4] For information about how to execute a libSBML-based Java™ application, see the section about Java at `http://sbml.org/Software/libSBML/docs/cpp-api/libsbml-accessing.html`

In the example above the arguments for the Java™ Virtual Machine (JVM) define an initial heap space of 32 MB (-Xms32M) and a maximal heap size of 512 MB (-Xmx512M). For more information about the program usage from the command line see section 4.3.1 on page 40.

2.3 Plug-in for CellDesigner

If you like to use SBMLsqueezer as a plug-in for the popular graphical model editor CellDesigner (Funahashi *et al.*, 2003, 2006, 2007, 2008), you can do this by following four installation steps:

1. Download and install CellDesigner from the project's website[5].
2. Locate, where your copy of CellDesigner is installed.
3. Replace the file 🗀 exec ▸ celldesigner.jar with the updated file celldesigner.jar that you can obtain from http://www.celldesigner.org/~funa/GSoC/. On this website you can also find further details about the plug-in installation process.
4. Open the 🗀 plugin folder in your installation of CellDesigner and copy the file SBMLsqueezer_v2.1.jar into this folder.
5. Open the 🗀 lib folder in your installation of CellDesigner and replace the JSBML JAR file that is redistributed by CellDesigner with the JAR file that you can download at http://www.cogsys.cs.uni-tuebingen.de/software/SBMLsqueezer/downloads/jsbml-1.1-a1-incl-libs.jar.

Some more information about this installation procedure: Depending on your operating system, CellDesigner will be installed in different folders. In Microsoft Windows it is usually 🗀 C: ▸ Program Files ▸ CellDesigner4.4. In Linux you might find it in 🗀 opt ▸ CellDesigner4.4, and in Mac OS X CellDesigner will be installed under 🗀 Macintosh HD ▸ Applications ▸ CellDesigner4.4, where 4.4 is the version number with which SBMLsqueezer 2.1 has been tested. Note that the version numbers 4.4 and 2.1 of CellDesigner and SBMLsqueezer might change in the future, but the procedure will remain the same.

When replacing CellDesigner's version of JSBML with the one downloaded from the download site of the SBMLsqueezer project, make sure that the name of the new JSBML file will be identical to the name of the JSBML file distributed with CellDesigner. Otherwise, you will have to change some start script of CellDesigner. Even though this is not difficult and can be done on each operating system, we prefer to avoid that for the sake of simplicity. Alternatively, you can also download JSBML from the official sourceforge page. However, you must make sure that the downloaded JAR file includes the package 🗀 org.sbml.jsbml.celldesigner. Without this package, SBMLsqueezer cannot be launched in CellDesigner. Another possible solution is to checkout the

[5] http://celldesigner.org

development trunk of JSBML and run the antscript by yourself, thus building the latest version of JSBML. If you like to go this way, please read the documentation about JSBML[6] first.

It should be noted that SBMLsqueezer might produce different results when using it as a plug-in of CellDesigner from the results of all other ways to use the program. The reason is that CellDesigner models can contain a rich tool-specific annotation, which can only be understood by SBMLsqueezer in the plug-in mode for this program. In contrast, all other variants of SBMLsqueezer described herein depend on SBO terms to indicate the roles of modifiers (e.g., enzymatic catalysts) when it is not used as a plug-in for CellDesigner. Again, the reason is that the CellDesigner plug-in has different ways to receive this information. Applying SBMLsqueezer to a model exported from CellDesigner and opened in the stand-alone version might therefore yield different results compared to when directly running it as a plug-in from CellDesigner.

2.4 Integration into Garuda

When downloading Garuda, a functioning copy of SBMLsqueezer will be included. Hence, no special installation steps are required. Just follow the instructions of how to install Garuda[7] (Ghosh *et al.*, 2011).

2.5 Online program

In order to use SBMLsqueezer as an online program, it is necessary to install a recent web browser for your specific operating system, such as Mozilla Firefox, Google Chrome, Apple Safari, Microsoft Internet Explorer, Opera, or any other browser that you prefer and that is able to render up-to-date web sites. See the documentation of Galaxy web services[8] (Goecks *et al.*, 2010), on which the online version of SBMLsqueezer is based, for precise software requirements.

When using the online version, no specific installation of SBMLsqueezer is necessary. Note, however, that an active Internet connection is essential in order to use SBMLsqueezer as an online program. Just use your regular web browser and go to `http://webservices.cs.uni-tuebingen.de` to use the program. See section 3.9 on page 30 for further information.

2.6 Java™ Web Start

Click `http://www.cogsys.cs.uni-tuebingen.de/software/SBMLsqueezer/downloads/SBMLsqueezer.jnlp` in order to launch SBMLsqueezer as a Java™ Web Start application. Given that you have Java™ installed on your operating system and enabled in your web browser, SBMLsqueezer will be directly launched on your local computer without installation.

[6] `http://sbml.org/Software/JSBML/docs`
[7] `http://www.garuda-alliance.org/`
[8] `http://galaxyproject.org`

3 How to get started

In this chapter you will learn how the GUI of the program SBMLsqueezer is designed and intended to work, how you can launch, configure, and use the program. We will systematically go through all program features and explain what can be done and how. Where possible, keystroke combinations will be introduced that can be used to simplify the work with the program. All special scenarios of the diverse ways of using SBMLsqueezer (as a stand-alone tool, online program, plug-in in CellDesigner, Java™ Web Start application, as a gadget of Garuda, etc.) will be highlighted and discussed.

More experienced users might want to skip several parts of this chapter and be more interested in the description of the command-line interface (see section 4.3 on page 40) or examples for how to use the API (see section 4.1 on page 33) in custom applications.

3.1 Starting the application

If you downloaded a ZIP file, you need to unzip it before starting the application. Launch SBMLsqueezer by double-clicking on the Java™ icon, which might look like the image next to this text, in a file browser of your operating system. This will start the GUI (see chapter 3 for further details).

You can also start SBMLsqueezer from the command line. Depending on your preference and your operating system, it might be helpful for you to write a short shell or bash scripts for starting the application. You might name this, for instance, `start.sh` for Linux or Mac OS X, and `start.bat` for Microsoft Windows. Within those scripts you could specify several command-line options (see section 4.3 on page 40) and hence customize the behavior of SBMLsqueezer. How to do that is described in section 4.3.1 on page 40. If you are using libSBML as your SBML parsing engine, writing such a script is highly recommended. You can find a sample script for this purpose in section 2.2 on page 7.

If you are using SBMLsqueezer as a plug-in for CellDesigner, just open a model that you like to edit. You can then launch all functions of SBMLsqueezer by clicking on one of the options within the menu Plugin > SBMLsqueezer 2.1 in CellDesigner.

To start SBMLsqueezer in Garuda, simply double-click on SBMLsqueezer's program icon (see the symbol that is printed next to this text). Alternatively, you can also launch SBMLsqueezer there by sending an SBML file to it from any other Garuda gadget. For more information about that, see the documentation of Garuda.

When using SBMLsqueezer as an online program, just open a browser of your choice and go to `http://webservices.cs.uni-tuebingen.de` and select the tool SBMLsqueezer in the

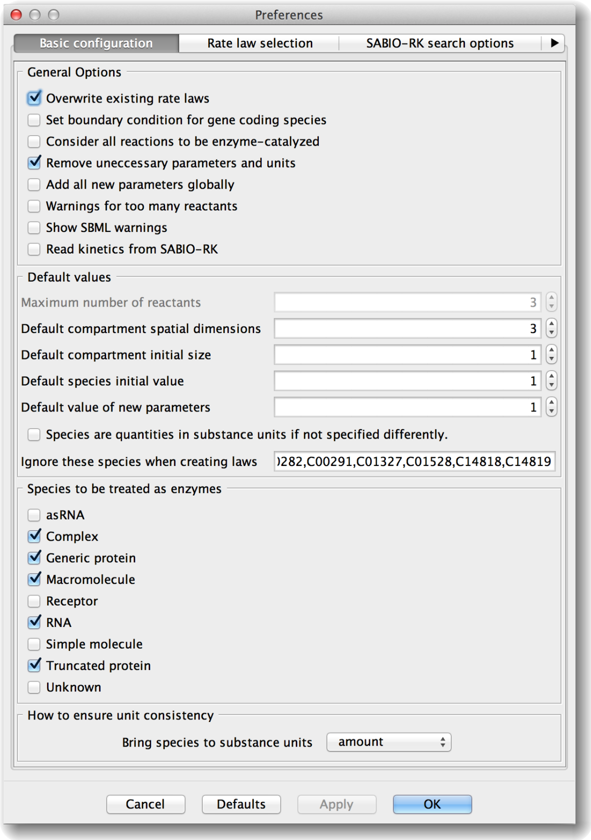

Figure 3.1: Basic configuration. All preferences in this tab correspond to the command line options described in section 4.3.3. You can specify if already existing rate laws should be replaced with newly generated ones, if a SABIO-RK search should be performed, etc. Taking small molecules and ions into account may lead to complex kinetic equations. You can here give a list of KEGG identifiers for those species to be ignored when creating rate laws (see table 5.1). If values are missing in the model, this tab allows you to specify default values to be inserted. You can decide how SBMLsqueezer should ensure unit consistency, by either bringing all species to amount units, or to concentration units. For situations, in which the role of modifiers is unclear, you can here select types of species to be considered enzymes.

overview of tools. You do not have to execute any further software besides your web browser.

3.2 Adjusting the preferences

The program SBMLsqueezer offers a large number of options to configure how kinetic equations are generated. All options described in this section can also be applied using SBMLsqueezer's command-line interface (see section 4.3 on page 40 for details). This means that you can already upon launching the program define with which options it should be initialized. Your configuration can always be set back to the default options if desired. SBMLsqueezer stores its configuration in the system's preferences of your operating system. This means that when launching the program for the next time, you can directly continue with your previous configuration. Furthermore, there are no configuration files generated by SBMLsqueezer in addition to those maintained by your operating system. Hence, SBMLsqueezer integrates well into your working environment.

We will now take a closer look at the options and describe what you can influence and which choices the program offers to you. In order to configure your preferences, you can click on the tool icon in the tool-bar (see the icon next to this text), or select the corresponding entry in the

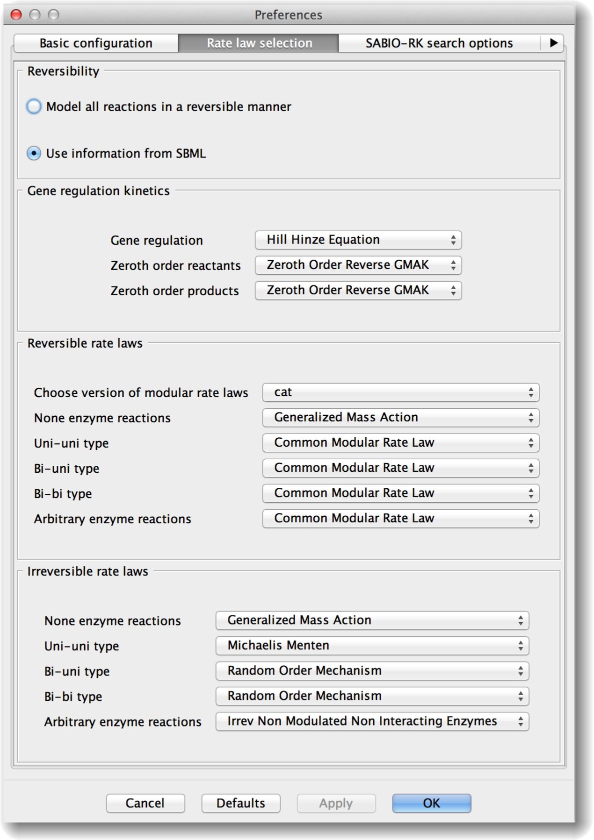

Figure 3.2: Rate law selection. It has been observed that irreversible rate laws are often too restrictive and cannot capture the dynamics of complex reaction systems. For this reason, SBMLsqueezer can create all kinetic equations in a reversible manner and will then also update the property *reversible* in affected reactions. Depending on this setting, you will be able to select default types of irreversible rate laws. The other sections of this tab allow you to select which type of rate law should be primarily selected when generating equations for an entire network in one single step. If you create equations for individual reactions only, as shown in fig. 3.2, these settings do not have an effect. For more information about your choices in this tab, see also the description of its corresponding command-line options in section 4.3.4 on page 45.

menu bar. Under Microsoft Windows and Linux, you can access the preferences menu under Edit › Preferences, whereas under Mac OS X, these can be found under SBMLsqueezer › Preferences... or by using the keystroke combination ⌘ + ,.

On the bottom panel of the preferences dialog you can see the four buttons Cancel, Defaults, Apply, and OK as this is displayed in figs. 3.1 to 3.5 on pages 11–15. The Cancel button has the same effect as a click on the close button on the top of the window or hitting the Esc key: all of your changes will be disregarded and the window will be closed. The Defaults button restores the standard settings of SBMLsqueezer. With Apply you can persistently save your current settings. The OK button is a combination of Apply and close, i.e., it makes your settings persistent and closes the window. Hitting the ⏎ key has the same effect as clicking on the OK button. Note that the dialog is structured with multiple tabs. When clicking on Defaults the preferences in all tabs will be restored, even if those are not active at the time when you hit that button.

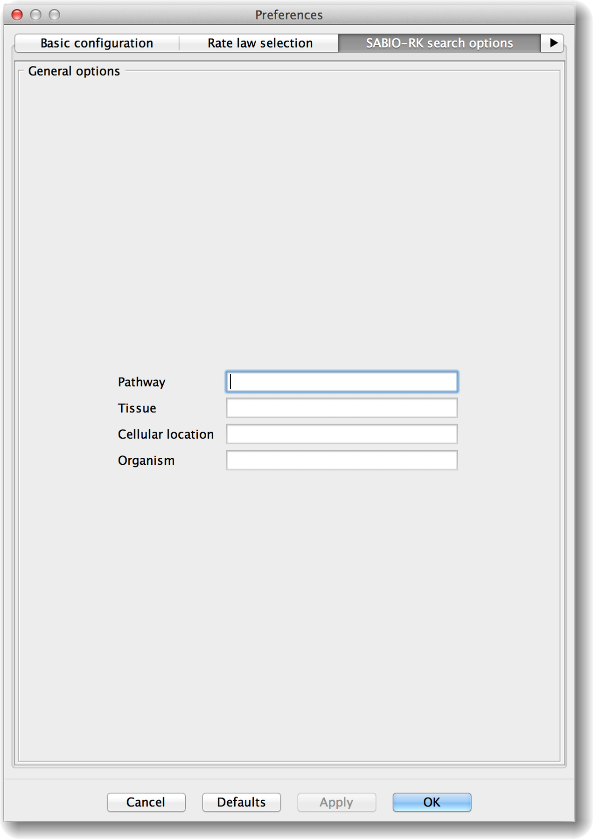

Figure 3.3: SABIO-RK search options. The options in this tab correspond to the command-line options that are described in section 4.3.5 on page 52. You can specify general features of the reaction for which you want to look up appropriate rate laws in SABIO-RK, such as the pathway, in which the reaction occurs, the tissue, in which it was observed, even the cellular location. Of course, you can also restrict your search to an organism of choice.

3.3 Open a model

The simplest way to open an SBML file is to click on File ⟩ Open in the menu bar, or at the folder icon (see the icon next to this text) in the tool bar. If you prefer keystroke combinations, you can use ⌘ + O on Mac OS X or Ctrl + O on Microsoft Windows and Linux computers. The GUI can open multiple SBML files in parallel. SBMLsqueezer arranges these SBML documents in tabs, whose order can be changed by dragging and dropping the tabs. Furthermore, you can also open one ore multiple models in SBMLsqueezer via drag and drop, i.e., just select one or multiple SBML files in an arbitrary file browser of your operating system and drag these into SBMLsqueezer's GUI.

When starting SBMLsqueezer from the command line, you can directly pass a model file to it, which will then be opened in the GUI. To this end, use the option `--sbml-in-file=<File>`, where `<File>` is the absolute or relative path to the SBML file you want to open (see section 4.3.2 on page 41). Note, however, that the command line option allows you to specify just one file to open. If you like to open further files, you need to do this in the GUI.

In case that you use libSBML as SBML back-end, SBMLsqueezer will conduct a model valida-

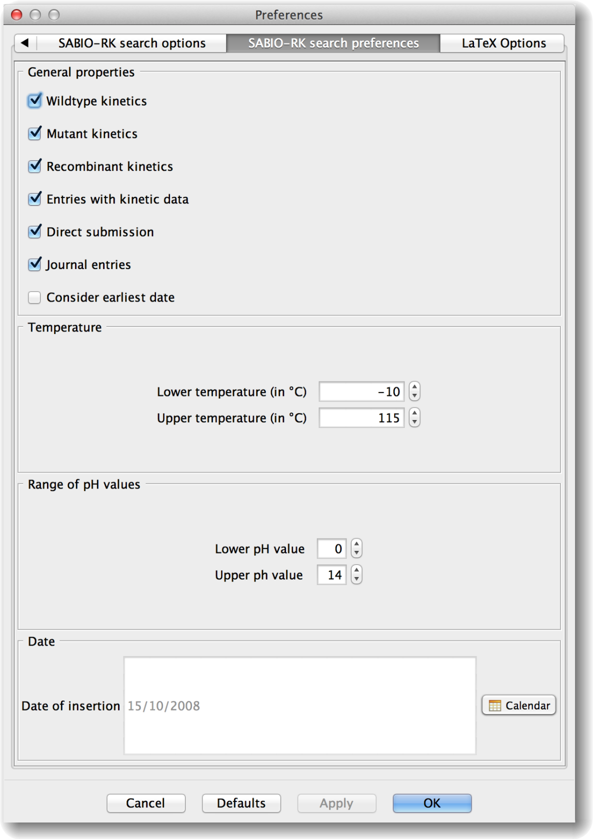

Figure 3.4: SABIO-RK search preferences. All preferences in this tab can also be changed through the command line. See section 4.3.6 on page 52 for details. Here you can define more fine-grained search search properties for looking up kinetic equations in SABIO-RK, such as the minimal and maximal temperature and pH value, under which the reaction proceeds, the currentness of the entry in SABIO-RK, and several boolean switches that influence which type of database entry can be considered. These features are mainly important when extracting rate laws for all reactions in the network in a bulk. When you search for kinetics of individual reactions, you may want to also apply more appropriate settings for just this reaction.

tion upon opening the file. The result of the validation will then be displayed in a dialog window. This feature is not available when using JSBML as SBML back-end, because the JSBML library does currently not implement an off-line validation for SBML documents.

SBMLsqueezer remembers up to ten files you have already worked with. Once you have opened one or more models, these are accessible in the menu bar, where you can click on File › Recent files. This sub-menu will display the names of the files and their absolute path as tool-tip. You can open one of these files by just clicking on its entry in the menu, or you can apply one of the keystroke combinations ⌥+0 to ⌥+9 on Mac OS X or Alt+0 to Alt+9 on Linux and Microsoft Windows. Here, the number keys are used to sort the previously opened models according to when you last accessed the file. The most recently opened file will always have number 0 and the model number 9 is the model that was opened a longer time ago. Note that this order will change as soon as you open one model from this list or any other model.

If you are working with CellDesigner, open the model with this program's menu item or keystroke combination. When launching the SBMLsqueezer as CellDesigner plug-in, it will always use the currently selected model. This means that, in case that you are working with multiple models in CellDesigner, this model will be passed to SBMLsqueezer, whose tab is currently selected.

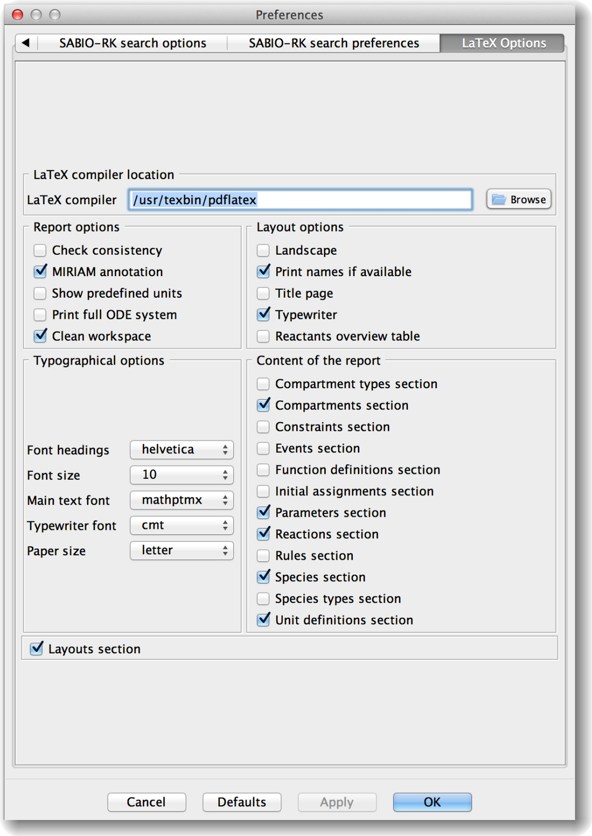

Figure 3.5: LaTeX options. All options in this tab are also available as command-line options (see section 4.3.8 on page 54). You can specify where your LaTeX compiler is located in your operating system and what to include into the model report. This tab also gives you several style options that influence how the the generated document will look like. As a new feature, it now also supports the Layout extension for SBML (Gauges *et al.*, 2006). You can here decide if a figure of your network should be generated and included into the report, given that layout information is provided in your file. For more information about SBML2LaTeX see the documentation of this project.

In Garuda, SBMLsqueezer can receive files from other gadgets and will open these whenever this is requested. SBMLsqueezer will automatically open accepted files. There is hence nothing further to do. If SBMLsqueezer is not running when a file is sent to it from another gadget, Garuda will automatically launch the program and the file will be opened upon start.

3.4 Equation generation one by one

You can add a kinetic equation to individual reactions. To this end, SBMLsqueezer provides a specialized context menu and dialog window, which enable you to select the type of rate law that seems appropriate for your purposes. This dialog window is available whenever using SBMLsqueezer as a plug-in for CellDesigner or in the stand-alone version (also in the Garuda version). In all cases, the same dialog window will appear, which offers you a selection of appropriate rate laws for the particular reaction and gives you the opportunity to choose between those, or to discard the dialog. No change will be performed to your model when canceling the dialog (either by pushing Esc or by clicking on Cancel).

We here use model № 390 from BioModels database (Li *et al.*, 2010; Arnold and Nikoloski,

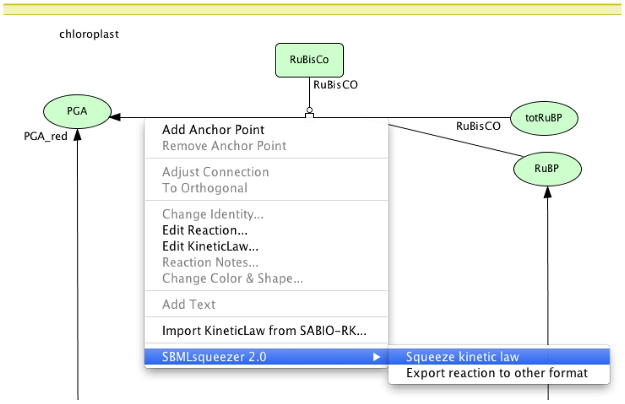

Figure 3.6: Reaction context menu in CellDesigner. After installing SBMLsqueezer as a plug-in, its functionality will be incorporated into CellDesigner's reaction context menu. If you right-click on an arbitrary reaction, it allows you to create a kinetic equation for this reaction (with the dialog in fig. 3.7), or to generate a LaTeX model report that only involves the details relevant for the selected reaction.

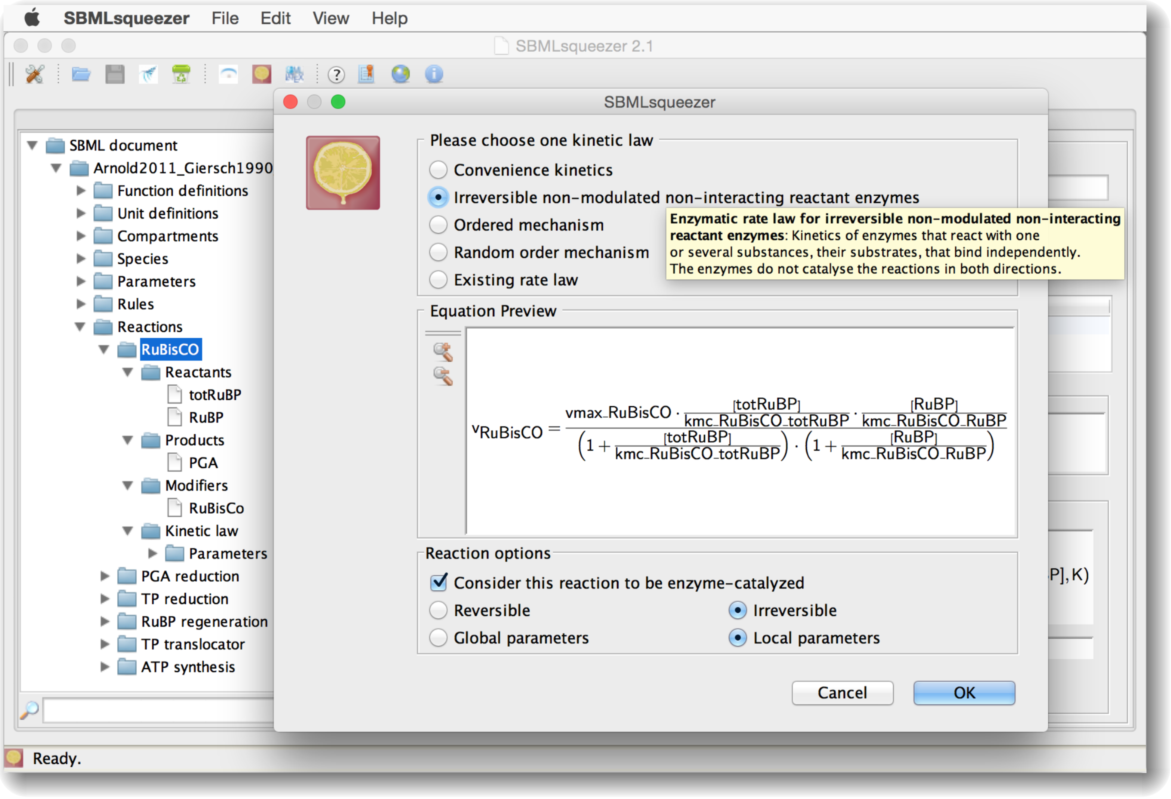

Figure 3.7: Generating a kinetic equation for a single reaction. This dialog can be opened by right-clicking on an individual reaction in your active model and selecting Squeeze in the pop-up menu. The directionality of the reaction and the enzyme property can be altered. Both options possibly influence the selection of rate laws. To abort this dialog, click on Cancel or hit the Esc key.

2011) as an illustrative example.

In case that you are working with CellDesigner (Funahashi *et al.*, 2003, 2006, 2007, 2008), right-click on the reaction of interest as this is displayed in fig. 3.6 on page 16 and select the menu item Squeeze kinetic law. When using SBMLsqueezer as a stand-alone program, you can generate kinetic equations for a selected reaction by right-clicking on the reaction of interest. An example is given in fig. 3.7 on page 16. SBMLsqueezer analyzes the currently selected reaction according to various properties and displays a selection of suitable rate laws, of which you can select the most appropriate one.

The equation preview, where you can zoom in and out of the rendered formula, helps you to choose a rate law. If a corresponding SBO entry can be found for a rate law, a tool-tip will be displayed with further information about this equation. You can also decide if the reversibility of the reaction should be changed. This would also alter the list of applicable kinetic equations. The other options, if newly created parameters should be locally attached to the kinetic law object or globally to the model, influences the internal structure of the model, but not its behavior. Just note, that there are some parameters, which cannot be stored locally because they represent recurrent properties of some reactive species and are needed across several rate laws (e.g., energy constants).

3.5 Generate kinetic equations in a single step

SBMLsqueezer can create rate laws for all reactions in one single step without the need of further human interaction or manual selection of rate laws for any reaction. This option can be useful if you are working with a large model and want to create a first draft of a kinetic system, or if you are interested to apply the same type of approximative rate law to each reaction. To see how to use this program feature, have a look at fig. 3.9 on the next page.

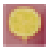

Just like in the case of the reaction context menu (section 3.4 on page 15), the same kinetics wizard can be used in stand-alone mode and when working with the CellDesigner plug-in version. Various options allow you to customize how this is done. The most important options decide if already existing rate laws should either be kept or overwritten, if all reactions can be modeled in a reversible manner, or as currently defined in the model. In the CellDesigner plug-in version, just click on Plugin > SBMLsqueezer 2.1 > Squeeze kinetic laws to launch the kinetics wizard. In the stand-alone version, you can either right-click on the model in the SBML tree, the lemon icon in the tool bar (icon next to this text), or Edit > Squeeze in the menu bar.

The usage of the kinetics wizard is identical, irrespective of how you launched it. You can see how this wizard looks like in fig. 3.9 on the next page. You can always discard all your changes by either clicking on the Cancel button or by hitting the Esc key. The Help button opens a browser window that gives you more information about SBMLsqueezer. If you click on Show options, the preference dialog will be displayed, which is described in section 3.2 on page 11. In this way, SBMLsqueezer gives you the option to make sure that the selection of kinetic equations for all distinguished cases will be done as if you pick those individually.

When you hit the Next button, kinetic equations will be created according to your settings and

Figure 3.8: Launching SBMLsqueezer in CellDesigner. The menu item Plugin ⟩ SBMLsqueezer 2.1 gives you access to nearly all functions of the program. You can launch the kinetics wizard, adjust your preferences, export a LaTeX report, open a help browser, or just look at how SBMLsqueezer internally represents CellDesigner's data structure.

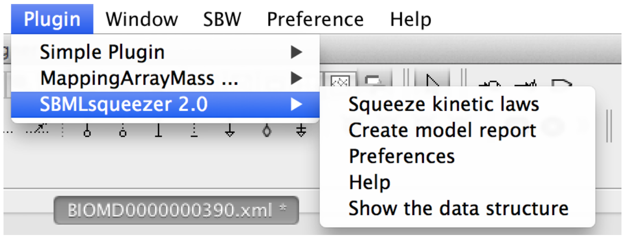

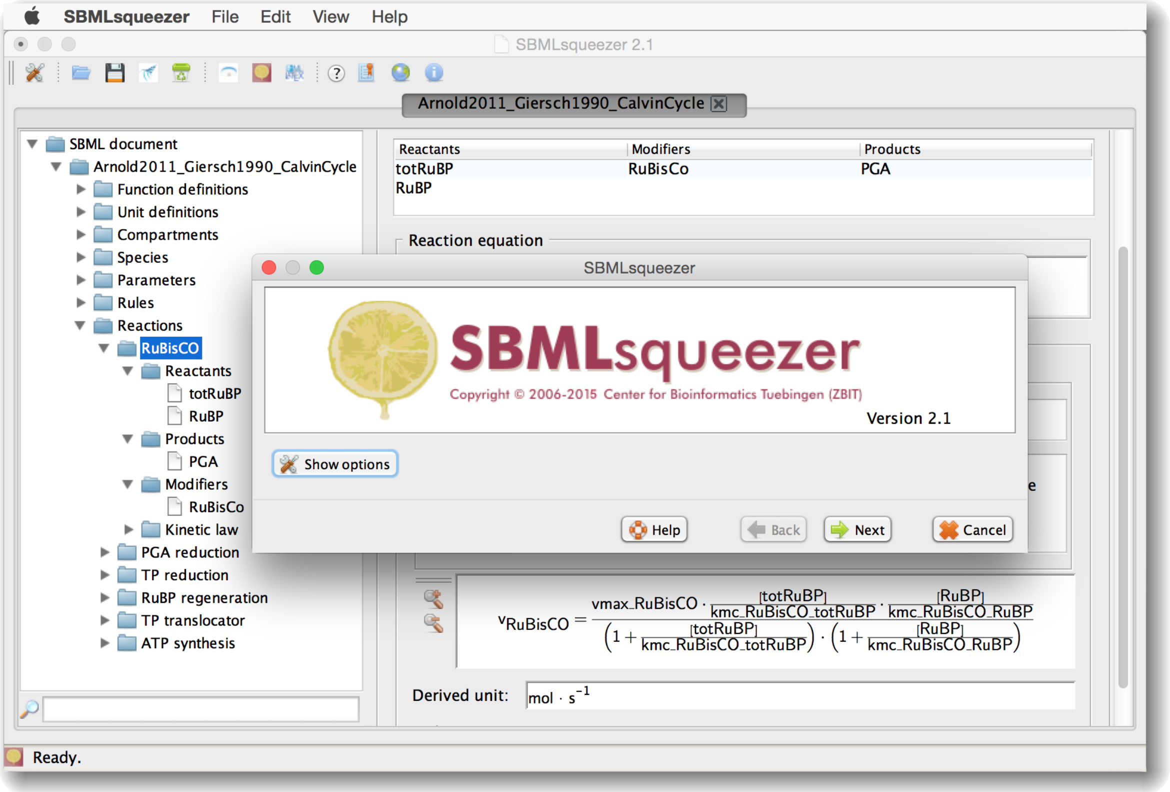

Figure 3.9: Generating kinetic equations in one single step with the kinetic law wizard. This screen-shot shows the Kinetic Law Wizard (here shown under Mac OS X). When launched, this wizard allows you to generate kinetic equations for all reactions in the active model. From this wizard, you can directly access and alter all program options, see the online help, and go back and forth between all steps through which the wizard will guide you.

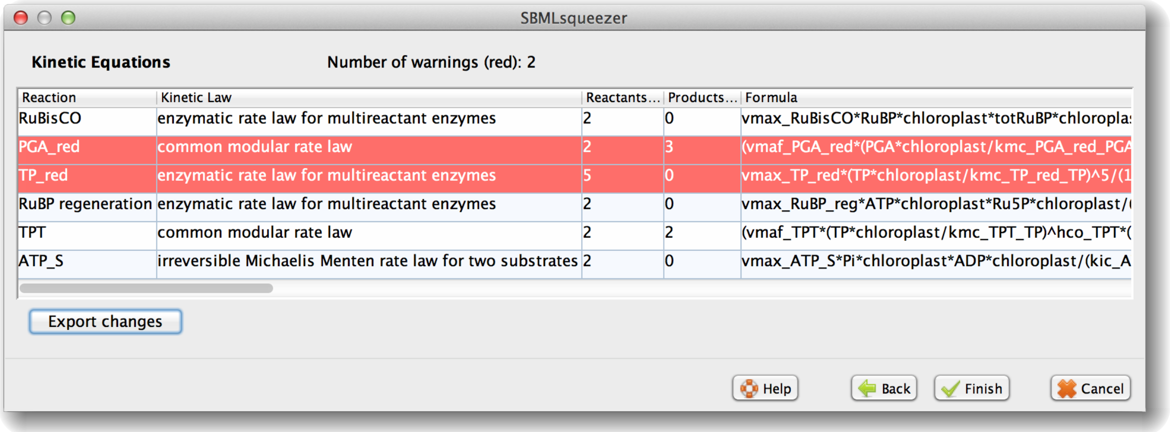

Figure 3.10: Results of the kinetics wizard displayed in a table. We again use model № 390 from BioModels database (Li *et al.*, 2010; Arnold and Nikoloski, 2011) as an example. Two rows are highlighted in red. The reason is that these reactions involve an unrealistically high number of reactant molecules, or are reversible with a high number of products, respectively. These warnings indicate that the generated rate law might neglect several intermediate steps. In this table, you can review all generated rate law. Note that these will not be transferred to your original model before you hit the Finish button. You can alter each rate law by double- or right-clicking on an entry in the column "Kinetic Law," or hit the Back button and change your preferences for the rate law selection. You can already generate a LaTeX report about the changed model (hit the button Export changes), which can help you to make your decision if you want to apply or discard your changes.

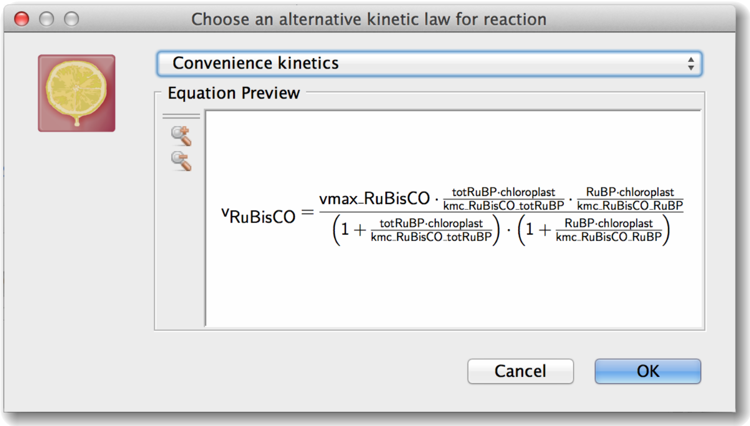

Figure 3.11: Changing selected rate laws in the kinetics wizard. This dialog window appears when you double-click on an entry in the column "Kinetic Law" of the kinetics wizard. You can here select and apply an alternative rate equation. If you want to change the criteria for the creation of equations, you need to go back to a previous step in the wizard.

Figure 3.12: Starting the SABIO-RK wizard. After opening a model in SBMLsqueezer, the SABIO-RK button will become active in the tool-bar (see the black fringe). A tool-tip will display more information about this button when moving the mouse over it. You can click on this button to launch the SABIO-RK wizard. Alternatively, you can also click on Edit › SABIO-RK.

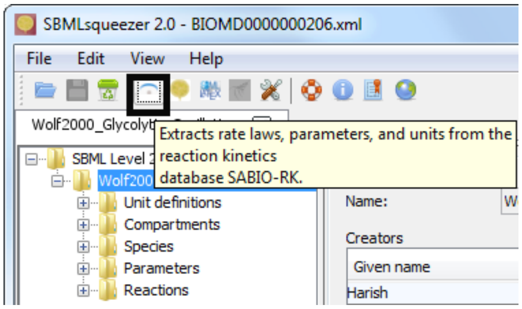

displayed in a summary table (see fig. 3.10 on page 19). You can change individual rate laws by double-clicking or right-clicking on a specific entry in this table. A pop-up window will then appear asking you to choose an alternative rate law (see fig. 3.11 on page 19). When applying the change, the selection will be updated. When you hit the button Finish, the created rate equations, units, and parameter objects will be incorporated into your original model.

3.6 Extraction of rate laws from SABIO-RK

Instead of deriving kinetic equations *de novo* for your model, you can also use SBMLsqueezer to automatically obtain experimentally determined equations from SABIO-RK. To this end, SBMLsqueezer compares the reactions of interest to reaction signatures in this online database and allows you to select the most appropriate reactions or to restrict your search by using several parameters, such as pH, temperature, etc. Note that this feature does not apply for the CellDesigner plug-in variant, because CellDesigner brings already with it its own SABIO-RK interface, which you can use directly in CellDesigner. Furthermore, this feature requires an active Internet connection and can therefore not be used in an off-line mode.

This section describes how to use the GUI to extract information from SABIO-RK. You can also do this on the command line, which allows for batch processing of multiple SBML files as this has been done by Büchel *et al.* (2013). For more information about the command-line options for SABIO-RK see section 4.3 on page 40. You can directly use SBMLsqueezer's API to benefit from this functionality. Section 4.1 provides an elaborated description of how to use this feature.

3.6.1 Rate laws from SABIO-RK for an entire network

Figures 3.12 to 3.17 show how you can add kinetic equations from SABIO-RK to several reactions at once. As an example model we pick model № 206 from BioModels database (Li *et al.*, 2010; Wolf *et al.*, 2000). A wizard guides you through the selection of relevant reactions (fig. 3.13 on the facing page) and provides several options to restrict the search in SABIO-RK (fig. 3.14 on the next

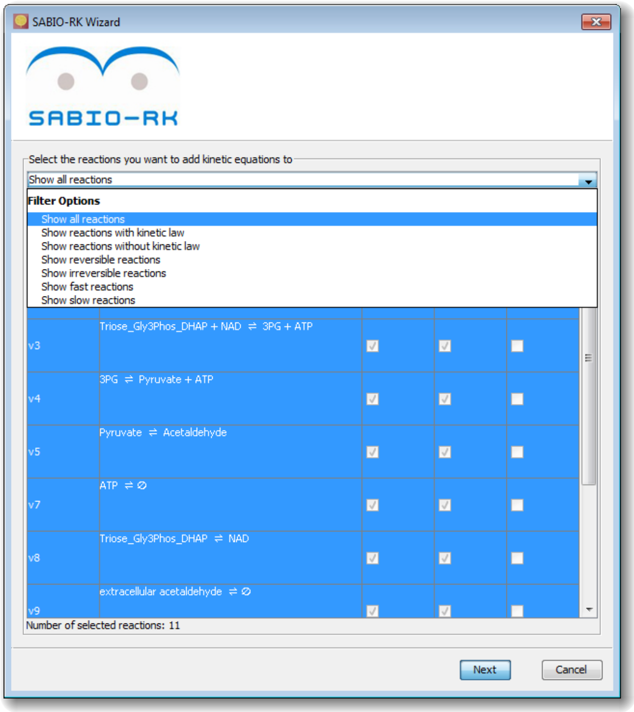

Figure 3.13: Selection of reactions in the SABIO-RK wizard. On the first window you can select which reactions you want to equip with kinetic equations from SABIO-RK. It is possible to directly select the reactions with some property (such as reactions not yet containing a kinetic law or fast reactions). Furthermore, you can select the desired reactions by holding the Ctrl key pressed while clicking on the desired reactions. You can abort the wizard by either clicking on the Cancel button or by hitting the Esc key. When finished click on the button Next.

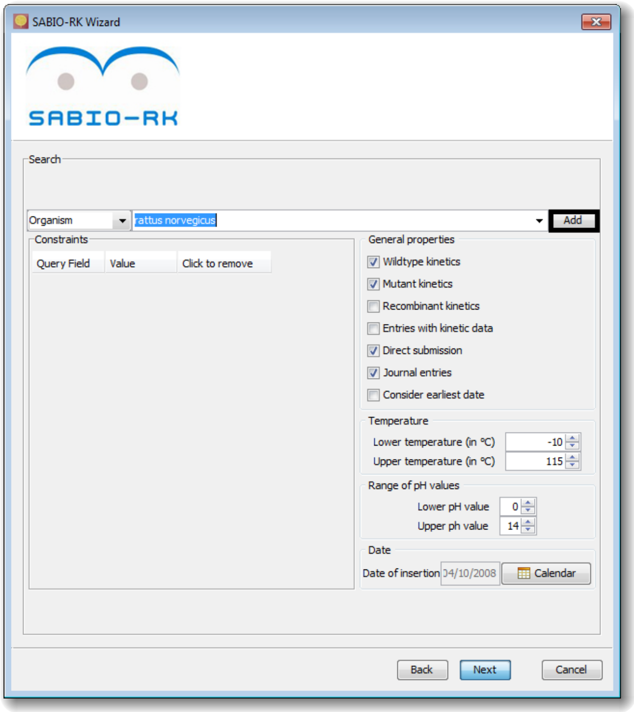

Figure 3.14: Selection of search terms for SABIO-RK. On the next window you can add categories for search terms together with their values. These terms are used to select only reactions and rate laws from SABIO-RK, whose annotation contains these terms. An example is the organism, in which this reaction takes place. You can choose "organism" from the provided terms and type the desired name as value of the term in the text field. By clicking on button Add (black fringe), the term will be added to the table on the left-hand side of the dialog window. See fig. 3.15 on the next page for an example how to remove a term again.

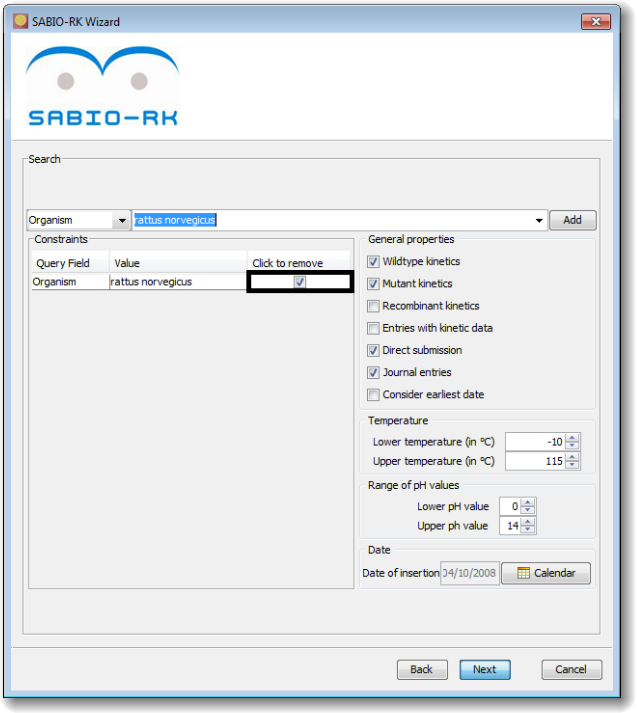

Figure 3.15: Removal of search terms for SABIO-RK. You can remove a query field again by clicking on the check box button in the right-most column. When you are done with selecting appropriate search terms, click on button Next to see the results of your query. Figure 3.16 displays an example who this overview can look like. The Back button allows you to return to the selection of reactions as displayed in fig. 3.13 on page 21. As before, you can abort the dialog by either hitting the key Esc or by clicking on the Cancel button.

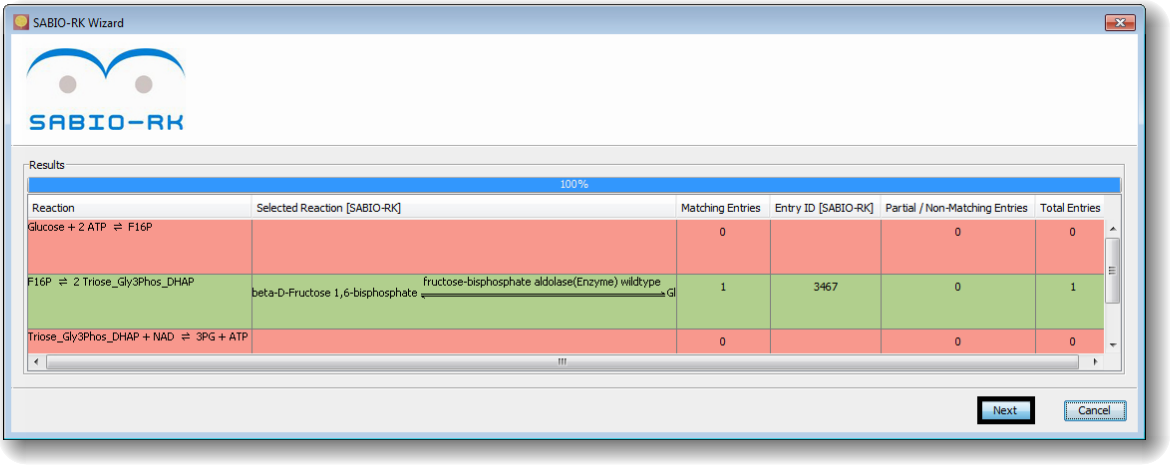

Figure 3.16: Window with found kinetic equations. A reaction is highlighted in green if a matching rate equation was found, yellow if the found equations do not exactly fit to your model (there is no straightforward matching of all elements in the equation to elements in your model), and red if no matching rate equation was found. If you are satisfied with the results, click Next.

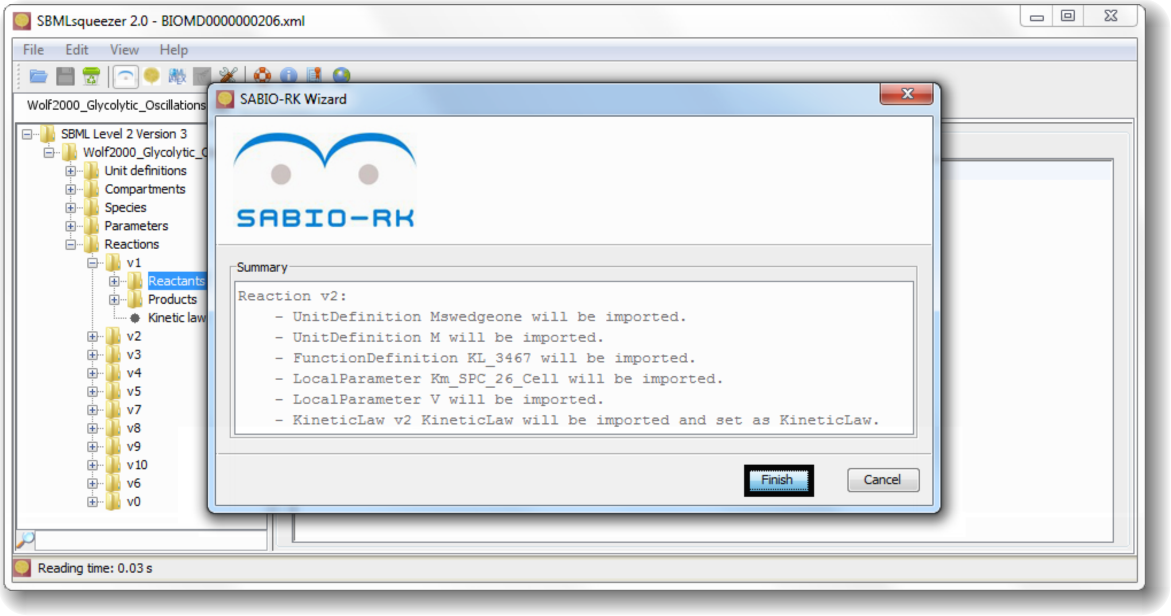

Figure 3.17: Summary of necessary changes. The wizard summarizes all changes to the model that are required if you want to transfer the identified rate laws from SABIO-RK to your model. This summary lists only changes related to reactions that have been highlighted in green in the previous step (fig. 3.16 on page 22). Upon clicking on Finish a pop-up dialog will finally ask you to either confirm these changes to be made or to disregard all results. If you confirm, the rate equations will be transferred to your model and all changes will be applied as stated in this summary.

page), whose interface is similar to the database front end of SABIO-RK[1]. To ensure that entries for the reactions of interest can be found in SABIO-RK, it is important to annotate these reactions with IDs from the Kyoto Encyclopedia of Genes and Genomes (KEGG) database (Kanehisa and Goto, 2000) using the MIRIAM registry (Juty *et al.*, 2012). The KEGG reaction ID is then always included in the respective search in order to avoid adding kinetic laws referring to other reactions. To further restrict your search, change the bounds of pH, temperature, etc. in the right panel (see fig. 3.14 on page 21).

3.6.2 SABIO-RK for a selected reaction

As an alternative you can add kinetic equations from SABIO-RK to a selected individual reaction. How to do this is shown in figs. 3.18 to 3.23 on pages 24–27. We here use model № 206 from BioModels database (Li *et al.*, 2010; Wolf *et al.*, 2000) as an example network. You should first select the reaction, for which you want to obtain a rate law from SABIO-RK (fig. 3.18 on the next

[1] `http://sabio.h-its.org`

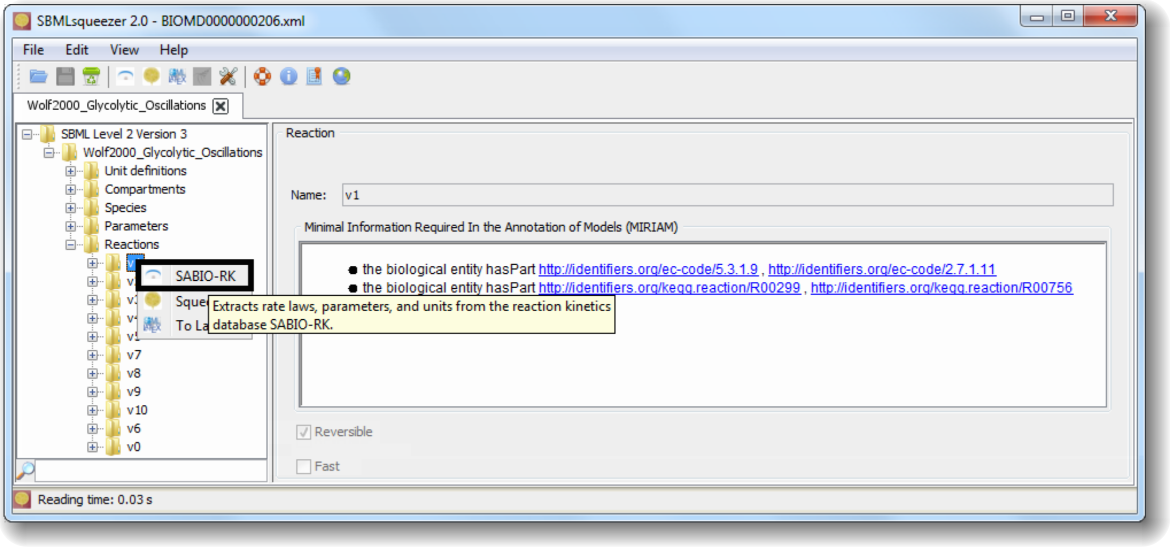

Figure 3.18: Starting the SABIO-RK application for a selected reaction. To find a reaction of interest, you can use the search function at the bottom of the SBML tree. Just click on the text field and type a part of the reaction's name or ID. The tree will be updated while you type and reduced to elements that contain the text you are typing. Alternatively, you can just scroll to the entry of interest. Once you have found a reaction of interest, right-click on it. Then click on the SABIO-RK item in the pop-up menu and the SABIO-RK wizard will be started. The reaction is shown again in the first window and you can just click on Next. This will bring you to the selection of search terms that is described in fig. 3.19 on the facing page.

page). All subsequent steps of the procedure are essentially the same as described before. The only difference is that the SABIO-RK wizard will be launched for one selected reaction of interest only instead of having all reactions in the model.

3.7 Viewing and saving the results

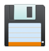

When using the stand-alone version of SBMLsqueezer, you can see a tree representation of the SBML document you are working with on the left-hand side of the GUI. Figure 3.24 displays how model № 390 from BioModels database (Li *et al.*, 2010) looks like after SBMLsqueezer has modified one reaction's rate law. The program recognizes if an SBML document has been modified by one of its function. You can see this because the title bar of the program will change, and the save actions in tool-bar and menu-bar will become active. In Microsoft Windows and Linux an asterisk will be added to the file name in the title bar. In Mac OS X, the red "close" button on the top-left most corner of the program will no longer display a cross, but a circle (you can see this in fig. 3.24 on page 28). You can save the modified file either by clicking

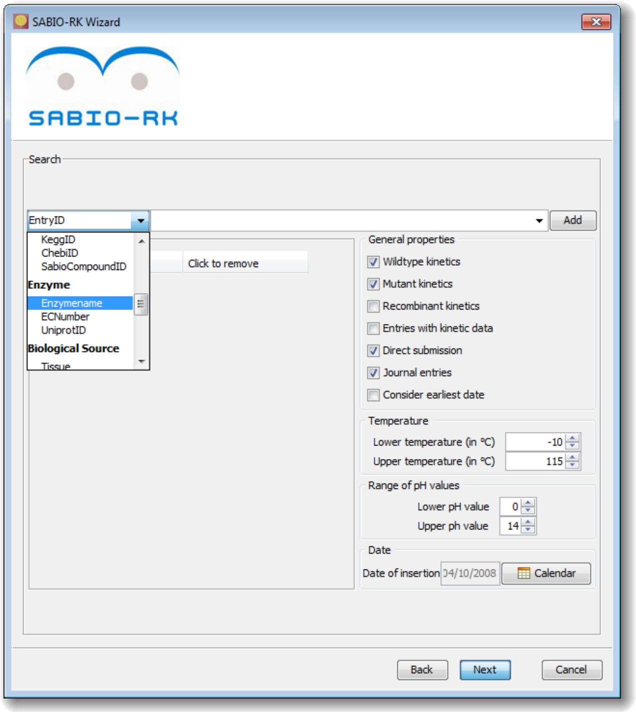

Figure 3.19: Selection of search terms. You can add search terms such as the organism by choosing from the provided terms and typing in the value of the term. The search can be further restricted by changing the bounds of pH, temperature, etc. in the right panel. You can remove a query field by clicking on the check-box button in the table on the left-hand side. This dialog is just the same what has been described in figs. 3.14 to 3.15 on pages 21–22 when adding kinetic laws to several reactions in a single step. The example continues in fig. 3.20.

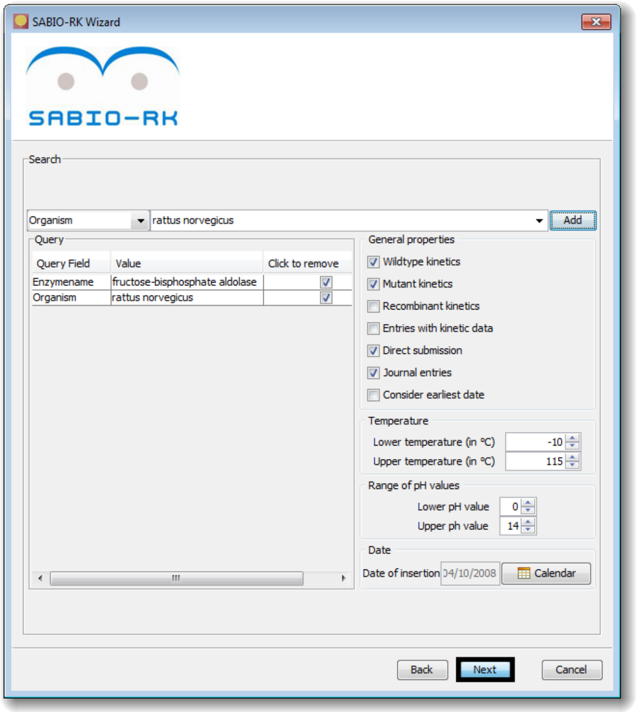

Figure 3.20: Window with two search terms. Here a search for an organism and an enzyme name has been specified and will be combined to a query when clicking on Next. You will then see the list of reactions as it is displayed in fig. 3.21 on the following page.

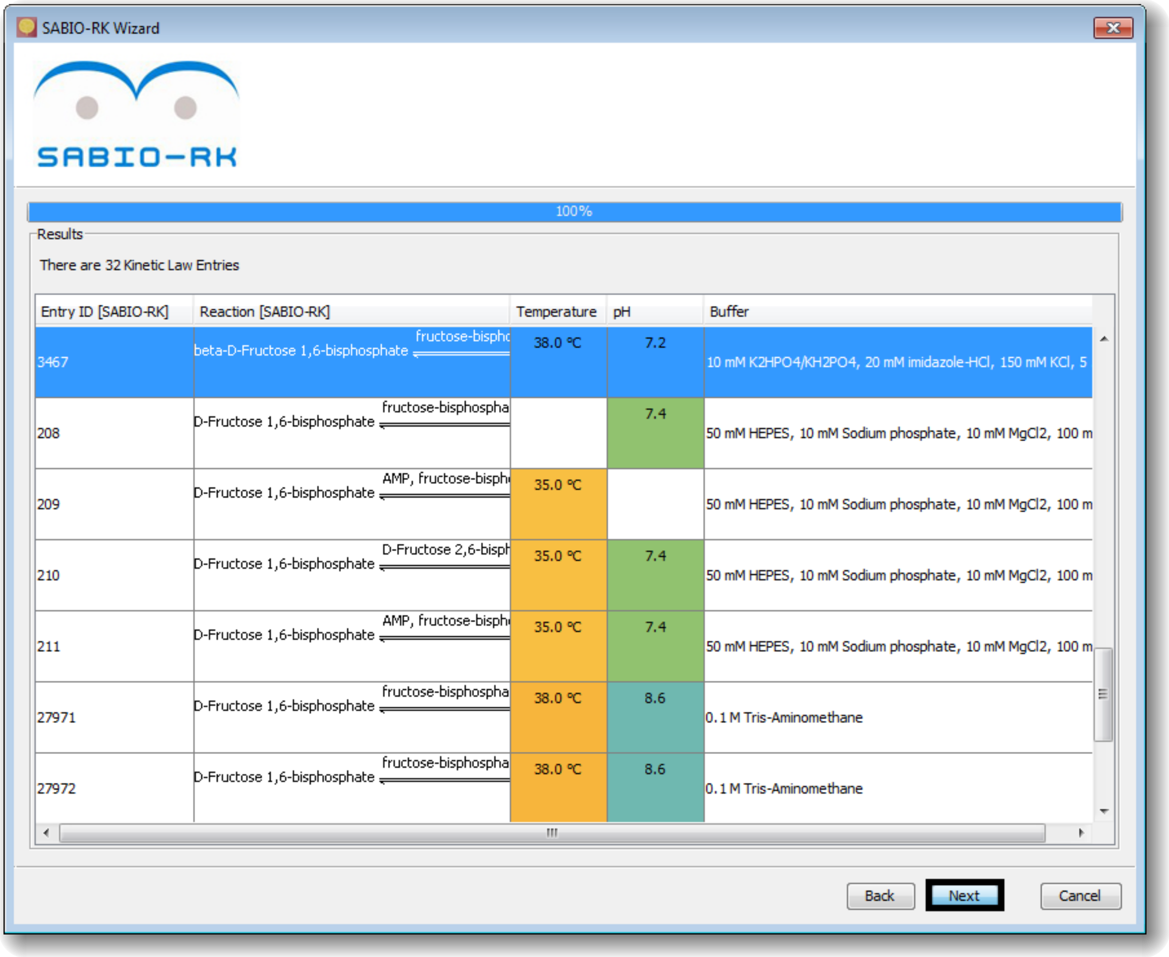

Figure 3.21: Window with kinetic equations found for a single reaction. The rate equations that match your search criteria are displayed here. You can select a reaction and click on Next, which will bring you to fig. 3.22 on the facing page. Alternatively, you can click on Back and change your search criteria (see fig. 3.20 on page 25).

on File › Save or by clicking on the disk symbol in the tool-bar. Alternatively, you can also use the keystroke combination Ctrl + S (under Microsoft Windows and Linux) or ⌘ + S (under Mac OS X).

You can always select File › Save as... in order to save the active SBML document under a different file name. The keystroke combination ⇧ + Ctrl + S (Microsoft Windows and Linux) ⇧ + ⌘ + S (Mac OS X) will also open a file chooser to save a copy of the active SBML document. In Mac OS X you have a further options to save the currently selected document elsewhere: you can drag the file symbol in the title bar into an arbitrary folder of a file browser.

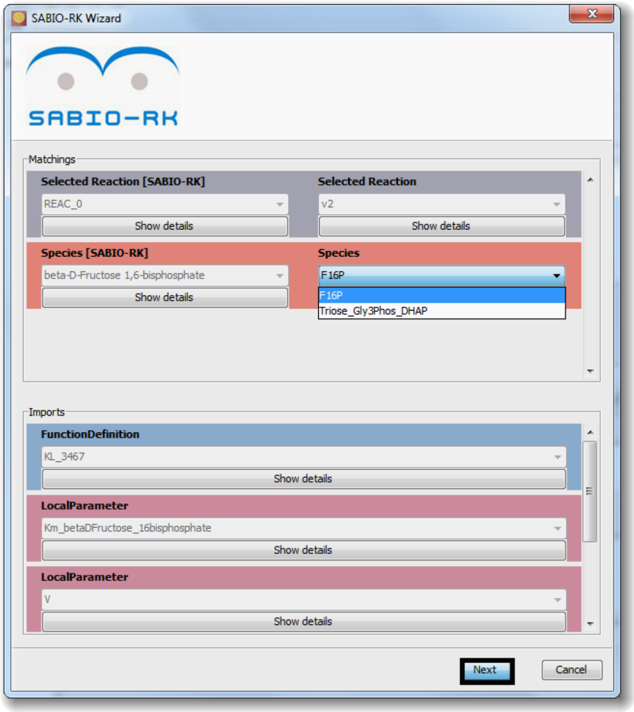

Figure 3.22: Window for matching elements in the equation to model elements. If kinetic laws are added to an entire network simultaneously, a straightforward matching of all elements (e.g., the species) contained in each rate equation to SBML elements in your model will be necessary. In contrast to that, if you only add a rate law to one reaction at a time, the application presents you a suggestion for the matching and enables you to change it if desired. The necessary elements to import, such as function definitions contained in the rate equation to add, are also shown in this window.

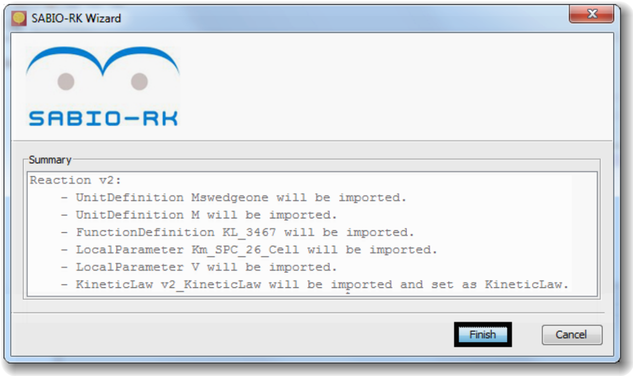

Figure 3.23: Summary of necessary changes. This overview summarizes how your model will be changed when adding the selected rate equation. Now click on Finish and afterwards a dialog pops up asking you to confirm the changes. After this confirmation the rate equation will be transferred and these changes will be applied to your model.

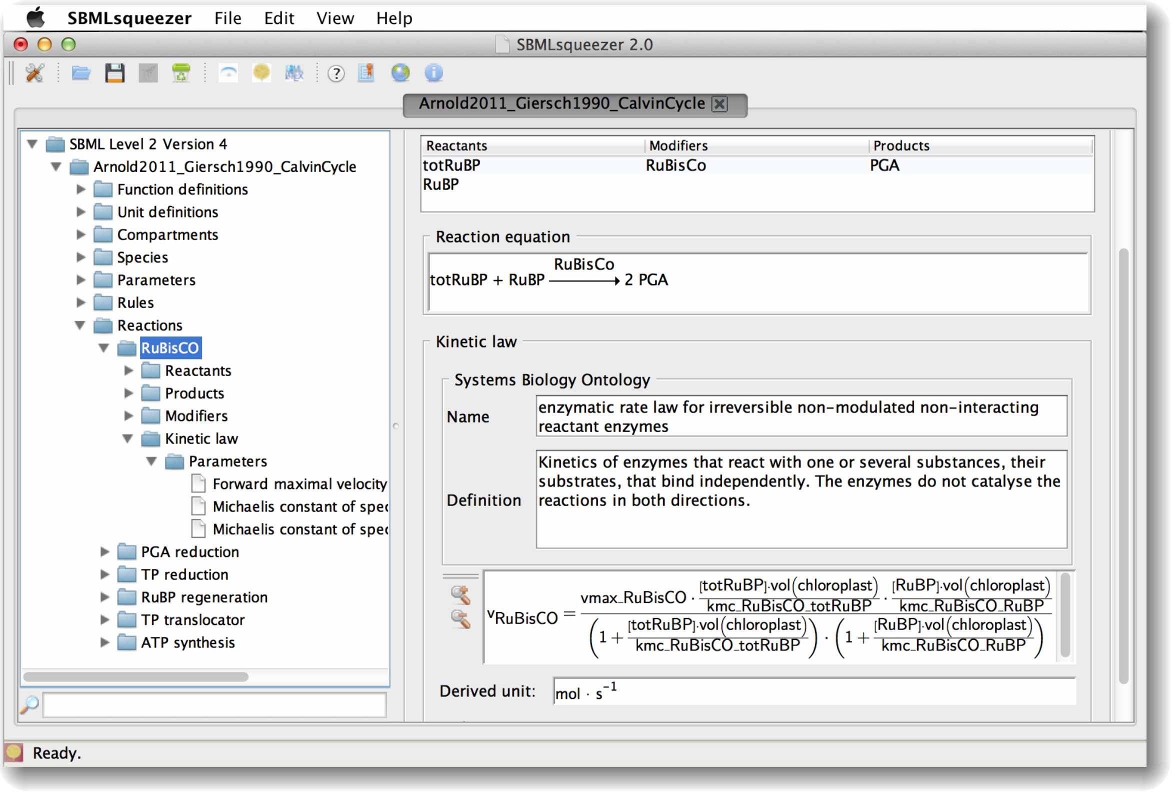

Figure 3.24: Viewing SBMLsqueezer's results. SBMLsqueezer provides a two-part view that displays the details of the model's structure. On the left you can see the hierarchical SBML data structure. By clicking on selected elements within this tree, details about the element will be displayed on the right. If you select a reaction or a kinetic law directly, the rate equation, parameters, etc. will be displayed. You can search for elements within the model by using the text field on the left-bottom corner. This text field reacts to your input while typing and will immediately reduce the number of selectable items in the tree. Exact hits will be highlighted in a bold font.

Now, let us assume, you have used SBMLsqueezer to assign kinetic equations to the reactions in your model (either by extracting those from SABIO-RK or with the *de novo* equation generator). When expanding that tree and clicking on individual components, the program enables you to view the generated kinetic equations. Just expand "Reactions" and afterwards the desired reaction. Then you can click on "Kinetic law" and the rate equation is displayed on the right in detail. A convenient search function at the bottom of the window helps you to quickly find reactions and other model components of interest.

When using SBMLsqueezer as a plug-in for CellDesigner, you can either see the results by looking through the tables and overviews provided by CellDesigner itself, or you can click on Plugin 〉 SBMLsqueezer 2.1 〉 Show the data structure. This will open a similar view as the stand-alone

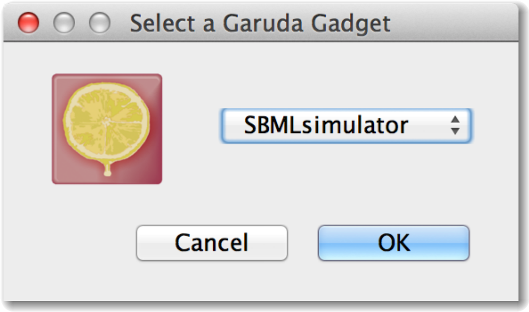

Figure 3.25: Selecting a Garuda gadget. This dialog window shows you all available Garuda gadgets that support the file type you are sending. Upon hitting the OK button, the model in the currently selected tab will be sent to the selected application.

version provides. SBMLsqueezer automatically synchronizes all of its changes with CellDesigner. This means that CellDesigner will be notified about these changes and incorporate these into its in-memory model. You can hence directly use its save functions or also save a copy of your model.

3.8 Interaction with further Garuda gadgets

After having kinetic equations assigned to reactions of interest, it might be useful to pass the resulting SBML document to further gadgets in Garuda for subsequent analysis. SBMLsqueezer is automatically connected to Garuda core if it has been launched inside of Garuda. Otherwise, SBMLsqueezer will also connect to Garuda if you launch your installation of Garuda before starting SBMLsqueezer. If SBMLsqueezer is already running when you launch Garuda, no new connection will be established, you would need to restart SBMLsqueezer.

Now let us assume, your connection to Garuda has been successfully established. In this case, SBMLsqueezer can both, receive and send SBML files from or to further gadgets. SBMLsqueezer will notify you when it is connected to Garuda by displaying a message in its status bar (at the bottom of the main window). You do not have to do anything in SBMLsqueezer when you want to receive a file from another gadget. The program will automatically be notified as soon as it receives the file and will directly open it in a new tab, so that you can work with the file as if you have just regularly opened it within the application.

It might, for instance, be an interesting idea to create a draft for a dynamic model directly based on information from the KEGG database (Kanehisa and Goto, 2000) using KEGGtranslator (Wrzodek *et al.*, 2011, 2013) and to pass the model to SBMLsqueezer in order to assign kinetic equations. This model can then be forwarded to the application SBMLsimulator (Keller *et al.*, 2013; Dörr *et al.*, 2014) that can be used to estimate the parameter values within the newly created kinetic laws, given that reference data are available. A similar approach has been suggested for the generation of draft genome-scale metabolic models (Büchel *et al.*, 2013).

As soon as at least one SBML file has been opened, SBMLsqueezer will activate the Garuda icon in the tool-bar (which you can see next to this text) as well as the menu item File ⟩ Send current file to Garuda. By clicking on that icon or selecting this entry in the menu bar, you can send the active file, i.e., the file that belongs to the currently selected tab in

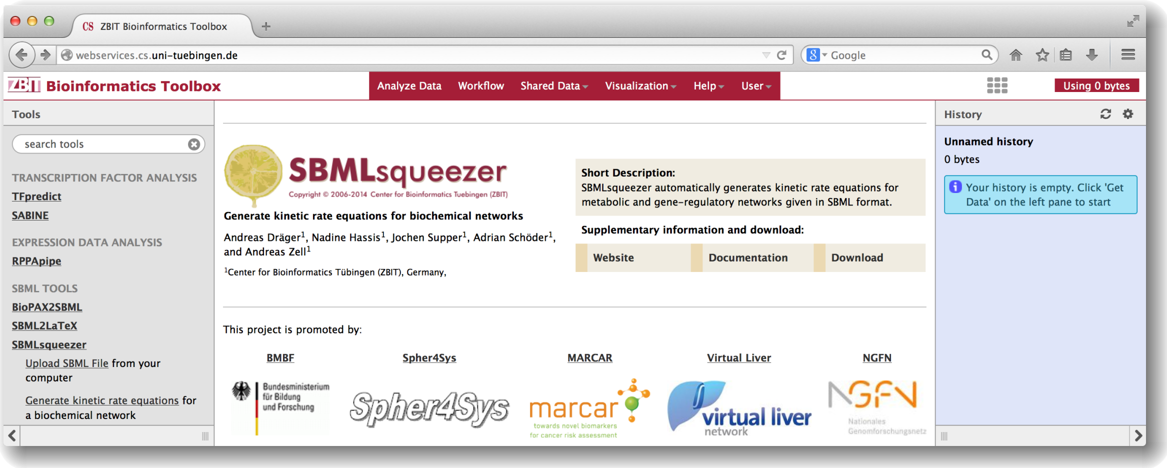

Figure 3.26: Starting the online version of SBMLsqueezer. This figure displays the Galaxy framework in the browser Firefox (version 29.0.1) under Mac OS X (version 10.9.3). You can start working with SBMLsqueezer by either clicking on the program's icon or the link "Upload SBML file".

SBMLsqueezer, to another gadget. To this end, SBMLsqueezer will display the dialog window that is shown in fig. 3.25 on page 29. If the model in the selected tab has been modified and not yet saved, SBMLsqueezer will create a temporary file in your operating system's directory for temporary files and send this file to the selected gadget.

3.9 Using SBMLsqueezer as an online program

SBMLsqueezer can also be used via the Galaxy web service facility (Goecks *et al.*, 2010) of the University of Tuebingen[2]. Since the user interface of this variant greatly differs from all other use-case scenarios, we here give a description of options and functions of the web interface.

In order to use the online version of SBMLsqueezer, open a browser of your choice. Go to the main portal of all web services provided by the Universtiy of Tuebingen[2] and scroll down until you find the tool SBMLsqueezer and click on the the program's logo, or you can click on the link in the list of tools on the left. Alternatively, you can directly go to the service SBMLsqueezer[3]. Figure 3.26 displays the web interface of the Galaxy framework (Goecks *et al.*, 2010).

The first step is now to upload your SBML file of interest to the online program. To this end, you can either click on the link "Upload SBML file" on the left-hand side of the browser window, or click on the icon of SBMLsqueezer. In both cases you should see a browse button that allows you to select an Extended Markup Language (XML) or SBML file from your local file system. After

[2] http://webservices.cs.uni-tuebingen.de
[3] http://webservices.cs.uni-tuebingen.de/tool_runner?tool_id=sbmlupload2

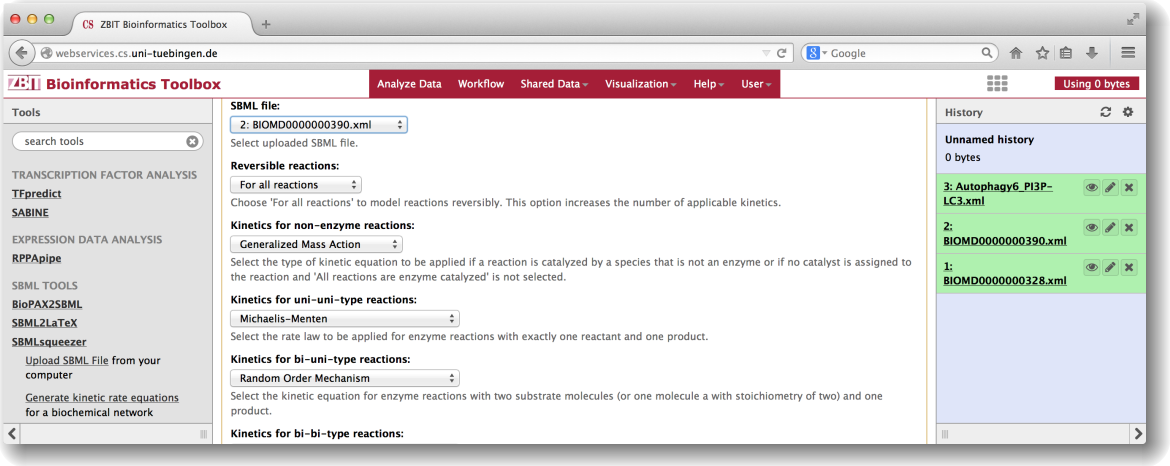

Figure 3.27: Adjusting preferences in the online version. In this example, three models have been uploaded to the Galaxy service and are shown in the history column in the right-hand side of the browser window. You can now choose for which file SBMLsqueezer should generate kinetic equations by altering the selected entry in the combo box at the top of the center column. In the center column, this view offers you all options of the stand-alone version of SBMLsqueezer.

selecting the file, hit the button “Execute” in order to actually upload the file to the Galaxy service. Galaxy informs you about the progress of the upload in the “History” column at the right-hand side of the browser window. You can upload further files if you like, but at least one file is required in order to run SBMLsqueezer. Please note that uploaded models and files will be deleted after the job has been executed and are not accessible to any third-parties, i.e., data security is guaranteed.

Second, click on the link “Generate kinetic rate equations” on the left-hand side of the browser window. You should see a view similar to what is displayed in fig. 3.27. Scroll down to see all available options. At the bottom of this page, there is also a descriptive text explaining how to proceed. Each option has descriptive text as well. The online version presents all options of the program just as the stand-alone version. You can therefore go back in this tutorial and look-up the meaning of individual options either in section 3.2 on page 11 about the GUI of the stand-alone version or in section 4.3 on page 40 where the command-line options are described. When you scroll down, you can see that this online program also offers you to obtain rate laws from SABIO-RK. Just one option group is missing, namely SBML2LaTeX. The reason is because the Galaxy framework of the University of Tuebingen provides SBML2LaTeX as a separate online program (Dräger *et al.*, 2009b).

When you are happy with the settings, scroll down until you see the “Execute” button. When you hit this button, SBMLsqueezer will create kinetic equations for your model according to your preferences. To this end, a new job will be scheduled that will be displayed in your history.

When the job is done, you can obtain the results by first clicking on the link “Generated kinetics for [file name],” which will appear in the history column. You can then click on the disk icon below

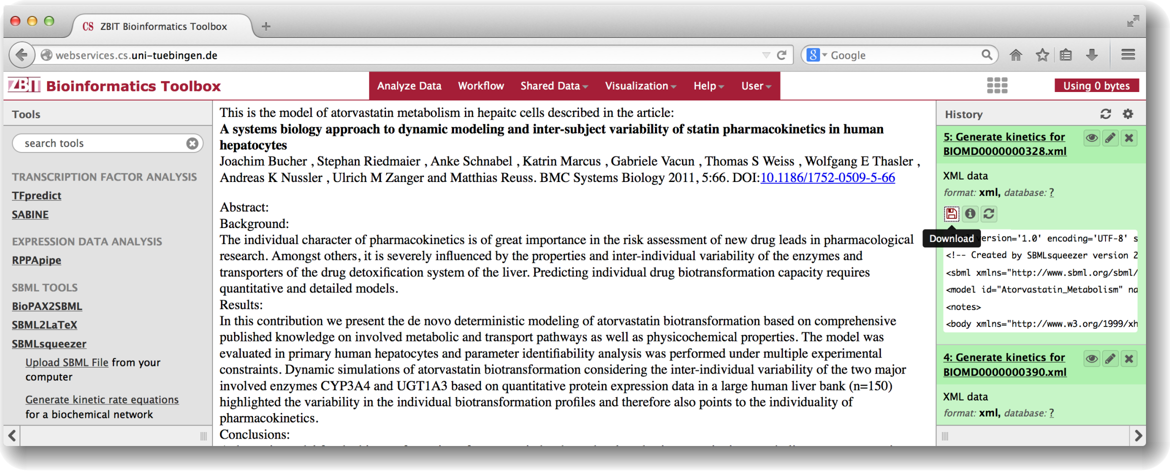

Figure 3.28: Obtaining the results from the online version. The history column contains an overview of all files for which kinetic equations have been generated. You can view the details of each file by clicking on the eye icon, or download the file by first clicking on the link in the headline of the respective entry ("Generated kinetics for...") and then clicking on the disk icon.

in order to download the SBML file that contains your results. If you click on the eye icon, the central column will display a rendered version of your SBML file, which is the view at all Extended HTML (XHTML) notes within your file.

4 Advanced program features

This chapter discusses how more experienced users can benefit from SBMLsqueezer's capabilities. In the first two sections we will have a look behind the scenes and explain how you can directly access and use the API of SBMLsqueezer in a programming environment. In the next section we will list and explain all possible command-line options. All program features that have been described in the previous chapter can also be addressed through the command-line and programming interface, making SBMLsqueezer a versatile tool that can be used in diverse, customized applications.

4.1 Using SBMLsqueezer as an equation generator library

We will now discuss how to use the API of SBMLsqueezer directly in order to generate kinetic equations as part of more complex operations. This function could already be used in version 1.3 of SBMLsqueezer (see listing A.1 on page 72). Since then, the API has been greatly modified and improved. We will now discuss important aspects of the new API of SBMLsqueezer and give examples of particularly important use-case scenarios.

4.1.1 Assignment of kinetic equations to all reactions in an SBML file

Listing 4.1 gives you an example for a simple application that takes the paths to two SBML files as input and proceeds as follows:

1. It changes the preferences of the program by defining several options.
2. It creates an instance of `SBMLsqueezer` with type parameter `org.sbml.jsbml.Model`, i.e., it will use JSBML as its parsing library.
3. By calling the `squeeze` method, it will then use JSBML to read the first file into an `SBMLDocument` data structure and generate kinetic equations for all reactions within the document according to the current preferences.
4. The result will be written into the second file, i.e., the original file will remain unchanged unless the two arguments point both to the identical file.

The paths in this example can also point to a directory instead of a particular file. If this is the case, `SBMLsqueezer` will recursively traverse all acceptable SBML files in the given input folder and create a corresponding file with newly created or obtained kinetic equations in the output folder.

```java
/**
 * This program takes the absolute paths to an input file and an output
 * file as arguments, configures several preferences for
 * {@link SBMLsqueezer}, generates kinetic equations, units, parameters
 * etc. and saves the result in the given output file in SBML format.
 *
 * @param args absolute or relative paths to two SBML files (in and out)
 * @throws Throwable if either user preferences cannot be made
 *         persistent or the creation of rate laws fails.
 */
public static void main(String[] args) throws Throwable {
  /*
   * Configure user preferences of SBMLsqueezer
   */
  // General preferences
  SBPreferences prefs =
      SBPreferences.getPreferencesFor(OptionsGeneral.class);
  prefs.put(OptionsGeneral.ALL_REACTIONS_AS_ENZYME_CATALYZED, true);
  prefs.put(OptionsGeneral.DEFAULT_COMPARTMENT_SIZE, 1d);
  prefs.put(OptionsGeneral.POSSIBLE_ENZYME_RNA, true);
  prefs.flush();

  // Rate law selection
  prefs = SBPreferences.getPreferencesFor(OptionsRateLaws.class);
  prefs.put(OptionsRateLaws.KINETICS_REVERSIBLE_UNI_UNI_TYPE,
      MichaelisMenten.class);
  prefs.put(OptionsRateLaws.KINETICS_REVERSIBLE_ARBITRARY_ENZYME_REACTIONS,
      ConvenienceKinetics.class);
  prefs.flush();

  // Initialize SBMLsqueezer with JSBML as its internal SBML library
  SBMLsqueezer<Model> squeezer = new SBMLsqueezer<Model>();
  // Create kinetic equations, parameters, units etc. and save the result
  squeezer.squeeze(args[0], args[1]);
}
```

Listing 4.1: Generating rate laws for all reactions in a model via the API of SBMLsqueezer 2.1

4.1.2 Adjusting configuration and user preferences

While the previous version of SBMLsqueezer stored its preferences in configuration files within the user's home directory, SBMLsqueezer now makes use of the preferences of your operating system. The keywords for the individual options have been slightly changed, but are still very similar. In general, the names of the command-line options (see section 4.3 on page 40) are very similar to the names of the options that you can use when hacking SBMLsqueezer's API. Furthermore, the grouping of the preferences is identical to the grouping of options you have in the tabs of the preferences dialog (see figs. 3.1 to 3.5 on pages 11–15) and also the groups of command-line

options. You can therefore read through the command-line options in order to get a description of potential options to be used in the API. The Java™ API documentation, which you can find online on the project's homepage[1], will also be helpful to gain an understanding of available options.

The options of SBMLsqueezer are each gathered in specialized interfaces, which all extend the interface `KeyProvider`. Each option is a constant field variable. If you are using an Integrated Development Environment (IDE), such as Eclipse, an auto-complete function will directly show you which options are provided within a particular sub-class of `KeyProvider`. Such an IDE will also display the Java documentation to you. You can load the preferences by calling

```
SBPreferences prefs =
   SBPreferences.getPreferencesFor(MyOptionsClass.class);
```

Listing 4.2: Loading preferences in SBMLsqueezer 2.1

Here, `MyOptionsClass` acts as a placeholder for your actual instance of `KeyProvider` for which you want to load preferences. SBMLsqueezer gathers its specific options in the following interfaces:

`org.sbml.squeezer.OptionsGeneral` See the command-line options described in section 4.3.3 on page 41.

`org.sbml.squeezer.io.IOOptions` These options are described in section 4.3.2 on page 41.

`org.sbml.squeezer.kinetics.OptionsRateLaws` Corresponds to the command-line arguments in section 4.3.4 on page 45.

`org.sbml.squeezer.sabiork.SABIORKOptions` See the description in section 4.3.5 on page 52.

`org.sbml.squeezer.sabiork.SABIORKPreferences` See section 4.3.6 on page 52.

Each option has a specific data type associated with it. You can hence use a call of the `put` method on your instance of `SBPreferences` to alter the value for an option. Thereby, the option is used as the key in the preferences object. In the same way, you can also obtain the current value for an option. Just read the documentation about preferences of the standard Java™ distribution[2]. The only difference is that SBMLsqueezer uses an extended version of the regular preferences implementation, which allows you to directly store and retrieve more complex data structures besides `String`s, such as `File` or `Class` objects. In particular, when selecting a specific kind of rate law for a certain type of reaction, SBMLsqueezer expects the class object of that `KineticLaw`. Note that this is in contrast to version 1.3, which expected the name of the class.

[1] `http://www.cogsys.cs.uni-tuebingen.de/software/SBMLsqueezer/doc/api/SBMLsqueezer2.1/`
[2] `http://docs.oracle.com/javase/6/docs/api/java/util/prefs/Preferences.html`

4.1.3 Using SBMLsqueezer with a libSBML back-end

Let us now discuss how you can use SBMLsqueezer's API with a libSBML back-end instead of pure JSBML. To this end, you only have to change the way how you initialize your instance of `SBMLsqueezer`:

```
SBMLsqueezer<org.sbml.libsbml.Model> squeezer = new
    SBMLsqueezer<org.sbml.libsbml.Model>(
  new org.sbml.jsbml.xml.libsbml.LibSBMLReader(),
  new org.sbml.jsbml.xml.libsbml.LibSBMLWriter());
```

Listing 4.3: Initializing SBMLsqueezer 2.1 with a libSBML back-end

Everything else of listing 4.1 on page 34 can be left unchanged. Here, we are initializing `SBMLsqueezer` based on a libSBML `Model` data structure. It is important to use the correct reader and writer from the package `org.sbml.jsbml.xml.libsbml`, and not to confuse these with the `LibSBMLReader` and `LibSBMLWriter` from the `org.sbml.jsbml.celldesigner.libsbml` package in JSBML. Otherwise, your application will not function, because the classes in this package are very basic implementations needed for the interface between JSBML and CellDesigner.

Please note that SBMLsqueezer can internally only work with the JSBML data structure. The libSBML back-end is used to parse and write SBML files and also allows for off-line model validation. However, SBMLsqueezer will convert libSBML's data structure to JSBML and synchronize its changes to the original data structure. When run-time is crucial for your application, this procedure should hence be avoided.

4.1.4 Assignment of specific rate laws to selected reactions

Instead of reading and writing entire files or recursively walking through a directory structure, it can also be desirable to perform more customized calls to create rate laws for individual reactions only. To this end, a deeper understanding about the functioning of SBMLsqueezer is required. There are a few particularly important classes for the *de novo* creation of kinetic equations, which are all located in the package `org.sbml.squeezer`:

`KineticLawGenerator` This class contains the actual algorithms to build a rate law and to synchronize changes between models.

`ParameterFactory` Here all (local) parameters are generated. The implementation follows the factory pattern, but it requires an instance of the model for which the parameters are created because of numerous dependencies between objects and IDs. It uses the unit factory (see below) to derive the units of new parameters. This class also annotates all newly created parameter objects with SBO terms.

`SubmodelController` SBMLsqueezer never modifies the original model data structure. Internally, this controller creates a minimal copy of the model, only comprising those reactions with dependent model components (required species, compartments, parameters, units, etc.)

that are needed to work with the currently selected set of reactions. In the GUI, the user can typically select one or all reactions of a model. This class is more powerful, as it could potentially generate a sub-model for an arbitrary set of reactions with all required other components of the model. It hence generates a restricted clone of the model.

`ReactionType` This class determines the base type of a given reaction and contains the algorithms that select appropriate rate laws or the most suitable rate law. It also removes substances from the reaction if these belong to the group of substances from the ignore list (see table 5.1 on page 61).

`UnitFactory` This factory class generates unit definition objects and IDs for unit definitions, which are to be assigned to parameters and numbers in kinetic expressions.

With these classes at hand, new kinetic equations can be created for arbitrary reactions. The example in listing 4.1 on page 34 demonstrates how a convenience rate law (Liebermeister and Klipp, 2006) can be assigned to a selected reaction in an SBML file. This type of rate equation can be applied to reversible as well as to irreversible enzyme-catalyzed reactions and has been found to be particularly useful when no specific information about reaction mechanisms is known (Dräger *et al.*, 2007a, 2009a; Dräger, 2011).

```
/**
 * Reads a given SBML file, creates a convenience rate law for the first
 * reaction within that file, and stores the result in the second file.
 *
 * @param args absolute or relative paths to two SBML files (in and out)
 * @throws Throwable
 */
public static void main(String args[]) throws Throwable {
  // Select an SBML reaction object from your SBML document
  SBMLDocument doc = SBMLReader.read(new File(args[0]));
  Model model = doc.getModel();
  Reaction reaction = model.getReaction(0);

  // Create and run the kinetic law generator
  KineticLawGenerator klg = new KineticLawGenerator(model);
  // This operates on a copy of the model that only contains one reaction
  klg.createKineticLaw(
    reaction,                     // the selected reaction
    ConvenienceKinetics.class,    // the type of rate law to be created
    false,                        // whether to set the reaction reversible
    TypeStandardVersion.cat,      // type parameter for modular rate laws
    UnitConsistencyType.amount,   // how to ensure unit consistency
    1d);                          // the value for new parameters

  // Transfer the rate law from the model copy to the original model
  klg.storeKineticLaws();

  // Save the result in another file
```

```java
    TidySBMLWriter.write(doc, new File(args[1]), ' ', (short) 2);
  }
```

Listing 4.4: Assignment of a kinetic law to a reaction via the API of SBMLsqueezer 2

4.2 Querying SABIO-RK and merging the results into a local SBML document

The command-line mode for the interface to SABIO-RK is implemented in the class `ConsoleWizard`. This class is located in the package `org.sbml.squeezer.sabiork.wizard.console`. The following example shows how to query for rate equations in SABIO-RK by using SBMLsqueezer's API. The search can either comprise all reactions or just the reactions in a model. All search terms that can be applied to the model are listed at the beginning of the source-code example. The KEGG (Kanehisa and Goto, 2000) ID of a reaction is automatically added to the search terms (compare section 3.6.1 on page 20). A found kinetic law is added to a reaction if all contained elements can be matched to SBML elements in the model.

```java
  /**
   * Reads a given SBMLDocument and runs the console mode. The reactions
   * are searched by their KEGG term and additional search terms.
   *
   * @param args
   *        absolute or relative paths to two SBML files (input and output)
   */
  public static void main(String[] args) throws Throwable{
    // The different search terms can be set here (null means that the term
    // is not used).
    String pathway = null;
    String tissue = null;
    String organism = null;
    String cellularLocation = null;
    Boolean isWildtype = true;
    Boolean isMutant = true;
    Boolean isRecombinant = false;
    Boolean hasKineticData = true;
    Double lowerpHValue = 7.9d;
    Double upperpHValue = 14d;
    Double lowerTemperature = -10d;
    Double upperTemperature = 115d;
    Boolean isDirectSubmission = true;
    Boolean isJournal = true;
    Boolean isEntriesInsertedSince = false;
    String dateSubmitted = "15/10/2008";

    boolean overwriteExistingRateLaws = true;

    // Read the SBMLDocument
```

```java
    SBMLDocument doc = SBMLReader.read(new File(args[0]));

    // Run the console mode
    SABIORKWizard.getResultConsole(doc, overwriteExistingRateLaws,
        pathway, tissue, organism,
      cellularLocation, isWildtype, isMutant, isRecombinant,
      hasKineticData, lowerpHValue, upperpHValue, lowerTemperature,
      upperTemperature, isDirectSubmission, isJournal,
      isEntriesInsertedSince, dateSubmitted);

    // Save the changed document
    TidySBMLWriter.write(doc, new File(args[1]), '␣', (short) 2);
  }
```

Listing 4.5: SABIO-RK query for the reactions of a model

You can also launch the GUI of the SABIO-RK wizard by using SBMLsqueezer's API. The following source-code example demonstrates how to do so.

```java
/**
 * Runs the wizard
 *
 * @param input  the input file
 * @param output the output file
 */
public static void runGUI(final File input, final File output) {
  try {
    UIManager.setLookAndFeel(UIManager.getSystemLookAndFeelClassName());
  } catch (Exception exc) {
    exc.printStackTrace();
  }
  final JFrame frame = new JFrame("SBMLsqueezer");
  frame.setLayout(new BorderLayout());
  frame.setMinimumSize(new Dimension(300, 300));

  JButton buttonWizard = new JButton("Open␣SABIO-RK␣Wizard");
  buttonWizard.addActionListener(new ActionListener() {
    @Override
    public void actionPerformed(ActionEvent e) {
      SBMLDocument result = null;
      try {
        result = SABIORKWizard.getResultGUI(frame,
          ModalityType.APPLICATION_MODAL,
          SBMLReader.read(input), true).getSBMLDocument();
      } catch (XMLStreamException e1) {
        e1.printStackTrace();
      } catch (IOException e1) {
        e1.printStackTrace();
      }
      try {
        if (result != null) {
```

```
          TidySBMLWriter.write(result, output, ' ', (short) 2);
        }
      } catch (SBMLException e1) {
        e1.printStackTrace();
      } catch (XMLStreamException e1) {
        e1.printStackTrace();
      } catch (IOException e1) {
        e1.printStackTrace();
      }
    }
  });

  frame.add(buttonWizard, BorderLayout.NORTH);
  frame.setVisible(true);
}
```

Listing 4.6: Calling the SABIO-RK wizard

4.3 Command-line arguments

All functions of the program SBMLsqueezer are also available as command-line arguments. SBMLsqueezer is therefore fully functional even if you do not use its GUI. This can be useful if you like to generate kinetic equations, units, and parameter objects for a large number of SBML files in a loop or in a more complex schedule. To benefit from this functionality, just open a command line window on your operating system, e.g., execute the `cmd.exe` in Microsoft Windows or open a terminal window in Unix (incl. Linux and Mac OS X).

It should also be noted that the command line options impact the GUI. It is hence possible to use command-line options in order to launch SBMLsqueezer with a pre-defined configuration. However, you should note that if you pass a command-line argument to SBMLsqueezer, it might assume that you intend to use the program in the pure command-line mode and hence will not launch the GUI. In this case, you will need to also pass the command-line option `--gui=true` or just `--gui` to the program.

4.3.1 Program usage

You can start the application on all operating systems by typing

```
java -jar SBMLsqueezer.jar [options]
```

Listing 4.7: Launching SBMLsqueezer from the command-line

on your command prompt. Please note that you might have to change `SBMLsqueezer.jar` for the real name of the JAR file, e.g., `SBMLsqueezer_v2.1.jar`. For future versions of SBMLsqueezer the name of the JAR file could also change, because it usually includes the version number.

In most cases, SBMLsqueezer needs more than 128 MB memory, so it might be convenient to create a shortcut and start the application with as much memory as available. If you have 2 GB RAM, for example, you might want to start the application with the following command:

```
java -Xms128m -Xmx1400M -jar SBMLsqueezer.jar [options]
```

Listing 4.8: Launching SBMLsqueezer from the command-line with increased heap size

How much memory you actually need strongly depends on the size of your input datasets. Under Microsoft Windows you might want to type `javaw` instead of `java` in order to be able to close the command-line window while the program is running. This feature is particularly useful when writing a batch script to launch SBMLsqueezer with your favorite command-line arguments.

Use the following program parameters to lists all available options of SBMLsqueezer: `--help` or `-?`. The subsequent sections describe all available options of SBMLsqueezer version 2.1.

SBMLsqueezer's entire user interface (the GUI as well as the command line) is equipped with with language packs for German and English, and partially for Chinese (Mandarin). By default the program checks your system's configuration and loads the language pack of your operating system. The fallback language is English. You can also purposely choose the language of the user interface by passing the language parameter as an argument to the Java™ Virtual Machine (JVM). To this end use the following launch command

```
java -Xms128m -Xmx1400M -Duser.language=en -jar SBMLsqueezer.jar [options]
```

Listing 4.9: Launching SBMLsqueezer with an alternative language pack

if you want to launch the program with an English user interface. For German use `de`, and `zh` for Chinese.

4.3.2 Input/output options

`--sbml-in-file[ |=]<File>` Specifies the SBML input file. Default value: none

`--sbml-out-file[ |=]<File>` Specifies the file where SBMLsqueezer writes its SBML output. Default value: none

`--try-loading-libsbml` If selected, the application will try to load the library libSBML for reading and writing SBML files, otherwise everything will be done with JSBML only, i.e., pure Java™ and therefore platform independent. Default value: `false`

4.3.3 Basic configuration

With the options introduced in this section, you can specify which assumptions SBMLsqueezer should make when values or information are missing, i.e., if the model is lacking more information than just kinetic equations. You can also specify how to interpret the model and when error messages should be displayed.

General Options

The behavior of SBMLsqueezer can be specified by the following options, for instance, to decide if error messages should be displayed under certain circumstances.

`--overwrite-existing-rate-laws[ |=]<Boolean>` If this flag is set to `true`, a new rate law will be created for each reaction irrespective of whether there is already a rate law assigned to this reaction or not. If `false`, new rate laws are only generated if missing in the SBML file. Note that if this option is checked, already existing kinetic laws will be overwritten. Default value: `true`

`--set-boundary-condition-for-genes[ |=]<Boolean>` If `true`, the boundary condition of all species that represent gene-coding elements, such as genes or gene-coding regions, will be set to `true`. Default value: `true`

`--all-reactions-as-enzyme-catalyzed[ |=]<Boolean>` If `true`, all reactions within the network are considered to be enzyme-catalyzed reactions. If `false`, an explicit enzymatic catalyst must be assigned to a reaction to obtain this status. Default value: `true`

`--remove-unnecessary-parameters-and-units[ |=]<Boolean>` If `true`, parameters and units that are never referenced by any element of the model are automatically deleted after creating kinetic equations. Default value: `true`

`--new-parameters-global` If `true`, all parameters are stored globally for the whole model. Otherwise the majority of parameters is stored locally for the respective kinetic equation they belong to. Note that some parameters represent global properties of the entire model and should therefore be always stored in the global list of parameters. In this way, these parameters are valid within the entire model. Default value: `false`

`--warnings-for-too-many-reactants[ |=]<Boolean>` If `true`, warnings will be displayed for reactions with an unrealistic number of reactants. The maximal number of reactants that are believed to be still realistic can be defined if this option is selected. Default value: `true`

`--show-sbml-warnings[ |=]<Boolean>` If `true`, SBML warnings are displayed. These warnings are mainly the result of a syntactical model check and do not give much information about the semantic correctness of your model. Since the SBML library performs this check, SBMLsqueezer cannot influence the content of the syntax check. Note that this option only works properly if you use libSBML as your SBML back-end, because JSBML does not provide a full validity check for SBML models. Default value: `true`

`--read-from-sabio-rk[ |=]<Boolean>` This option lets the user choose whether to search for experimentally obtained rate laws in the reaction kinetics database SABIO-RK. Note that performing this search requires an active Internet connection. Default value: `true`

Default values

The options in this group allow you to specify several default values to be applied for components of the model. This is done in addition to the actual rate law generation, such as the default compartment size, etc., and to define model wide settings to be taken into account when creating rate equations.

`--max-number-of-reactants[ |=]<Integer>` A simultaneous collision of a high number of reactants just by chance is very unlikely. Usually, these reactions proceed in a sequence of separate steps, each involving only very few molecules. Here you can specify the maximal number of reactants so that the reaction is still considered plausible. Note that this option is only available if you decide that this kind of warning should be displayed. Default value: `3`

`--default-compartment-spatial-dim[ |=]<Double>` If no spatial dimensions are defined for a compartment, the value defined by this option will be used as a default. Default value: `3.0`

`--default-compartment-size[ |=]<Double>` For compartments that are not yet initialized, SBMLsqueezer will use this value as the default initial size. Default value: `1.0`

`--default-species-init-val[ |=]<Double>` If species are not yet initialized, SBMLsqueezer will use this value as initial amount or initial concentration of the species. Which kind of quantity is used, depends on whether the species has only substance units. This means, for species that are to be interpreted in terms of concentration, an initial concentration will be set, whereas an initial amount will be set if the species is to be interpreted in terms of molecule counts. Default value: `1.0`

`--default-new-parameter-val[ |=]<Double>` Here you can specify the default value that is set for newly created parameters. Default value: `1.0`

`--default-species-has-only-substance-units[ |=]<Boolean>` This option allows users to specify that the numerical value of a species should be interpreted as a value given in substance units in cases where this has not yet been defined. If not selected, species with undefined meaning will be conceived as a quantity in concentration units. Default value: `true`

`--ignore-these-species-when-creating-laws[ |=]<String>` This option allows the user to ignore species that are annotated with the given compound IDs when creating rate laws for reactions that involve these species. For instance, water or single protons can often be ignored when creating rate equations, hence simplifying the resulting rate equations. Preselected are the KEGG (Kanehisa and Goto, 2000) compound IDs for several ions and small molecules, including water and protons. See table 5.1 on page 61 for details. Default value: `C00001, C00038, C00070, C00076, C00080, C00175, C00238, C00282, C00291, C01327, C01528, C14818, C14819`

Species to be treated as enzymes

In many situations, it is not clear, which kinds of chemical species can be considered an enzyme. The following options allow you to select, which kind of species SBMLsqueezer should interpret as an enzyme when acting as a catalytic modifier of a reaction.

`--possible-enzyme-antisense-rna[ |=]<Boolean>` If this is set to `true`, anti-sense Ribonucleic Acid (RNA) molecules are treated as enzymes when catalyzing a reaction. If `false` anti-sense RNA molecule catalyzed reactions are not considered to be enzyme-catalyzed reactions. Default value: `false`

`--possible-enzyme-complex[ |=]<Boolean>` If checked, complex molecules are treated as enzymes when catalyzing a reaction. Otherwise, complex-catalyzed reactions are not considered to be enzyme reactions. Default value: `true`

`--possible-enzyme-generic[ |=]<Boolean>` If `true`, generic proteins are treated as enzymes when catalyzing a reaction. Otherwise, generic protein-catalyzed reactions are not considered to be enzyme reactions. Default value: `true`

`--possible-enzyme-macromolecule[ |=]<Boolean>` If this options is selected, species that are annotated as macromolecules are treated as enzymes when catalyzing a reaction. Otherwise, macromolecule-catalyzed reactions are not considered enzyme reactions. If a modifier of a reaction that is annotated as an enzymatic catalyst refers to a macromolecule but this option is not active, SBMLsqueezer will reduce the modifier to a simple catalyst. Default value: `true`

`--possible-enzyme-receptor[ |=]<Boolean>` If `true`, receptors are treated as enzymes when catalyzing a reaction. If `false`, receptor-catalyzed reactions are not considered to be enzyme reactions. Default value: `false`

`--possible-enzyme-rna[ |=]<Boolean>` If `true`, RNA is treated as an enzyme when catalyzing a reaction. Otherwise RNA-catalyzed reactions are not considered to be enzyme-catalyzed reactions. Default value: `true`

`--possible-enzyme-simple-molecule` If `true`, simple molecules are treated as enzymes when catalyzing a reaction. If `false`, simple molecule-catalyzed reactions are not considered to be enzyme reactions. Default value: `false`

`--possible-enzyme-truncated[ |=]<Boolean>` If `true`, truncated proteins are treated as enzymes when catalyzing a reaction. Otherwise, truncated protein-catalyzed reactions are not considered to be enzyme reactions. Default value: `true`

`--possible-enzyme-unknown` If `true`, unknown molecules are treated as enzymes when catalyzing a reaction. If `false`, unknown molecule-catalyzed reactions are not considered to be enzyme reactions. Default value: `false`

How to ensure unit consistency

Unit consistency is an important property for kinetic models. In SBML, the kinetics of all reactions should be defined so that these can be evaluated to units of substance per time. Since Level 3, the extend units of a model can be defined. Since then, evaluating reactions should result in extend units per time units. The main difficulty when dealing with units are transport reactions and the ability to specify reactive species in terms of molecule counts (amounts) or concentration units, which is amounts per size. In addition, the sizes of compartments do not necessarily have to be three dimensional. In SBML, the spatial dimensions do not have to be integers either. However, SBMLsqueezer contains several complex algorithms to determine appropriate units for newly generated parameters and to incorporate the sizes of surrounding compartments into the generated rate laws. Here you can specify how to do that.

`--type-unit-consistency[ |=]<UnitConsistencyType>` This option ensures unit consistency and can attain two different values: Choose *amount* to bring each occurrence of a participating species to a substance unit. Depending on whether the species has only substance units or not it might be necessary to multiply the species with the size of its surrounding compartment. Choose *concentration* to bring each participating species to concentration units. In this case the species will be divided by the surrounding compartment size in a kinetic equations if it is defined to have only substance units. The units of parameters are set accordingly. All possible values for type `<UnitConsistencyType>` are: `amount` and `concentration`. Default value: `amount`

4.3.4 Rate law selection

The options in this group allow you to define the rate laws with highest priority when generating kinetic equations in one single step for an entire network. To this end, SBMLsqueezer defines several basic types of reactions and provides a list of applicable generic equations for each type. Special cases of these equations have to be derived for each individual case, also depending on unit consistency, compartment dimensions, etc. The actual selection of a rate law means that you can specify, which class of the program SBMLsqueezer should be used to generate a rate law of this type. You do not need to have deep programming skills for this. All you need to know here is that the the names of the rate laws are a bit more complicated than the actual human-readable names, because you must specify, how SBMLsqueezer internally names these rate laws. For more advanced users, it might be interesting to know that SBMLsqueezer uses the concept known as *reflection* to select its kinetic equations.

Furthermore, you can decide how to deal with information about reversible or irreversible reactions.

Reversibility

These two options are mutually exclusive and their values must not contradict. Use either one of both.

`--treat-all-reactions-reversible` If `true`, all reactions are set to reversible before creating new kinetic equations. Otherwise, the information given by the SBML file will be left unchanged. Default value: `false`

`--treat-reactions-reversible-as-given[ |=]<Boolean>` If checked, the information about reversibility will be left unchanged. Default value: `true`

Gene regulation kinetics

`--kinetics-gene-regulation[ |=]<Class>` Please specify the default kinetic law to be applied for reactions that are identified to belong to gene-regulatory processes (reactions involving genes, RNA, and proteins), such as transcription or translation. All possible values for type `<Class>` are:

- `org.sbml.squeezer.kinetics.AdditiveModelLinear` (general form linear additive model),
- `org.sbml.squeezer.kinetics.AdditiveModelNonLinear` (general form of the non-linear additive model),
- `org.sbml.squeezer.kinetics.NetGeneratorLinear` (Net*Gene*rator form of the linear additive model),
- `org.sbml.squeezer.kinetics.HillEquation` (generalized Hill equation),
- `org.sbml.squeezer.kinetics.HillHinzeEquation` (Hill-Hinze equation),
- `org.sbml.squeezer.kinetics.HillRaddeEquation` (Hill-Radde equation),
- `org.sbml.squeezer.kinetics.HSystem` (H-system equation by Spieth *et al.*, 2006),
- `org.sbml.squeezer.kinetics.NetGeneratorNonLinear` (Net*Gene*rator form of the non-linear additive model),
- `org.sbml.squeezer.kinetics.SSystem` (S-System-based kinetic),
- `org.sbml.squeezer.kinetics.Vohradsky` (Non-linear additive model by Vu and Vohradský, 2007), and
- `org.sbml.squeezer.kinetics.Weaver` (non-linear additive model by Weaver *et al.*, 1999).

Default value: `org.sbml.squeezer.kinetics.HillHinzeEquation`

`--kinetics-zero-reactants[ |=]<Class>` Default rate law with zeroth order reactants. All possible values for type `<Class>` are:

- `org.sbml.squeezer.kinetics.AdditiveModelLinear` (general form of the linear additive model),
- `org.sbml.squeezer.kinetics.AdditiveModelNonLinear` (general form of the non-linear additive model),

- `org.sbml.squeezer.kinetics.HillHinzeEquation` (Hill-Hinze equation),
- `org.sbml.squeezer.kinetics.HillRaddeEquation` (Hill-Radde equation),
- `org.sbml.squeezer.kinetics.HSystem` (H-system equation by Spieth *et al.*, 2006),
- `org.sbml.squeezer.kinetics.NetGeneratorLinear` (Net*Gene*rator form of the linear additive model),
- `org.sbml.squeezer.kinetics.NetGeneratorNonLinear` (Net*Gene*rator form of the non-linear additive model),
- `org.sbml.squeezer.kinetics.SSystem` (S-System-based kinetic),
- `org.sbml.squeezer.kinetics.Vohradsky` (Non-linear additive model by Vu and Vohradský, 2007),
- `org.sbml.squeezer.kinetics.Weaver` (non-linear additive model by Weaver *et al.*, 1999),
- `org.sbml.squeezer.kinetics.ZerothOrderForwardGMAK` (zeroth order forward mass action kinetics), and
- `org.sbml.squeezer.kinetics.ZerothOrderReverseGMAK` (zeroth order reverse mass action kinetics).

Default value: `org.sbml.squeezer.kinetics.ZerothOrderReverseGMAK`

`--kinetics-zero-products[ |=]<Class>` Default rate law with zeroth order products All possible values for type `<Class>` are:

- `org.sbml.squeezer.kinetics.AdditiveModelLinear` (general form of the linear additive model),
- `org.sbml.squeezer.kinetics.AdditiveModelNonLinear` (general form of the non-linear additive model),
- `org.sbml.squeezer.kinetics.HillHinzeEquation` (Hill-Hinze equation),
- `org.sbml.squeezer.kinetics.HillRaddeEquation` (Hill-Radde equation),
- `org.sbml.squeezer.kinetics.HSystem` (Hill-Radde equation),
- `org.sbml.squeezer.kinetics.NetGeneratorLinear` (Net*Gene*rator form of the linear additive model),
- `org.sbml.squeezer.kinetics.NetGeneratorNonLinear` (Net*Gene*rator form of the non-linear additive model),
- `org.sbml.squeezer.kinetics.SSystem` (S-System-based kinetic),
- `org.sbml.squeezer.kinetics.Vohradsky` (Non-linear additive model by Vu and Vohradský, 2007),

- `org.sbml.squeezer.kinetics.Weaver` (non-linear additive model by Weaver *et al.*, 1999),
- `org.sbml.squeezer.kinetics.ZerothOrderForwardGMAK` (zeroth order forward mass action kinetics), and
- `org.sbml.squeezer.kinetics.ZerothOrderReverseGMAK` (zeroth order reverse mass action kinetics).

Default value: `org.sbml.squeezer.kinetics.ZerothOrderReverseGMAK`

Reversible rate laws

`--type-standard-version[ |=]<TypeStandardVersion>` This option declares the version of the modular rate laws and can attain the three different values *cat*, *hal*, and *weg* as described in the publication of Liebermeister *et al.* (2010). This option can only be accessed if all reactions are modeled reversibly. All possible values for type `<TypeStandardVersion>` are: `cat`, `hal`, and `weg`. Default value: `cat`

`--kinetics-reversible-non-enzyme-reactions[ |=]<Class>` Determines the key for the standard kinetic law to be applied for reactions that are catalyzed by non-enzymes or that are not catalyzed at all. The value may be any rate law that implements `InterfaceNonEnzymeKinetics` All possible values for type `<Class>` are:

- `org.sbml.squeezer.kinetics.GeneralizedMassAction` (the generalized mass-action rate law),
- `org.sbml.squeezer.kinetics.ZerothOrderForwardGMAK` (zeroth order forward mass action kinetics), and
- `org.sbml.squeezer.kinetics.ZerothOrderReverseGMAK` (zeroth order reverse mass action kinetics).

Default value: `org.sbml.squeezer.kinetics.GeneralizedMassAction`

`--kinetics-reversible-uni-uni-type[ |=]<Class>` This key defines the default kinetic law to be applied to enzyme-catalyzed reactions with one reactant and one product. All possible values for type `<Class>` are:

- `org.sbml.squeezer.kinetics.CommonModularRateLaw` (CM: the common modular rate law),
- `org.sbml.squeezer.kinetics.ConvenienceKinetics` (convenience kinetics),
- `org.sbml.squeezer.kinetics.DirectBindingModularRateLaw` (DM: the direct binding modular rate law),
- `org.sbml.squeezer.kinetics.ForceDependentModularRateLaw` (FM: the force-dependent modular rate law),

- `org.sbml.squeezer.kinetics.HillEquation` (generalized Hill equation),
- `org.sbml.squeezer.kinetics.MichaelisMenten` (Michaelis-Menten),
- `org.sbml.squeezer.kinetics.PowerLawModularRateLaw` (PM: the power-law modular rate law), and
- `org.sbml.squeezer.kinetics.SimultaneousBindingModularRateLaw` (SM: the simultaneous binding modular rate law).

Default value: `org.sbml.squeezer.kinetics.MichaelisMenten`

`--kinetics-reversible-bi-uni-type[ |=]<Class>` Choose the type of the default kinetic law for reversible bi-uni reactions (two reactants, one product). All possible values for type `<Class>` are:

- `org.sbml.squeezer.kinetics.CommonModularRateLaw` (CM: the common modular rate law),
- `org.sbml.squeezer.kinetics.ConvenienceKinetics` (convenience kinetics),
- `org.sbml.squeezer.kinetics.DirectBindingModularRateLaw` (DM: the direct binding modular rate law),
- `org.sbml.squeezer.kinetics.ForceDependentModularRateLaw` (FM: the force-dependent modular rate law),
- `org.sbml.squeezer.kinetics.OrderedMechanism` (ordered mechanism),
- `org.sbml.squeezer.kinetics.PowerLawModularRateLaw` (PM: the power-law modular rate law),
- `org.sbml.squeezer.kinetics.RandomOrderMechanism` (random order mechanism), and
- `org.sbml.squeezer.kinetics.SimultaneousBindingModularRateLaw` (SM: the simultaneous binding modular rate law).

Default value: `org.sbml.squeezer.kinetics.RandomOrderMechanism`

`--kinetics-reversible-bi-bi-type[ |=]<Class>` Select the type of the default kinetic law for reversible bi-bi reactions (two reactants, two products). All possible values for type `<Class>` are:

- `org.sbml.squeezer.kinetics.CommonModularRateLaw` (CM: the common modular rate law),
- `org.sbml.squeezer.kinetics.ConvenienceKinetics` (convenience kinetics),
- `org.sbml.squeezer.kinetics.DirectBindingModularRateLaw` (DM: the direct binding modular rate law),

- org.sbml.squeezer.kinetics.ForceDependentModularRateLaw (FM: the force-dependent modular rate law),
- org.sbml.squeezer.kinetics.OrderedMechanism (ordered mechanism),
- org.sbml.squeezer.kinetics.PingPongMechanism (Ping-Pong mechanism),
- org.sbml.squeezer.kinetics.PowerLawModularRateLaw (PM: the power-law modular rate law),
- org.sbml.squeezer.kinetics.RandomOrderMechanism (random order mechanism), and
- org.sbml.squeezer.kinetics.SimultaneousBindingModularRateLaw (SM: the simultaneous binding modular rate law).

Default value: org.sbml.squeezer.kinetics.RandomOrderMechanism

--kinetics-reversible-arbitrary-enzyme-reactions[|=]<Class> Arbitrary reversible enzyme reactions. All possible values for type <Class> are:

- org.sbml.squeezer.kinetics.CommonModularRateLaw (common modular rate law, CM),
- org.sbml.squeezer.kinetics.ConvenienceKinetics (convenience kinetics),
- org.sbml.squeezer.kinetics.DirectBindingModularRateLaw (direct binding modular rate law, DM),
- org.sbml.squeezer.kinetics.ForceDependentModularRateLaw (force-dependent modular rate law, FM),
- org.sbml.squeezer.kinetics.PowerLawModularRateLaw (power-law modular rate law, PM), and
- org.sbml.squeezer.kinetics.SimultaneousBindingModularRateLaw (simultaneous binding modular rate law, SM).

Default value: org.sbml.squeezer.kinetics.CommonModularRateLaw

Irreversible rate laws

--kinetics-irreversible-non-enzyme-reactions[|=]<Class> Determines the key for the standard kinetic law to be applied for reactions that are catalyzed by non-enzymes or that are not catalyzed at all. The value may be any rate law that implements the interface org.sbml.squeezer.kinetics.InterfaceNonEnzymeKinetics All possible values for type <Class> are:

- org.sbml.squeezer.kinetics.GeneralizedMassAction (the generalized mass-action rate law),

- `org.sbml.squeezer.kinetics.ZerothOrderForwardGMAK` (zeroth order forward mass action kinetics), and
- `org.sbml.squeezer.kinetics.ZerothOrderReverseGMAK` (zeroth order reverse mass action kinetics).

Default value: `org.sbml.squeezer.kinetics.GeneralizedMassAction`

`--kinetics-irreversible-uni-uni-type[ |=]<Class>` This key defines the default kinetic law to be applied to enzyme-catalyzed reactions with one reactant and one product. All possible values for type `<Class>` are:

- `org.sbml.squeezer.kinetics.ConvenienceKinetics` (convenience kinetics),
- `org.sbml.squeezer.kinetics.IrrevCompetNonCooperativeEnzymes` (the irreversible non-exclusive non-cooperative competitive inhibition),
- `org.sbml.squeezer.kinetics.IrrevNonModulatedNonInteractingEnzymes` (a rate law for irreversible non-modulated non-interacting reactant enzymes),
- `org.sbml.squeezer.kinetics.HillEquation` (generalized Hill equation), and
- `org.sbml.squeezer.kinetics.MichaelisMenten` (Michaelis-Menten).

Default value: `org.sbml.squeezer.kinetics.MichaelisMenten`

`--kinetics-irreversible-bi-uni-type[ |=]<Class>` Choose the type of the default kinetic law for irreversible bi-uni reactions (two reactants, one product). All possible values for type `<Class>` are:

- `org.sbml.squeezer.kinetics.ConvenienceKinetics` (convenience kinetics),
- `org.sbml.squeezer.kinetics.IrrevNonModulatedNonInteractingEnzymes` (a rate law for irreversible non-modulated non-interacting reactant enzymes),
- `org.sbml.squeezer.kinetics.OrderedMechanism` (the ordered mechanism), and
- `org.sbml.squeezer.kinetics.RandomOrderMechanism` (the random order mechanism).

Default value: `org.sbml.squeezer.kinetics.RandomOrderMechanism`

`--kinetics-irreversible-bi-bi-type[ |=]<Class>` Select the type of the default kinetic law for irreversible bi-bi reactions (two reactants, two products). All possible values for type `<Class>` are:

- `org.sbml.squeezer.kinetics.ConvenienceKinetics` (convenience kinetics),
- `org.sbml.squeezer.kinetics.IrrevNonModulatedNonInteractingEnzymes` (a rate law for irreversible non-modulated non-interacting reactant enzymes),
- `org.sbml.squeezer.kinetics.OrderedMechanism` (ordered mechanism),

- `org.sbml.squeezer.kinetics.PingPongMechanism` (Ping-Pong mechanism), and
- `org.sbml.squeezer.kinetics.RandomOrderMechanism` (random order mechanism).

Default value: `org.sbml.squeezer.kinetics.RandomOrderMechanism`

`--kinetics-irreversible-arbitrary-enzyme-reactions[ |=]<Class>` Arbitrary irreversible enzyme reactions All possible values for type `<Class>` are:

- `org.sbml.squeezer.kinetics.ConvenienceKinetics` (convenience kinetics) and
- `org.sbml.squeezer.kinetics.IrrevNonModulatedNonInteractingEnzymes` (a rate law for irreversible non-modulated non-interacting reactant enzymes).

Default value: `org.sbml.squeezer.kinetics.IrrevNonModulatedNonInteractingEnzymes`

4.3.5 SABIO-RK search options

For querying the content of the SABIO-RK database for experimentally determined kinetic equations, SBMLsqueezer provides a large set of options in order to customize your search.

General options

With these options you can define key options to restrict the results of your search for reaction kinetics in SABIO-RK.

`--pathway[ |=]<String>` Define the pathway for which the kinetics are to be determined.

`--tissue[ |=]<String>` Define the tissue for which the kinetics are to be determined.

`--cellular-location[ |=]<String>` Define the cellular location for which the kinetics are to be determined.

`--organism[ |=]<String>` Define the organism for which the kinetics are to be determined.

4.3.6 SABIO-RK search preferences

General properties

`--is-wildtype[ |=]<Boolean>` Search for wild-type kinetics. Default value: `true`

`--is-mutant[ |=]<Boolean>` Search for kinetics of mutants. Default value: `true`

`--is-recombinant` Search for kinetics of recombinant organisms. Default value: `false`

`--has-kinetic-data[ |=]<Boolean>` Search for entries containing kinetic data. Default value: `true`

`--is-direct-submission[ |=]<Boolean>` Search for entries directly submitted. Default value: `true`

`--is-journal[ |=]<Boolean>` Search for entries referring to journal publications. Default value: `true`

`--is-entries-inserted-since` Consider only entries inserted after the specified date. Default value: `false`

Temperature

`--lowest-temperature-value[ |=]<Double>` The lowest possible temperature for entries (in °C). Arguments must fit into the range [-271.15, 1000]. Default value: `-10.0`

`--highest-temperature-value[ |=]<Double>` The highest possible temperature for entries (in °C). Arguments must fit into the range [-271.15, 1000]. Default value: `115.0`

Range of pH values

`--lowest-ph-value[ |=]<Double>` The lowest possible pH value for entries. Arguments must fit into the range [0, 14]. Default value: `0.0`

`--highest-ph-value[ |=]<Double>` The highest possible pH value for entries. Arguments must fit into the range [0, 14]. Default value: `14.0`

Date

`--lowest-date[ |=]<Date>` Define the earliest acceptable date when the entries have been inserted into SABIO-RK. Default value: `Wed Oct 15 00:00:00 PDT 2008`

4.3.7 Options for the Graphical User Interface

The options in this group allow you to influence the behavior of the GUI. This means that you can directly launch SBMLsqueezer with your preferred configuration.

`--check-for-updates[ |=]<Boolean>` Decide whether or not this program should search for updates at start-up. Default value: `true`

`--gui` If this option is given, the program will display its GUI. Default value: `false`

`--log-level[ |=]<String>` Change the log-level of this application. This option will influence how fine-grained error and other log messages will be that you receive while executing this program. Log messages whose level exceeds the given threshold will also be displayed in the status bar of the GUI. All possible values for type `<String>` are:

- `ALL`: all log messages will be displayed,
- `CONFIG`: those log messages related to configuration of the program and less fine-grained will be displayed,
- `FINE`: displays simple debugging messages and less fine-grained messages,
- `FINER`: the messages that will be displayed are already relevant for more extensive debugging purposes and less fine-grained messages,
- `FINEST`: information relevant for intensive debugging the program and less fine-grained will be displayed,
- `INFO`: information messages and less fine-grained messages, such as warning messages, will be displayed,
- `OFF`: no log messages will be displayed at all,
- `SEVERE`: only serious error messages will be displayed, and
- `WARNING`: only warnings and serious error messages will be displayed.

Default value: `INFO`

4.3.8 LaTeX options

The program SBMLsqueezer brings with it a full version of the latest development release of SBML2LaTeX. Hence, all options provided by this report generator for SBML models can also be applied to SBMLsqueezer. For more information, see the project web site of SBML2LaTeX[3] and the corresponding publication (Dräger *et al.*, 2009b).

LaTeX compiler location

`--load-latex-compiler[ |=]<File>` The path to the LaTeX compiler to generate PDF, DVI or other files from the created LaTeX report file. Accepts all files (*). Default value: `<current directory>`

Report options

`--check-consistency` If `true`, the automatic model consistency check is performed and the results are written in the appendix of the model report file. Note that this might require an active Internet connection. Default value: `false`

`--miriam-annotation[ |=]<Boolean>` If `true`, MIRIAM annotations are included into the model report if there are any. In this case, SBML2LaTeX generates links to the resources for each annotated element. Default value: `true`

[3] `http://www.cogsys.cs.uni-tuebingen.de/software/SBML2LaTeX/`

--show-predefined-units[|=]<Boolean> If true, all the predefined unit declarations of the model are made explicit in the report file as these are defined by the corresponding SBML Level and Version. Otherwise only unit definitions from the model are included. Note that this option is only available if the option --include-section-unit-definitions is active. Default value: true

--print-full-ode-system If set to true, the entire rate of change will be written for each species. By default, SBML2LaTeX only prints the sum of the individual reaction rates, which are hyper-linked but displayed at a different position of the report. Note that this option is only available if the option --include-section-reactions is active. Default value: false

--clean-workspace If this option is set to true, all temporary files will be deleted after running SBML2LaTeX. In case of PDF creation, for instance, this will cause even the TeX file to be deleted. However, this option can be meaningful to remove all the temporary files created by your system's LaTeX compiler. Default value: false

Layout options

--landscape This option decides whether to set the LaTeX document in landscape or portrait mode. By default most pages are in portrait format. Default value: false

--print-names-if-available If selected, the names of SBML elements (NamedSBase) are displayed instead of their IDs. This can only be done if the element has a name. Default value: false

--title-page If true, a separate title page will be created. By default the title is written as a simple heading on the first page. Default value: false

--typewriter[|=]<Boolean> This option decides whether a typewriter font should be applied to highlight SBML IDs. This is particularly important when these occur in mathematical equations. Default value: true

--reactants-overview-table If true, the details (ID and name) of all reactants, modifiers and products participating in a reaction are listed in one table. By default a separate table is created for each one of the three participant groups including its SBO term. Note that this option is only available if the option --include-section-reactions is active. Default value: false

Typographical options

--font-headings[|=]<SansSerifFont> Allows to select the font of captions and other (by default sans serif) text. All possible values for type <SansSerifFont> are:

- avant (sample in Avant Garde),
- cmss (sample in Computer Modern Sans Serif), and
- helvetica (sample in Helvetica).

Default value: helvetica

--font-size[|=]<Short> This option allows you to select the size of the standard text font. Headings appear with a larger font. All possible values for type <Short> are: 8, 9, 10, 11, 12, 14, and 17. Default value: 11

--font-text[|=]<SerifFont> Allows to select the font of continuous text. Choosing times is actually not recommended because in some cases equations might not look as nicely as they do when using mathptmx. All possible values for type <SerifFont> are:

- chancery *(sample in Chancery)*,
- charter (sample in Charter),
- cmr (sample in Computer Modern Roman),
- mathptmx (sample in Times for math),
- palatino (sample in Palatino),
- times (sample in Times), and
- utopia (sample in Utopia).

Default value: mathptmx

--font-typewriter[|=]<String> Select a typewriter font that can be used for IDs if option 'TYPEWRITER' is selected. URLs and other resources are also marked with this font. All possible values for type <String> are:

- cmt (sample in Computer Modern Typewriter) and
- courier (sample in Courier).

Default value: cmt

--paper-size[|=]<PaperSize> The paper size for LaTeX documents. With this option the paper format can be influenced. Default paper size: DIN A4. All sizes a?, b?, c? and d? are European DIN sizes. Letter, legal and executive are US paper formats. All possible values for type <PaperSize> are: letter, legal, executive, a0, a1, a2, a3, a4, a5, a6, a7, a8, a9, b0, b1, b2, b3, b4, b5, b6, b7, b8, b9, c0, c1, c2, c3, c4, c5, c6, c7, c8, c9, d0, d1, d2, d3, d4, d5, d6, d7, d8, and d9. Default value: letter

Content of the report

--include-section-compartment-types[|=]<Boolean> This option decides whether or not a section about compartment types should be included in the resulting model report. Note that this option only causes an effect if the model contains compartment type declarations. Default value: true

--include-section-compartments[|=]<Boolean> This option decides whether or not a section about compartments should be included in the resulting model report. Note that this option only causes an effect if the model contains compartment declarations. Default value: true

--include-section-constraints[|=]<Boolean> This option decides whether or not a section about constraints should be included in the resulting model report. Note that this option only causes an effect if the model contains constraint declarations. Default value: true

--include-section-events[|=]<Boolean> This option decides whether or not a section about events should be included in the resulting model report. Note that this option only causes an effect if the model contains event declarations. Default value: true

--include-section-function-definitions[|=]<Boolean> This option decides whether or not a section about function definitions should be included in the resulting model report. Note that this option only causes an effect if the model declares any function definitions. Default value: true

--include-section-initial-assignments[|=]<Boolean> This option decides whether or not a section about initial assignments should be included in the resulting model report. Note that this option only causes an effect if the model declares any initial assignments. Default value: true

--include-section-parameters[|=]<Boolean> This option decides whether or not a section about parameters should be included in the resulting model report. Note that this option only causes an effect if the model declares any parameters. Default value: true

--include-section-reactions[|=]<Boolean> This option decides whether or not a section about reactions should be included in the resulting model report. Note that this option only causes an effect if the model declares any reactions. Furthermore, this option also decides if a summary of the differential equation system that is implied by the given model should be generated. Again, this will only cause an effect if the model contains any species. Default value: true

--include-section-rules[|=]<Boolean> This option decides whether or not a section about rules should be included in the resulting model report. Note that this option only causes an effect if the model declares any rules, no matter if these are of algebraic, assignment or rate rule type. Default value: true

--include-section-species[|=]<Boolean> This option decides whether or not a section about the species in the given model should be included in the resulting model report. Note that this option only causes an effect if the model declares any species. Default value: `true`

--include-section-species-types[|=]<Boolean> If this option is selected, a section about species types will occur in the model report. Otherwise, this section will be excluded from the report. Default value: `true`

--include-section-unit-definitions[|=]<Boolean> This option decides whether or not a section about the unit definitions of the given model should be included in the resulting model report. Note that this option only causes an effect if the model declares any unit definitions. However, in some level/version combinations SBML models contain predefined unit definitions which might be included in the model report if this option is active. Default value: `true`

Additional options

--include-section-layouts[|=]<Boolean> Include a section with images of model layouts if these are available. Default value: `true`

4.3.9 Garuda options

Garuda is a software framework that allows multiple applications (gadgets) to communicate with each other by sharing data files. In addition, you can launch an application from Garuda, query for tools with specific aims and much more. Since version 2.0, SBMLsqueezer can also be used as a gadget in Garuda.

--connect-to-garuda[|=]<Boolean> Decides whether or not the current application should attempt to connect to the Garuda Core. Default value: `true`

5 Supported rate laws

The kinds of equations supported by the program are numerous, including traditional approaches (Guldberg and Waage, 1879; Michaelis and Menten, 1913) just like very recent equations (Liebermeister and Klipp, 2006; Liebermeister *et al.*, 2010). It provides equations for gene-regulatory processes (Hinze *et al.*, 2007; Radde and Kaderali, 2007; Töpfer *et al.*, 2007; Vu and Vohradský, 2007; Weaver *et al.*, 1999) and approximative rate laws (Savageau, 1969). In addition, SBMLsqueezer covers a large variety of standard rate laws for biochemical reactions from relevant text books (Segel, 1993; Heinrich and Schuster, 1996; Bisswanger, 2000; Cornish-Bowden, 2004). Here, we give a short overview of all equations that are currently implemented in SBMLsqueezer, ordered by the categories metabolic or gene-regulatory. For a detailed description of all rate laws, see the PhD thesis of Dräger, 2011. However, the actual algorithm that suggests applicable rate laws is more complex and considers several features of the reaction. It can therefore happen that SBMLsqueezer suggests multiple different rate equations for the same reaction.

5.1 Rate laws for metabolic processes

- (Generalized) mass-action rate law with numerous orders (Guldberg and Waage, 1879; Heinrich and Schuster, 1996, p. 16), a rate law that has also been shown to be useful as an approximation for more complex mechanisms (Dräger *et al.*, 2007b, 2009a)
- Uni-uni Michaelis-Menten kinetics (Michaelis and Menten, 1913)
- Irreversible non-modulated non-interacting reactant enzymes (see SBO)
- Bi-uni enzyme mechanisms (Segel, 1993; Bisswanger, 2000; Cornish-Bowden, 2004)
  - Random-order mechanism
  - Ordered mechanism
- Bi-bi enzyme reactions (Segel, 1993; Bisswanger, 2000; Cornish-Bowden, 2004)
  - Random-order mechanism (Cornish-Bowden, 2004, p. 169)
  - Ordered mechanism
  - Ping-pong mechanism
- Modular rate laws for enzymatic reactions (Liebermeister *et al.*, 2010)
  - Power-law modular rate law (PM)

  - Common modular rate law (CM)
  - Direct binding modular rate law (DM)
  - Simultaneous binding modular rate law (SM)
  - Force-dependent modular rate law (FM)

- Convenience kinetics (Liebermeister and Klipp, 2006)
  - Thermodynamically dependent form
  - Thermodynamically independent form

- (Generalized) Hill equation (Hill, 1910; Cornish-Bowden, 2004, p. 314)

5.2 Rate laws for gene-regulatory processes

- Hill-Hinze equation (Hinze *et al.*, 2007)

- Hill-Radde equation (Radde and Kaderali, 2007; Radde, 2007)

- Linear additive network models
  - General form
  - Net*Gene*rator form (Töpfer *et al.*, 2007)

- Non-linear additive network models
  - General form
  - Net*Gene*rator form (Töpfer *et al.*, 2007)
  - Vohradský's equation (Vu and Vohradský, 2007)
  - Weaver's equation (Weaver *et al.*, 1999)

- S-systems (Savageau, 1969; Spieth *et al.*, 2004; Tournier, 2005; Spieth *et al.*, 2006; Hecker *et al.*, 2009)

- H-systems (Spieth *et al.*, 2006)

Table 5.1: KEGG IDs of small molecules and ions. This table gives the default list of all small molecules and ions that are ignored by SBMLsqueezer when creating kinetic equations. This list was created according to Blum (2009).

Chemical formula	Common name	KEGG ID
H_2O	Water	C00001
Zn^{2+}	Zinc ion	C00038
Cu^{2+}	Copper ion	C00070
Ca^{2+}	Calcium ion	C00076
H^+	Proton	C00080
Co^{2+}	Cobalt ion	C00175
K^+	Potassium ion	C00238
H_2	Hydrogen	C00282
Ni^{2+}	Nickel ion	C00291
Cl^-	Chloride ion	C00698
HCl	Hydrochloric acid	C01327
H_2Se	Hydrogen selenide	C01528
Fe^{2+}	Iron (II) ion	C14818
Fe^{3+}	Iron (III) ion	C14819

6 FAQ and troubleshooting

Where can I get help for a certain component, option, check-box, etc.?
Most elements in SBMLsqueezer have tool-tips. If you do not understand an option, you can get help in the first place by just pointing the mouse cursor over it and wait for the tool-tip to show up (∼3 seconds).

I'm getting a "java.lang.OutOfMemoryError: Java heap space"
Some operations need a lot of memory. If you simply start SBMLsqueezer, without any Java™ Virtual Machine (JVM) parameters, only 64 MB of memory are available. Please append the argument `-Xmx1024M` to start the application with 1 GB of main memory. See section 4.3.1 on page 40 for a more detailed description of how to start the application with additional memory. If possible, you should give the application 2 GB of main memory. A minimum of 1 GB main memory should be available to the application.

Is an Internet connection required to run SBMLsqueezer?
The vast majority of SBMLsqueezer's functions run in off-line mode. Features that require an active Internet connection are the online check for updates, the extraction of rate laws from the online database SABIO-RK, and following external links within online help.

Where can I obtain the latest version?
Go to `http://www.cogsys.cs.uni-tuebingen.de/software/SBMLsqueezer/`.

Which Java™ version must be installed on my computer to launch SBMLsqueezer?
SBMLsqueezer requires at least Java™ 1.6. Please see `http://www.java.com/de/download/` to download the latest Java™ version.

Why does SBMLsqueezer not start on my Mac with Mac OS X prior to 10.6 Update 3?
If you try to launch SBMLsqueezer, but the application does not start and you receive the following error message on the command-line or Java™ console of your Mac, you need to update your Java™ installation:

```
Exception in thread "AWT-EventQueue-0" java.lang.NoClassDefFoundError:
    com/apple/eawt/AboutHandler
    at java.lang.ClassLoader.defineClass1(Native Method)
    at java.lang.ClassLoader.defineClass(ClassLoader.java:703)
    ...
```

The interface `com.apple.eawt.AboutHandler` was introduced to Java™ for Mac OS X 10.6 Update 3. If you have an earlier version of Mac OS X or Java™, please update your OS or Java™ installation. Also see the Mac OS X documentation about the `AboutHandler` for more information. On a Mac, you can update your Java™ installation through the Software Update menu item in the main Apple menu.

How can I report bugs or get help?
Please contact the mailing list using the e-mail address ✉ `sbmlsqueezer@googlegroups.com`.

7 License

7.1 Disclaimer

SBMLsqueezer is free software: you can redistribute it and/or modify it under the terms of the GNU General Public License (GPL) as published by the Free Software Foundation, either version 3 of the License, or (at your option) any later version.

This program is distributed in the hope that it will be useful, but **without any warranty**; without even the implied warranty of **merchantability** or **fitness for a particular purpose**. See the GNU General Public License for more details.

Each distributed version of SBMLsqueezer should contain a copy of the GNU General Public License. If not, please see `http://www.gnu.org/licenses/`.

7.2 Included third-party libraries

SBMLsqueezer includes and re-distributes the following third-party software libraries.

- JSBML ($1.1\alpha 1$, revision 2354 with CellDesigner module) including all of its third-party libraries, see `http://sbml.org/Software/JSBML`.
- `jtidy-r938.jar`, JTidy License
- SBML2LaTeX (build 20150808-0830, GPL), see `http://sourceforge.net/projects/sbml2latex/` and `http://www.cogsys.cs.uni-tuebingen.de/software/SBML2LaTeX/`.
- libSBML (version 5.9.0, GNU Lesser General Public License (LGPL)), see `http://sbml.org/Software/libSBML`.
- Quaqua filechooser (version 9, LGPL), see `http://www.randelshofer.ch/quaqua/`.
- Garuda (version $1.1\beta 1$) including all of its third-party libraries, see `http://www.garuda-alliance.org`.
- jlatexmath (version 1.0.0, GPL), see `http://forge.scilab.org/index.php/p/jlatexmath/`.

All version numbers are given with respect to the latest release of SBMLsqueezer.

8 Acknowledgments

This work has been funded by a Marie Curie International Outgoing Fellowship awarded to Andreas Dräger within the EU 7th Framework Program for Research and Technological Development (project AMBiCon, 332020) and by the Federal Ministry of Education and Research (BMBF, Germany) in the projects Virtual Liver Network (project number 0315756), National Genome Research Network (NGFN-Plus, project number 01GS08134), and Spher4Sys (grant number 0315384C).

8.1 Core developers

The following people implemented wide parts of SBMLsqueezer:

- Andreas Dräger, University of California, San Diego, La Jolla, California, USA and University of Tuebingen, Germany andraeger@eng.ucsd.edu
- Roland Keller, University of Tuebingen, Germany roland.keller@uni-tuebingen.de
- Johannes Eichner, University of Tuebingen, Germany johannes.eichner@uni-tuebingen.de

8.2 Principal Investigators

- Bernhard O. Palsson, University of California, San Diego, La Jolla, California, USA palsson@ucsd.edu
- Andreas Zell, University of Tuebingen, Germany andreas.zell@uni-tuebingen.de

8.3 Alumni

During the years, many people contributed to this project. We are grateful to each contribution, such as source code, advice, proof reading and much more. In particular, we like to thank our former scientific advisors, colleagues, and students, who are here listed all together in alphabetical order: Meike Aichele, Hannes Borch, Alexander Dörr, Nadine Hassis, Marcel Kronfeld, Oliver Kohlbacher, Sarah Rachel Müller vom Hagen, Sebastian Nagel, Leif J. Pallesen, Alexander Peltzer, Julianus Pfeuffer, Sandra Saliger, Simon Schäfer, Adrian Schröder, Jochen Supper, Dieudonné M. Wouamba, Michael J. Ziller

We also thank Shaowu Yang for providing the Chinese language pack for this program and Michael Römer for maintaining the Galaxy framework and hence the online program version of SBMLsqueezer.

8.4 Collaborators and partners

The authors are grateful to the SABIO-RK team, in particular Martin Golebiewski and Wolfgang Müller from the Heidelberg Institute of Technology (HITS).

Appendix A

Release Notes

This chapter gives a brief overview of the main user-visible changes of SBMLsqueezer since its first release. As such, this chapter gives you an historic overview about the development of SBML-squeezer as a large software project.

A.1 Version 1.0

Release date: April 28th 2008.

This is the initial release of the software that has been made available as a supplement to the original publication from in 2008. All features of this version are therefore described in the corresponding publication Dräger *et al.* (2008).

A.2 Version 1.1

Release date: February 9th 2009.

A.2.1 New features

✔ SBMLsqueezer now allows you to specify whether new parameters should be stored locally (only valid within the respective kinetic law) or globally (valid for the whole model). Both, the main window and the context menu contain switches to make this decision. Please note that the k_G parameters of the thermodynamically independent convenience kinetics are always stored globally because these do not belong to a specific reaction but to a specific reacting species.

✔ The context menu of SBMLsqueezer now also shows already existing kinetic equations and renders their formula. The name of the kinetic equation created by SBMLsqueezer is now written to the notes element of the newly created kinetic law.

✔ The LaTeX export function was improved:

- Several choices allow for a customization of the output.
- The context menu also allows you to export a LaTeX file for a specific kinetic equation.

- It now includes HTML2LaTeX that converts notes elements from XHTML to LaTeX. Therefore, SBMLsqueezer also includes your notes in its model report.

✔ SBMLsqueezer version 1.1 is distributed under the terms of the GNU General Public License (GPL).

Please note that SBMLsqueezer's LaTeX export function is, however, still not comprehensive. For a fully-featured model report generator, we strongly recommend to have a look on our latest project: SBML2LaTeX on `http://webservices.cs.uni-tuebingen.de` and `http://www.cogsys.cs.uni-tuebingen.de/software/SBML2LaTeX`.

A.2.2 Bug fixes

✖ In some cases SBMLsqueezer's context menu did not offer the full list of available and applicable kinetic equations for certain reactions.

✖ If the number of products was higher than the number of reactants a null pointer exception was thrown in the thermodynamically independent form of the convenience kinetics due to an incorrect access to the list of products of a reaction.

✖ In the thermodynamically independent convenience kinetics the root function was called with only one argument. This is actually not incorrect but earlier versions of libSBML are unable to interpret such an implicit square root and require a 2 as the second argument.

✖ In the thermodynamically independent convenience kinetics the exponent of the anabolic term and the catabolic term were incorrect if the stoichiometry was not equal to one. In this case, a division of the exponent by two was missing.

✖ In the mass action kinetics a copy and paste error in the source code lead to incorrect pre-factors for activation and inhibition if both effects were assigned to the same reaction. Instead of receiving one activation function and one inhibition function there was the same activation twice.

✖ For several reasons null pointer exceptions could occur when trying to create LaTeX code from a model.

A.3 Version 1.2

Release date: July 31st 2009.

A.3.1 New features

✔ SBMLsqueezer now automatically checks for updates. If a newer version of SBMLsqueezer is available online, the user is notified by a message window in the bottom right corner of the screen. Furthermore, the release notes of the most recent SBMLsqueezer version are shown to the user by clicking on `show release notes`.

✔ In contrast to previous versions, SBMLsqueezer now indicates if a model already contains rate equations for all reactions or no reactions at all. In earlier versions SBMLsqueezer did not overwrite existing kinetic equations and did also not state why.

✔ SBMLsqueezer's complete internal data structure was improved: It has become much more efficient and simpler.

A.3.2 Improved Adaptation of SBMLsqueezer to CellDesigner 4.0.1

Due to the changes in CellDesigner, SBMLsqueezer only offered zeroth order mass action kinetics as available option to model transcription and translation processes. The reason for this was that the arrows for transcriptional/translational activation disappeared. These effects have now to be covered by trigger and physical stimulation. Unfortunately, CellDesigner maps the old arrows for transcriptional/translational activation to catalysis instead of "trigger". Therefore, we also have to cover the case of having a transcription or translation "catalyzed" by some stimulator. This has now been implemented and SBMLsqueezer therefore offers several kinetic rate equations for both processes. The same holds true for the "batch" mode of SBMLsqueezer: besides transcriptional/-translational activators also all kinds of catalysts and activators/inhibitors are accepted for the Hill equation.

We now apply the inhibition pre-factor from convenience kinetics also to reversible Michaelis-Menten kinetics with multiple inhibitors. In cases where the formulas of Michaelis-Menten and convenience kinetics are equal, only one of both is offered to the user:

- For reversible or irreversible uni-uni reactions without inhibition or activation only the Michaelis-Menten equation is selectable and
- in the case of reversible uni-uni reactions with multiple inhibitors only convenience kinetics can be applied.
- However, if the stoichiometric matrix of the reaction system does not have a full column rank, the thermodynamically independent form of the convenience kinetics can be selected as an alternative to the Michaelis-Menten equation.

Additionally, the activation pre-factor is now also applied for the mixed-type inhibition of irreversible enzymes by mutually exclusive inhibitors (SBO:0000275) if activators are assigned to the reaction.

The new Systems Biology Graphical Notation (SBGN) representation of transcription and translation uses the trigger symbol (for the gene or the RNA molecule, depending on the process) and a reaction from some source to RNA or from some source to a protein (Le Novère *et al.*, 2009). SBMLsqueezer did in these cases not suggest the Hill equation as a possible rate law because it still required the translation/transcription arrows that are going to be deprecated. Since this version, SBMLsqueezer also produces the identical form of the Hill equation for the old style of transcription/translation reaction and the new SBGN-compliant form.

A.3.3 Bug fixes

✖ Local parameters could not be removed completely if not necessary (this happened only in SBMLsqueezer version 1.1 because SBMLsqueezer version 1.0 did not create or delete local parameters at all).

✖ In Michaelis-Menten kinetics activators were multiplied incorrectly (only in the preview, not in the equation itself)

✖ The help browser could not be started in SBMLsqueezer version 1.1 because of a null pointer exception (an image could not be loaded correctly).

✖ Problems of the “batch mode”:

- In some cases SBMLsqueezer did not show the correct SBO numbers (for “Hill equation” and “Henri-Michaelis-Menten equation”).
- In the kinetics summary table SBMLsqueezer did not list the k_G parameters of the thermodynamically independent convenience kinetics in the column “parameters”.
- Sometimes there was a problem with the equation preview.

A.4 Version 1.2.1

Release date: August 7th 2009.

A.4.1 New features

✔ In the context menu, SBMLsqueezer now remembers the rate law when switching the reaction from reversible to irreversible and automatically selects the corresponding equation if available.

A.4.2 Bug fixes

✖ In the Hill equation instead of inhibitors SBMLsqueezer accessed the list of activators to create an inhibition term (only in SBMLsqueezer 1.2).

✖ When trying to remove unnecessary parameters from the model, SBMLsqueezer 1.2 deleted parameters, whose IDs contain upper case letters, no matter if these occur in kinetic equations because of an incorrect String comparison.

✖ The thermodynamically independent convenience kinetics was not created correctly in SBMLsqueezer 1.2: the integer one was subtracted in the denominator in cases where this was incorrect and the parameter k_V was not multiplied with the formula.

A.5 Version 1.3

Release date: April 2nd 2010.

Parts of the content in these release notes have been taken from the Ph.D. thesis of Dräger, 2011, where this section is much more elaborated.

A.5.1 New features

A new and purely Java™-based SBML data structure

SBMLsqueezer's data structure was completely changed: Earlier versions were based on String concatenation to create kinetic formulas with all participating species and parameters. Now SBMLsqueezer is based on an abstract syntax tree representation of the formula and also creates Parameter objects rather than simply their ID.

This new data structure is actually an almost complete Java™ implementation of SBML and has therefore become a separate project, JSBML, available at `http://sourceforge.net/projects/jsbml`. On the SBML homepage, you can find a separate mailing list about this project: `http://sbml.org/Forums`

With JSBML at hand, SBMLsqueezer does no longer manipulate CellDesigner's plug-in Objects directly. If you start SBMLsqueezer from CellDesigner's plug-in menu or its reaction context menu, SBMLsqueezer will copy all required parts of the model into JSBML objects. Only this copy of the model is manipulated. By clicking on [OK] or [Apply], all changes will be synchronized with CellDesigner's original data structures

Stand-Alone Mode

SBMLsqueezer now also runs in a stand-alone mode. With the help of libSBML, valid SBML files can be read and copied into the JSBML data objects. JSBML now mirrors the complete definition of SBML up to Level 2 Version 4. As in the CellDesigner plug-in mode, SBMLsqueezer only manipulates JSBML objects. By clicking on [Save], changes are written into the SBML file.

Command-line mode: All features of SBMLsqueezer are now also available from the command line. This allows users to write shell or batch scripts that access SBMLsqueezer's functions.

The API of SBMLsqueezer has been simplified. There is now just one function, squeeze in the main class SBMLsqueezer that can be called to read in a model from a valid SBML file and to write

the result, i.e., an SBML model including new KineticLaws, new Parameters, new or adapted UnitDefinitions, into the given out file. Before creating kinetic formulas for the model, the user probably wants to adjust SBMLsqueezer's configuration. To this end, the methodsset(CfgKey, <boolean|double|int|String>) in class SBMLsqueezer allow the programmer to set any configuration key and value pair, similar to the command-line mode. Here we give a minimal example of how to use this new API:

```
public static void main(String[] args) {
  // Initialize SBMLsqueezer with appropriate SBML readers/writers
  SBMLsqueezer squeezer = new SBMLsqueezer(new LibSBMLReader(), new
      LibSBMLWriter());
  // Configure SBMLsqueezer
  squeezer.set(CfgKeys.OPT_ALL_REACTIONS_ARE_ENZYME_CATALYZED, true);
  squeezer.set(CfgKeys.OPT_DEFAULT_COMPARTMENT_INITIAL_SIZE, 1.0);
  squeezer.set(CfgKeys.POSSIBLE_ENZYME_RNA, true);
  squeezer.set(CfgKeys.KINETICS_UNI_UNI_TYPE,
      MichaelisMenten.class.getName());
  squeezer.set(CfgKeys.KINETICS_OTHER_ENZYME_REACTIONS,
      ConvenienceKinetics.class.getName());
  try {
    // Create kinetic equations, parameters, units etc. and save the result
    // args contains paths to input file and output file
    squeezer.squeeze(args[0], args[1]);
  } catch (Throwable e) {
    e.printStackTrace();
  }
}
```

Listing A.1: Usage of SBMLsqueezer 1.3 via its API

Since SBMLsqueezer still requires an installation of libSBML on the user's system, which can sometimes become problematic, a more convenient way of using SBMLsqueezer has now been made available: the SBMLsqueezer web application does not require any local installation and is freely available at http://webservices.cs.uni-tuebingen.de. In this framework, workflows can be created in which SBMLsqueezer can directly be linked to the full version of SBML2LaTeX. The full version of SBML2LaTeX contains many additional features that are not included in SBMLsqueezer's version of SBML2LaTeX. Hence, it is highly recommended to make use of the online version of SBML2LaTeX.

Configuration

SBMLsqueezer now memorizes every setting and changed parameters from the GUI whether in CellDesigner plug-in or stand-alone mode. In the stand-alone case, SBMLsqueezer even memorizes given command-line arguments. These settings are read from a configuration file at the next start. This is especially convenient for open and save directories. At any time, the user can change all settings to the defaults by clicking on the designated button in the preferences dialog.

Improved Systems Biology Ontology support

SBMLsqueezer is now fully based on the Systems Biology Ontology (SBO). The JSBML copy of CellDesigner's plug-in data structures maps all CellDesigner-specific annotations to corresponding SBO terms. Afterwards these are mapped back. SBO term "enzymatic catalyst" (SBO:0000460) was created because it is required by SBMLsqueezer to distinguish enzymes and other catalysts, such as certain inorganic ions. SBMLsqueezer's stand-alone version interprets SBO terms that can be found in the model and changes the annotation of ModifierSpeciesReferences from "catalyst" (SBO:0000013) to "enzymatic catalyst" if the SBO term of the corresponding Species is one of those belonging to the family of materials considered as enzymes (SBMLsqueezer still offers check boxes to indicate certain kinds of species as enzymes).

Earlier versions of SBMLsqueezer indicated the SBO term IDs of newly created kinetic equations if these were already defined in SBO. However, this was more or less just for information to the user and could neither be saved nor be considered during any other processes. This version of SBMLsqueezer is able to not only take SBO terms into account while creating rate laws, but can even save SBO terms in the resulting SBML file. However, in CellDesigner plug-in mode, SBMLsqueezer is not yet able to save SBO term IDs in CellDesigner data objects due to the missing functionality in its API.

SBMLsqueezer now contains an own parser to identify the correct SBO term IDs and attributes that are required to annotate all newly created objects.

Whenever possible, SBMLsqueezer annotates newly created objects, such as `Parameters` or `UnitDefinitions`, with SBO IDs.

MIRIAM Support

In contrast to earlier versions, SBMLsqueezer now also interprets and uses MIRIAM tags. The new command-line and configuration key option `OPT_IGNORE_THESE_SPECIES_WHEN_CREATING_LAWS` takes a list of comma-separated MIRIAM IDs as value. Species annotated with `BQB_IS` and the given MIRIAM resource are then ignored when creating rate equations. This is, for instance, useful if small molecules such as water take part in a reaction. The concentration of water can hardly be measured and it is assumed to occur abundantly within the cell. By default, the list of species whose influence is to be neglected when creating new kinetic equations contains the KEGG compound IDs for the substances listed in table 5.1 on page 61.

New kinetic equations and extensibility

In earlier versions the inclusion of new kinetic equations into SBMLsqueezer was not an easy task. After writing a class that actually generates the new rate law, a rule had to be implemented of when to make this rate law available. Furthermore, the GUI had to be changed to include this new formula. Summarized, several changes in the program code were necessary to include additional rate laws.

Now SBMLsqueezer is based on Java™ reflection, i.e., initially SBMLsqueezer does not know any one of its available kinetic equations. These are all loaded when initializing SBMLsqueezer. Currently, twelve interfaces define the properties of all kinetic equations. The rules of when select which kind of rate equation only make use of the implemented interfaces. Hence, new kinetic equations can be incorporated into SBMLsqueezer simply by implementing designated interfaces. Since SBMLsqueezer is an open-source project, users can customize it easily and extend it with desired rate laws.

Much effort was put into the extension of SBMLsqueezer with additional rate laws for gene-regulatory processes. Earlier versions of SBMLsqueezer only provided the Hill equation variant suggested by Hinze *et al.* (2007). This equation has now been renamed to Hill-Hinze equation to highlight that it is actually a modification of the original rate law. Only in special cases this equation equals the traditional Hill equation (SBMLsqueezer will highlight these cases). Now nine additional rate equations were included:

Additive Model Linear A generalized super class of all other additive rate laws for gene-regulatory processes.

Additive Model Non-Linear defined in the paper "The Net*Gene*rator Algorithm] Reconstruction of Gene Regulatory Networks" by Töpfer, Guthke, Driesch, Wötzel, and Pfaff, 2007

H-Systems Spieth, Hassis, Streichert, Supper, Beyreuther, and Zell, 2006 "Comparing Mathematical Models on the Problem of Network Inference".

NetGenerator Linear Model "The Net*Gene*rator Algorithm: Reconstruction of Gene Regulatory Networks" by Töpfer, Guthke, Driesch, Wötzel, and Pfaff, 2007

NetGenerator Non-Linear Model "The Net*Gene*rator Algorithm] Reconstruction of Gene Regulatory Networks" by Töpfer, Guthke, Driesch, Wötzel, and Pfaff, 2007

S-Systems Tournier (2005): "Approximation of dynamical systems using S-systems theory: application to biological systems"

Hill-Radde equation "Modeling Non-Linear Dynamic Phenomena in Biochemical Networks" by Radde, 2007

Vohradsky "Neural network model of gene expression." of Vu and Vohradský, 2007

Weaver "Modeling regulatory networks with weight matrices" by Weaver, Workman, and Stormo, 1999

The following new generalized kinetic equations for metabolic reactions were also implemented in SBMLsqueezer, which are all defined by Liebermeister *et al.* (2010):

- Common modular rate law (CM)

- Direct binding modular rate law (DM)
- Force-dependent modular rate law (FM)
- Power-law modular rate law (PM)
- Simultaneous binding modular rate law (SM).

Each one of these equations is only available in a reversible mode and the user must choose one of the three possible versions that determine the degree of thermodynamic correctness. The *weg* version is the most complicated form but always thermodynamically correct. The *hal* version ensures thermodynamic correctness in most cases, whereas for the *cat* version thermodynamic properties cannot be guaranteed to be correct. The versions can be selected in the preferences dialog or provides as a command-line option.

Finally, the generalized form of Hill's equation as proposed by Cornish-Bowden (2004, in "Fundamentals of Enzyme Kinetics", p. 314) has been implemented and is now also available for metabolic reactions.

If the corresponding flag is set, boundary conditions are also set for species that represent empty sets, i.e., degraded species, sources, or sinks in a reaction.

Support and assignment of units and unit definitions

For multi-compartment models concentration versus molecule counts matters. SBMLsqueezer now provides two different ways of ensuring unit correctness. First, each species can be brought to units of concentration. To this end, SBMLsqueezer interprets the `hasOnlySubstanceUnits` attribute of each species. Depending on whether this is set to true, the species is divided by the size of the surrounding compartment. Otherwise the species already represents a concentration and nothing is done. Second, all species can be brought to molecule count units. This is again achieved with the help of the `hasOnlySubstanceUnits` attribute, but now SBMLsqueezer multiplies with the compartment size if this attribute is false.

In contrast to earlier versions, SBMLsqueezer now equips all newly created Parameter objects with units. To this end, SBMLsqueezer derives the correct units from the context. For instance, in the mass action kinetics the units of the catalytic constants depend on the order of the reaction and have therefore to be computed whenever such a rate law is created. Furthermore, SBMLsqueezer also to considers the `hasOnlySubstanceUnits` property of each species and the way in which the surrounding compartment comes into play to derive the units of parameters.

To make models more realistic, SBMLsqueezer redefines the pre-defined SBML unit definition substance from mol to mmol and volume from l to ml.

If possible, SBMLsqueezer avoids creating new `UnitDefinition` objects. It first tries to find equivalent and already existing definitions in the model.

A.5.2 Bug Fixes

- ✖ In the original paper of Hinze *et al.* (2007), inhibition was expressed with 1 - product of all inhibition functions. Earlier versions of SBMLsqueezer created a product that run over all inhibitors with (1 - inhibition function) for each such function.

- ✖ In all gene-regulation kinetics, it is now assumed that degradation processes are distinct reactions that can be, e.g., modeled using a mass action rate law. Hence, no degradation terms can be found in gene-regulation kinetics anymore.

- ✖ The rate law corresponding to `SBO:0000266` was not correct. Earlier versions of SBML-squeezer produced the fraction $K_{\mathrm{M}js}/K_{\mathrm{ib}j} \cdot I$, but correct is $S \cdot {}^{I}/_{K_{\mathrm{ib}j}}$ (see Equation 10 in the supplementary material “Kinetic Laws”; the correct version of this equation can be found at `http://www.ebi.ac.uk/sbo/main/SBO:0000266`).

- ✖ In case of an explicit enzyme catalysis, earlier versions of SBMLsqueezer did not multiply the numerator of equation SBO:0000273 with the enzyme. Therefore, it always produced the $V_{\max}$ version of the equation, but the parameter was called k_{cat}.

A.6 Version 2.0

Release date: June 30th 2014.

Many concepts that have been introduced with version 1.3 have been revised and updated. The entire handling of user settings has been massively changed and now uses the concept of user preferences instead of configuration files. The source code has been cleaned and re-factored in many positions and lots of development time has been invested into with the library JSBML.

A.6.1 New features

SBMLsqueezer can now be used in several different ways:

- As fully-featured stand-alone program without the need to install any further software, based on the official JSBML version 1.0β1

- As a stand-alone program based on a libSBML back-end, which means that real off-line SBML validation is possible.

- Irrespective of whether JSBML or libSBML is used as SBML library, the stand-alone version provides both, a GUI as well as a fully-featured command-line version. For large-scale rate law generation, this command-line interface has already proven to be useful (as part of the path2models project).

- SBMLsqueezer can be launched directly from the project’s website as a Java™ Web Start program without the need of any local installation.

- As a plug-in for CellDesigner 4.3
- As a Garuda gadget, as which it can communicate with further Garuda gadgets

Hence, the usage of the program has been greatly simplified. Further new program features comprise:

- ✔ SBMLsqueezer fully supports all Levels and Versions of SBML up to the most recent specification Level 3 Version 1.
- ✔ A direct query wizard for SABIO-RK has been implemented and allows users to extract experimentally obtained rate laws from this database together with parameters, values, and units. The search engine provides several settings for experimental conditions.
- ✔ SBMLsqueezer includes now several JUnit test cases to check if the generated kinetic equations are correct.
- ✔ It includes now a full version of SBML2LaTeX and can directly open Portable Document Format (PDF) files that are generated to document the content of the model.
- ✔ The renderer for equations and formula has been changed and provides more capabilities. SBMLsqueezer windows and context menus are now zoom-able and re-sizable.
- ✔ Improved user preferences menu and management for each individual operating system.
- ✔ The user interface (GUI and command-line) is fully bilingual (German and English), and partially also translated to Chinese, but models and their labels are only created in English for the sake of a better international re-usability.
- ✔ A new search function allows users to query the model data structure in the GUI.
- ✔ Lots of effort has been put to better adapt SBMLsqueezer to individual platforms. The support for Mac OS X has been greatly improved, the program now provides many features of native applications for Mac OS X.
- ✔ The documentation of the API has been improved.

A.6.2 Bug fixes

The communication between SBMLsqueezer and CellDesigner was very inefficient. The new plug-in interface in JSBML provides a much better performance. Errors in kinetic equations and units of parameters have been detected and solved:

- ✖ Sometimes SBMLsqueezer created invalid IDs for newly introduced units.

- ✖ The units of the association and dissociation constant in generalized mass action kinetics could not be derived correctly if a catalyst interfered with the reaction, because the units of the catalyst were not taken into account.

- ✖ The units of the half saturation constant in the (generalized) Hill equation were not correctly derived. The derivation of units of kinetic parameters has been improved.

Several minor issues with the GUI have been solved.

A.6.3 Known Issues

In SBML models, it should be possible to evaluate all kinetic equations to extend of reaction units per time units. Usually, the extend of a reaction would be a variant of a substance unit. However, it has been recognized that some kinetic equations cannot under all conditions be evaluated to extend per time units. The reasons are often the structure of the definition of the rate law. Some equations that have been defined for gene-regulatory networks, for instance, give a purely phenomenological description of the system (e.g., S- or H-systems). In other equations, it is assumed that the equilibrium constant is always dimensionless. If this is not the case, it cannot be guaranteed that SBMLsqueezer will create correct units for these equations (e.g., modular rate laws and convenience kinetics). It is therefore recommended to check the units of generated rate laws, even if in the vast majority of cases these will be correct. Please note that in this version the general unit handling has been extensively revised and improved and is in the vast majority of cases very reliable. SBMLsqueezer displays the derived units of equations, so that problematic equations can be easily identified.

A.7 Version 2.0.1

Release date: October 24th 2014.

This release has become necessary due to major changes in the Garuda backend and also includes an updated version of JSBML.

A.8 Version 2.1

Release date: October 10th 2015.

A.8.1 New Features

- ✔ SBMLsqueezer fully supports all Levels and Versions of SBML up to the most recent specifications Level 2 Version 5 and Level 3 Version 1.

- ✔ The CellDesigner plug-in version has been adapted to work with version 4.4.

- ✔ The Garuda backend has been updated.
- ✔ The report generator SBML2LaTeX has been updated and the new version is now part of this release of SBMLsqueezer.
- ✔ SBMLsqueezer comes with the most recent version of JSBML (revision 2354), which can read all current extension packages.
- ✔ Slightly improved API documentation and updated Users' Guide.
- ✔ Correctly indented SBML output files.

A.8.2 Bug Fixes

Under Microsoft Windows, it was sometimes not possible to correctly display user messages. This has been fixed. Several minor issues have been solved.

Appendix B

Acronyms

API Application Programming Interface

BioPAX Biological Pathway Exchange Language

FAQ Frequently Asked Questions

GPL GNU General Public License

GUI Graphical User Interface

HTML Hypertext Markup Language

ID Identifier

IDE Integrated Development Environment

JDK Java™ Development Kit

JVM Java™ Virtual Machine

JAR Java™ Archive

LGPL GNU Lesser General Public License

KEGG Kyoto Encyclopedia of Genes and Genomes

MIRIAM Minimal Information Required In the Annotation of Models

PDF Portable Document Format

RNA Ribonucleic Acid

SBGN Systems Biology Graphical Notation

SBML Systems Biology Markup Language

SABIO-RK System for the Analysis of Biochemical Pathways – Reaction Kinetics

SBO Systems Biology Ontology

XHTML Extended HTML

XML Extended Markup Language

Bibliography

Arnold, A. and Nikoloski, Z. (2011). A quantitative comparison of calvin-benson cycle models. *Trends Plant Sci*, **16**(12), 676–683.

Bisswanger, H. (2000). *Enzymkinetik – Theorie und Methoden*. Wiley-VCH, Weinheim, Germany, 3rd edition.

Blum, T. (2009). *Computational Approaches for Analyzing Metabolic Pathways*. Ph.D. thesis, Eberhard-Karls-Universität Tübingen, Tübingen, Germany.

Bornstein, B. J., Keating, S. M., Jouraku, A., and Hucka, M. (2008). LibSBML: an API Library for SBML. *Bioinformatics*, **24**(6), 880–881.

Büchel, F., Wrzodek, C., Mittag, F., Dräger, A., Eichner, J., Rodriguez, N., Le Novère, N., and Zell, A. (2012). Qualitative translation of relations from BioPAX to SBML qual. *Bioinformatics*, **28**(20), 2648–2653.

Büchel, F., Rodriguez, N., Swainston, N., Wrzodek, C., Czauderna, T., Keller, R., Mittag, F., Schubert, M., Glont, M., Golebiewski, M., van Iersel, M., Keating, S., Rall, M., Wybrow, M., Hermjakob, H., Hucka, M., Kell, D. B., Müller, W., Mendes, P., Zell, A., Chaouiya, C., Saez-Rodriguez, J., Schreiber, F., Laibe, C., Dräger, A., and Le Novère, N. (2013). Large-scale generation of computational models from biochemical pathway maps. *BMC Systems Biology*, **7**(1), 116.

Buswell, S., Devitt, S., Diaz, A., Ion, P., Miner, R., Poppelier, N., Smith, B., Soiffer, N., Sutor, R., and Watt, S. (1999). Mathematical markup language (mathml™) 1.01 specification. Technical report, MIT, INRIA, Keio.

Cornish-Bowden, A. (2004). *Fundamentals of Enzyme Kinetics*. Portland Press Ltd., 59 Portland Place, London, United Kingdom, 3rd edition.

Courtot, M., Juty, N., Knüpfer, C., Waltemath, D., Zhukova, A., Dräger, A., Dumontier, M., Finney, A., Golebiewski, M., Hastings, J., Hoops, S., Keating, S., Kell, D. B., Kerrien, S., Lawson, J., Lister, A., Lu, J., Machne, R., Mendes, P., Pocock, M., Rodriguez, N., Villéger, A., Wilkinson, D. J., Wimalaratne, S., Laibe, C., Hucka, M., and Le Novère, N. (2011). Controlled vocabularies and semantics in systems biology. *Mol Syst Biol*, **7**, 543.

Dörr, A., Keller, R., Zell, A., and Dräger, A. (2014). SBMLsimulator: a Java tool for model simulation and parameter estimation in systems biology. *Computational Biology*.

Dräger, A. (2011). *Computational Modeling of Biochemical Networks*. Ph.D. thesis, University of Tuebingen, Tübingen, Germany.

Dräger, A. and Palsson, B. Ø. (2014). Improving collaboration by standardization efforts in systems biology. *Frontiers in Bioengineering*, **2**(61).

Dräger, A., Kronfeld, M., Supper, J., Planatscher, H., Magnus, J. B., Oldiges, M., and Zell, A. (2007a). Benchmarking Evolutionary Algorithms on Convenience Kinetics Models of the Valine and Leucine Biosynthesis in *C. glutamicum*. In D. Srinivasan and L. Wang, editors, *2007 IEEE Congress on Evolutionary Computation*, pages 896–903, Singapore. IEEE Computational Intelligence Society, IEEE Press.

Dräger, A., Supper, J., Planatscher, H., Magnus, J. B., Oldiges, M., and Zell, A. (2007b). Comparing Various Evolutionary Algorithms on the Parameter Optimization of the Valine and Leucine Biosynthesis in *Corynebacterium glutamicum*. In D. Srinivasan and L. Wang, editors, *2007 IEEE Congress on Evolutionary Computation*, pages 620–627, Singapore. IEEE Computational Intelligence Society, IEEE Press.

Dräger, A., Hassis, N., Supper, J., Schröder, A., and Zell, A. (2008). SBMLsqueezer: a CellDesigner plug-in to generate kinetic rate equations for biochemical networks. *BMC Systems Biology*, **2**(1), 39.

Dräger, A., Kronfeld, M., Ziller, M. J., Supper, J., Planatscher, H., Magnus, J. B., Oldiges, M., Kohlbacher, O., and Zell, A. (2009a). Modeling metabolic networks in *C. glutamicum*: a comparison of rate laws in combination with various parameter optimization strategies. *BMC Syst Biol*, **3**, 5.

Dräger, A., Planatscher, H., Wouamba, D. M., Schröder, A., Hucka, M., Endler, L., Golebiewski, M., Müller, W., and Zell, A. (2009b). SBML2LaTeX: Conversion of SBML files into human-readable reports. *Bioinformatics*, **25**(11), 1455–1456.

Dräger, A., Schröder, A., and Zell, A. (2010). *Systems Biology for Signaling Networks*, volume 1 of *Systems Biology*, chapter Automating mathematical modeling of biochemical reaction networks, pages 159–205. Springer-Verlag.

Dräger, A., Rodriguez, N., Dumousseau, M., Dörr, A., Wrzodek, C., Le Novère, N., Zell, A., and Hucka, M. (2011). JSBML: a flexible Java library for working with SBML. *Bioinformatics*, **27**(15), 2167–2168.

Finney, A. and Hucka, M. (2003). Systems biology markup language: Level 2 and beyond. *Biochem Soc Trans*, **31**(Pt 6), 1472–1473.

Finney, A., Hucka, M., and Le Novére, N. (2006). Systems Biology Markup Language (SBML) Level 2: Structures and Facilities for Model Definitions. Technical report.

Funahashi, A., Tanimura, N., Morohashi, M., and Kitano, H. (2003). CellDesigner: a process diagram editor for gene-regulatory and biochemical networks. *BioSilico*, **1**(5), 159–162.

Funahashi, A., Matsuoka, Y., Jouraku, A., Kitano, H., and Kikuchi, N. (2006). CellDesigner: a modeling tool for biochemical networks. In *Proceedings of the 38th conference on Winter simulation*, WSC '06, pages 1707–1712, Monterey, California. Winter Simulation Conference.

Funahashi, A., Morohashi, M., Matsuoka, Y., Jouraku, A., and Kitano, H. (2007). CellDesigner: A Graphical Biological Network Editor and Workbench Interfacing Simulator. In S. Choi, editor, *Introduction to Systems Biology*, chapter 21, pages 422–434. Humana Press.

Funahashi, A., Matsuoka, Y., Jouraku, A., Morohashi, M., Kikuchi, N., and Kitano, H. (2008). CellDesigner 3.5: A Versatile Modeling Tool for Biochemical Networks. In *Proceedings of the IEEE*, volume 96, pages 1254–1265. IEEE.

Gauges, R., Rost, U., Sahle, S., and Wegner, K. (2006). A model diagram layout extension for SBML. *Bioinformatics*, **22**(15), 1879–1885.

Ghosh, S., Matsuoka, Y., Asai, Y., Hsin, K.-Y., and Kitano, H. (2011). Software for systems biology: from tools to integrated platforms. *Nature Reviews Genetics*, **12**(12), 821–832.

Goecks, J., Nekrutenko, A., Taylor, J., and Team, G. (2010). Galaxy: a comprehensive approach for supporting accessible, reproducible, and transparent computational research in the life sciences. *Genome Biology*, **11**(8), R86.

Guldberg, C. M. and Waage, P. (1879). Über die chemische affinität. *Journal für Practische Chemie*, **127**, 69–114.

Hecker, M., Lambeck, S., Töpfer, S., Someren, E. v., and Guthke, R. (2009). Gene regulatory network inference: data integration in dynamic models-a review. *Biosystems*, **96**(1), 86–103.

Heinrich, R. and Schuster, S. (1996). *The Regulation of Cellular Systems*. Chapman and Hall, New York, NY, USA.

Hill, A. V. (1910). The possible effects of the aggregation of the molecules of haemoglobin on its dissociation curves. *Journal of Physiology*, **40**(4), iv–vii.

Hinze, T., Hayat, S., Lenser, T., Matsumaru, N., and Dittrich, P. (2007). Hill Kinetics meets P Systems: A Case Study on Gene Regulatory Networks as Computing Agents *in silico* and *in vivo*. In G. Eleftherakis, P. Kefalas, and G. Paun, editors, *Proceedings of the Eight Workshop on Membrane Computing*, pages 363–381. SEERC.

Hucka, M., Finney, A., Sauro, H., and Bolouri, H. (2001). Systems Biology Markup Language (SBML) Level 1: Structures and Facilities for Basic Model Definitions. Technical report, Systems Biology Workbench Development Group ERATO Kitano Systems Biology Project Control and Dynamical Systems, MC 107-81.

Hucka, M., Finney, A., Sauro, H., and Bolouri, H. (2003a). Systems Biology Markup Language (SBML) Level 1: Structures and Facilities for Basic Model Definitions. Technical Report 2, Systems Biology Workbench Development Group JST ERATO Kitano Symbiotic Systems Project Control and Dynamical Systems, MC 107-81, California Institute of Technology, Pasadena, CA, USA.

Hucka, M., Finney, A., Sauro, H. M., Bolouri, H., Doyle, J. C., Kitano, H., Arkin, A. P., Bornstein, B. J., Bray, D., Cornish-Bowden, A., Cuellar, A. A., Dronov, S., Gilles, E. D., Ginkel, M., Gor, V., Goryanin, I. I., Hedley, W. J., Hodgman, T. C., Hofmeyr, J.-H. S., Hunter, P. J., Juty, N. S., Kasberger, J. L., Kremling, A., Kummer, U., Le Novère, N., Loew, L. M., Lucio, D., Mendes, P., Minch, E., Mjolsness, E. D., Nakayama, Y., Nelson, M. R., Nielsen, P. F., Sakurada, T., Schaff, J. C., Shapiro, B. E., Shimizu, T. S., Spence, H. D., Stelling, J., Takahashi, K., Tomita, M., Wagner, J. M., Wang, J., and the rest of the SBML Forum (2003b). The systems biology markup language (SBML): a medium for representation and exchange of biochemical network models. *Bioinformatics*, **19**(4), 524–531.

Hucka, M., Finney, A. M., Hoops, S., Keating, S. M., and Le Novère, N. (2007). Systems Biology Markup Language (SBML) Level 2: Structures and Facilities for Model Definitions. Technical Report 1.

Hucka, M., Finney, A., Hoops, S., Keating, S. M., and Le Novère, N. (2008). Systems biology markup language (SBML) Level 2: structures and facilities for model definitions. Technical report, Nature Precedings.

Hucka, M., Bergmann, F. T., Hoops, S., Keating, S. M., Sahle, S., Schaff, J. C., Smith, L., and Wilkinson, D. J. (2010). The Systems Biology Markup Language (SBML): Language Specification for Level 3 Version 1 Core. Technical report, Nature Precedings.

Hucka, M., Bergmann, F. T., Dräger, A., Hoops, S., Keating, S. M., Le Novère, N., Myers, C. J., Olivier, B. G., Sahle, S., Schaff, J. C., Smith, L. P., Waltemath, D., and Wilkinson, D. J. (2015). Systems Biology Markup Language (SBML) Level 2: Structures and Facilities for Model Definitions. Technical report.

Juty, N., Le Novère, N., and Laibe, C. (2012). Identifiers. org and MIRIAM Registry: community resources to provide persistent identification. *Nucleic Acids Research*, **40**(D1), D580–D586.

Kanehisa, M. and Goto, S. (2000). KEGG: Kyoto Encyclopedia of Genes and Genomes. *Nucleic Acids Research*, **28**(1), 27–30.

Keller, R., Dörr, A., Tabira, A., Funahashi, A., Ziller, M. J., Adams, R., Rodriguez, N., Le Novère, N., Hiroi, N., Planatscher, H., Zell, A., and Dräger, A. (2013). The systems biology simulation core algorithm. *BMC Syst Biol*.

Krebs, O., Golebiewski, M., Kania, R., Mir, S., Saric, J., Weidemann, A., Wittig, U., and Rojas, I. (2007). SABIO-RK: A data warehouse for biochemical reactions and their kinetics. *Journal of Integrative Bioinformatics*, **4**(1).

Laible, C. and Le Novère, N. (2007). MIRIAM Resources: tools to generate and resolve robust cross-references in Systems Biology. *BMC Systems Biology*, **13**(58), 58–67.

Le Novère, N., Finney, A., Hucka, M., Bhalla, U. S., Campagne, F., Collado-Vides, J., Crampin, E. J., Halstead, M., Klipp, E., Mendes, P., Nielsen, P., Sauro, H., Shapiro, B. E., Snoep, J. L., Spence, H. D., and Wanner, B. L. (2005). Minimum information requested in the annotation of biochemical models (MIRIAM). *Nature Biotechnology*, **23**(12), 1509–1515.

Le Novère, N., Courtot, M., and Laibe, C. (2006). Adding semantics in kinetics models of biochemical pathways. In C. Kettner and M. G. Hicks, editors, *2nd International ESCEC Workshop on Experimental Standard Conditions on Enzyme Characterizations. Beilstein Institut, Rüdesheim, Germany*, pages 137–153, Rüdessheim/Rhein, Germany. ESEC.

Le Novère, N., Hucka, M., Mi, H., Moodie, S., Schreiber, F., Sorokin, A., Demir, E., Wegner, K., Aladjem, M. I., Wimalaratne, S. M., Bergmann, F. T., Gauges, R., Ghazal, P., Kawaji, H., Li, L. M., Lu, Y., Villéger, A., Boyd, S. E., Calzone, L., Courtot, M., Dogrusoz, U., Freemann, T. C., Funahashi, A., Ghosh, S., Jouraku, A., Sohyoung, K., Kolpakov, F., Luna, A., Sahle, S., Schmidt, E., Watterson, S., Wu, G., Goryanin, I., Kell, D. B., Sander, C., Sauro, H., Snoep, J. L., Kohn, K., and Kitano, H. (2009). The Systems Biology Graphical Notation. *Nature biotechnology*, **27**(8), 735–741.

Li, C., Donizelli, M., Rodriguez, N., Dharuri, H., Endler, L., Chelliah, V., Li, L., He, E., Henry, A., Stefan, M. I., Snoep, J. L., Hucka, M., Le Novère, N., and Laibe, C. (2010). Biomodels database: An enhanced, curated and annotated resource for published quantitative kinetic models. *BMC Syst Biol*, **4**, 92.

Liebermeister, W. and Klipp, E. (2006). Bringing metabolic networks to life: convenience rate law and thermodynamic constraints. *Theor Biol Med Model*, **3**(42), 41.

Liebermeister, W., Uhlendorf, J., and Klipp, E. (2010). Modular rate laws for enzymatic reactions: thermodynamics, sensitivities, and implementation. *submitted to Bioinformatics*.

Michaelis, L. and Menten, M. L. (1913). Kinetik der Invertinwirkung. *Biochemische Zeitschrift*, **49**, 333–369.

Radde, N. (2007). *Modeling Non-Linear Dynamic Phenomena in Biochemical Networks*. Ph.D. thesis, Faculty of Mathematics and Natural Sciences, University of Cologne, Germany.

Radde, N. and Kaderali, L. (2007). *Bayesian Inference of Gene Regulatory Networks Using Gene Expression Time Series Data*, volume 4414/2007, chapter 1, pages 1–15. Springer Berlin/Heidelberg.

Rodriguez, N., Thomas, A., Watanabe, L., Vazirabad, I. Y., Kofia, V., Gómez, H. F., Mittag, F., Matthes, J., Rudolph, J., Wrzodek, F., Netz, E., Diamantikos, A., Eichner, J., Keller, R., Wrzodek, C., Fröhlich, S., Lewis, N. E., Myers, C. J., Le Novère, N., Palsson, B. Ø., Hucka, M., and Dräger, A. (2015). JSBML 1.0: providing a smorgasbord of options to encode systems biology models. *Bioinformatics*.

Rojas, I., Golebiewski, M., Kania, R., Krebs, O., Mir, S., Weidemann, A., and Wittig, U. (2007). Storing and Annotating of Kinetic Data. *In Silico Biology*, **7**, 37–44.

Savageau, M. A. (1969). Biochemical systems analysis. I. Some mathematical properties of the rate law for the component enzymatic reactions. *J Theor Biol*, **25**(3), 365–369.

Segel, I. H. (1993). *Enzyme Kinetics—Behavior and Analysis of Rapid Equilibrium and Steady-State Enzyme Systems*. Wiley-Intersciennce, New York, NY, USA.

Spieth, C., Streichert, F., Speer, N., and Zell, A. (2004). Optimizing Topology and Parameters of Gene Regulatory Network Models from Time-Series Experiments. In *Proceedings of the Genetic and Evolutionary Computation Conference (GECCO 2004)*, volume 3102 (Part I) of *LNCS*, pages 461–470.

Spieth, C., Hassis, N., Streichert, F., Supper, J., Beyreuther, K., and Zell, A. (2006). Comparing Mathematical Models on the Problem of Network Inference. In *Proceedings of the 8th annual conference on Genetic and evolutionary computation*, GECCO '06, pages 279–286, New York, NY, USA. ACM.

Töpfer, S., Guthke, R., Driesch, D., Wötzel, D., and Pfaff, M. (2007). The Net*Gene*rator Algorithm: reconstruction of gene regulatory networks. In K. Tuyls, R. Westra, Y. Saeys, and A. Nowé, editors, *Knowledge Discovery and Emergent Complexity in Bioinformatics*, volume 4366 of *Lecture Notes in Computer Science*.

Tournier, L. (2005). Approximation of dynamical systems using s-systems theory: application to biological systems. In *ISSAC '05: Proceedings of the 2005 international symposium on Symbolic and algebraic computation*, pages 317–324, New York, NY, USA. ACM press.

Vu, T. T. and Vohradský, J. (2007). Nonlinear differential equation model for quantification of transcriptional regulation applied to microarray data of saccharomyces cerevisiae. *Nucleic Acids Res*, **35**(1), 279–287.

Weaver, D. C., Workman, C. T., and Stormo, G. D. (1999). Modeling regulatory networks with weight matrices. In *Pacific Symposium on Biocomputing*, volume 4, pages 112–123. World Scientific Publishing.

Wittig, U., Golebiewski, M., Kania, R., Krebs, O., Mir, S., Weidemann, A., Anstein, S., Saric, J., and Rojas, I. (2006). SABIO-RK: Integration and Curation of Reaction Kinetics Data. In

U. Leser, F. Naumann, and B. Eckmann, editors, *Data Integration in the Life Sciences*, pages 94–103. Springer Berlin/Heidelberg.

Wittig, U., Kania, R., Golebiewski, M., Rey, M., Shi, L., Jong, L., Algaa, E., Weidemann, A., Sauer-Danzwith, H., Mir, S., Krebs, O., Bittkowski, M., Wetsch, E., Rojas, I., and Müller, W. (2012). SABIO-RK–database for biochemical reaction kinetics. *Nucleic Acids Res*, **40**(Database issue), D790–D796.

Wolf, J., Passarge, J., Somsen, O. J. G., Snoep, J. L., Heinrich, R., and Westerhoff, H. V. (2000). Transduction of intracellular and intercellular dynamics in yeast glycolytic oscillations. *Biophys J*, **78**(3), 1145–1153.

Wrzodek, C., Dräger, A., and Zell, A. (2011). KEGGtranslator: visualizing and converting the KEGG PATHWAY database to various formats. *Bioinformatics*, **27**(16), 2314–2315.

Wrzodek, C., Büchel, F., Ruff, M., Dräger, A., and Zell, A. (2013). Precise generation of systems biology models from kegg pathways. *BMC Syst Biol*, **7**(1), 15.

Index

Symbols

Application Programming Interface (API) . . 3, 5, 10, 20, 33–36, 38, 39, 71–73, 77, 79
Biological Pathway Exchange Language (BioPAX) 2
GNU General Public License (GPL) 64
Graphical User Interface (GUI) 3–5, 10, 13, 20, 24, 31, 37, 39–41, 53, 72, 76–78
Integrated Development Environment (IDE) . . 35
Java™ Archive (JAR) 5–8, 40
Java™ Development Kit (JDK) 6
Java™ Virtual Machine (JVM) . . . 5, 6, 8, 41, 62
Kyoto Encyclopedia of Genes and Genomes (KEGG) 23, 29, 38, 43, 61, 73
GNU Lesser General Public License (LGPL) 64
Minimal Information Required In the Annotation of Models (MIRIAM) 1, 23, 54, 73
Portable Document Format (PDF) 77
System for the Analysis of Biochemical Pathways – Reaction Kinetics (SABIO-RK) 2, 4, 5, 13, 14, 20–24, 28, 31, 38–40, 42, 52, 53, 62, 66, 77
Systems Biology Graphical Notation (SBGN) 70
Systems Biology Ontology (SBO) . . . 1, 9, 17, 36, 55, 59, 73
Extended HTML (XHTML) 32, 68
Extended Markup Language (XML) 30

A

ant 9

B

BioModels database 15, 19, 20, 23, 24
BioPAX2SBML 2

C

CellDesigner . . 2, 5, 8–10, 14–18, 20, 28, 29, 36, 69, 71–73, 77, 78

E

Eclipse 35

G

Galaxy 30
Garuda 2, 5, 9, 10, 15, 29, 58, 64, 77–79
Gene 1, 3, 42, 46, 59, 60

J

Java™ . . . 2, 5–7, 9, 10, 35, 41, 62, 63, 71, 74, 76
Java™ Web Start 2, 5, 9, 10, 76
JSBML . . 2, 5, 7–9, 14, 33, 36, 41, 42, 64, 71, 73, 76–79
JUnit 77

K

KEGGtranslator 3, 29

L

Language pack
- Chinese 41, 66, 77
- English 41, 77
- German 41, 77

libSBML . . 2, 5, 7, 10, 13, 36, 41, 42, 64, 68, 71, 72, 76

M

MathML 1

N

Net*Gene*rator 46, 47, 60

O

Operating System . 4–6, 8–11, 13, 15, 30, 34, 40, 77
- Linux 6–8, 10, 12–14, 24, 26, 40
  - `LD_LIBRARY_PATH` 7
  - Ubuntu 6
- Mac OS X . . 6–8, 10, 12–14, 18, 24, 26, 30, 40, 62, 63, 77

DYLD_LIBRARY_PATH 7
Microsoft Windows . 6, 8, 10, 12–14, 24, 26, 40, 41, 79
PATH 7
Windows 7 6
Unix 7, 40

P

Path to models project 3

R

RNA 44, 46, 70

S

SBML . 1–3, 5, 10, 13–15, 17, 20, 24, 26–30, 32, 33, 36–38, 40–42, 45, 46, 54, 55, 58, 71–73, 75–79
SBML2LaTeX . . 1, 3, 4, 15, 31, 54, 55, 64, 68, 72, 77, 79
SBMLsimulator 3, 29

Z

ZIP 10
