# Supplementary material for: SBMLsqueezer 2: context-sensitive creation of kinetic equations in biochemical networks
Source: BMC Syst Biol. 2015 Oct 9;9:68. doi: 10.1186/s12918-015-0212-9 (PMC4600286; doi:10.1186/s12918-015-0212-9)
Supplement: Additional file 2 — Algorithms of SBMLsqueezer. This PDF document explains all main algorithms of SBMLsqueezer in detail using pseudocode and additional descriptions. (141 Kb) [file 12918_2015_212_MOESM2_ESM.pdf]

The algorithms of SBMLsqueezer

Andreas Dräger[1,2,*] Daniel C. Zielinski[1] Roland Keller[2] Matthias Rall[2]
Johannes Eichner[2] Bernhard O. Palsson[1] Andreas Zell[2]

[1]Systems Biology Research Group, University of California, San Diego, La Jolla, CA, USA

[2]Center for Bioinformatics Tuebingen (ZBIT), University of Tuebingen, Tübingen, Germany

This document provides a short overview about the algorithms of the software SBMLsqueezer to group reactions into categories, generate kinetic equations, and to obtain rate laws from the online database SABIO-RK.

Algorithm 1: *De novo* creation of kinetic equations

Input : Model M, Set R of reactions in M for which rate laws are to be created
Parameter: Boolean o decides if existing rate laws should be overwritten, List K_I of KEGG compound identifiers to be skipped, Boolean e decides if enzyme catalysis should be assumed when unspecified, Boolean ρ decides if irreversible reactions should become reversible, Class D default rate law per category
Output : Model M with generated rate laws

$M' \leftarrow$ `submodel` (M, R, o, ρ)
foreach `Reaction` r in M' **do**
  $R_\mathrm{Features} \leftarrow$ `preprocess(`r, K_I`) // Obtain reaction features`
  $K \leftarrow$ `selectRateLaws(`$r, R_\mathrm{Features}, e$`) // Obtain set of applicable rate laws`
  $k \leftarrow$ `prioritize(`K, D`) // Select one rate law from` K
  `createRateLaw(`k, M'`) // Create this rate law and store it in` M'
return $M \leftarrow$ `merge(`M', M`)`

Algorithm 1 shows how kinetic equations are created for an entire model in a single step. The *de novo* creation of rate laws takes as inputs a structural model M, a set R of reactions of interest within this model, and the user preferences o, ρ, K_I, e, and D. The Boolean value o decides whether or not an already existing rate law should be overwritten or kept unchanged. The set of Kyoto Encyclopedia of Genes and Genomes (KEGG) compound identifiers K_I defines those species whose contribution to the rate law is to be neglected. If the Boolean variable e is set to true, reactions without an explicit catalyst are considered enzyme-catalyzed reactions. The value ρ decides if irreversible reactions in R should be set to reversible and rate laws should be

*Corresponding author: andraeger@eng.ucsd.edu

Algorithm 2: Extraction of rate laws from the online database SABIO-RK

Input : Model M, Set R of reactions in M for which rate laws are to be extracted from SABIO-RK
Parameter: Boolean o decides if existing rate laws should be overwritten, Set Q of SABIO-RK query terms
Output : Model M with rate laws from SABIO-RK

foreach `Reaction` r in R **do**
  $M' \leftarrow$ `querySABIORK(`r, Q`)`
  $k \leftarrow$ `extractRateLaw(`M`)`
  $M \leftarrow$ `merge(`k, M, o`)`
return M

accordingly created. In the first step, this algorithm creates a trimmed copy M' of the model, containing only components from M that are required to fully specify the reactions from R. The set D of default rate laws for each category is important for the automatic prioritization of rate laws. The algorithm iterates through all reactions in M'. The preprocessing step creates a set R_{Features} of features for the current reaction r. These features are required to select a set K of applicable rate laws for r. The rate laws in K are subsequently prioritized in order to find the rate law k to be assigned to r. Prioritizing can be done automatically or based on user input. After making this decision, an abstract syntax tree is assembled for the selected rate law and all parameters with their units are created and added to the submodel M'. When all reactions have been processed the submodel M' is merged with the original model M (optionally upon the user's approval).

Algorithm 2 explains how SBMLsqueezer extracts of rate laws from System for the Analysis of Biochemical Pathways – Reaction Kinetics (SABIO-RK). The algorithm again starts with a set R of reactions of interest in model M, but instead of creating a submodel, it iteratively uses given query terms Q to identify a matching model in SABIO-RK for each reaction $r \in R$. If the algorithm can download such a model M', it extracts the rate law for r from this model and merges the result into M upon the user's approval. Finally, both algorithms return the modified input model M. This algorithm can be applied in a batch mode that does not require further user input.

Now let us take a closer look how the subroutines of algorithm 1 on page 1 work. Algorithm 3 on the next page provides details about the preprocessing of reactions, which is required before rate laws can be selected. This algorithm extracts features from reaction r with an optional set of K_{I} KEGG compound identifiers of species whose contribution to rate laws is to be neglected. The sets R_r and P_r contain all reactants and products of r and are combined to the set Y. The values $r_{\text{Stoichiometry}}$ and $p_{\text{Stoichiometry}}$ give the accumulated stoichiometries of the reactants and products. Boolean variables determine several further reaction properties: if all stoichiometry coefficients are integers ($y_{\text{allInteger}}$), if all reactants represent genes (r_{allGenes}) or Ribonucleic Acid (RNA) molecules (r_{allRNA}), if all products represent RNA molecules (p_{allRNA}) or polypeptide chains ($p_{\text{allPolypeptides}}$). Each participant $y \in Y$ has a stoichiometry value and a reference to an actual species x. If x is annotated with a set K_x of KEGG identifiers that contains compound ids from the given ignore list, the element is removed from the reaction. This change does not affect the original model structure M, because this algorithm operates on the trimmed copy M'. Next, the algorithm initializes sets of enzymes E, stimulators A, inhibitors I, and non-enzyme catalysts C. To decide to which of those categories a modifier m belongs, it first looks at this modifier's Systems Biology Ontology (SBO) term and then at the SBO term of the species that corresponds to m. Tables 3 and 4 of the main article give an overview about relevant SBO terms for this decision process.

Algorithm 4 on page 5 shows how SBMLsqueezer selects suitable rate laws. The algorithm obtains as

Algorithm 3: Preprocessing

Input : Reaction r
Parameter: List K_{I} of KEGG compound identifiers to be skipped, can be empty
Output : Set R_{Features} of reaction features

$Y \leftarrow R_r \cup P_r$ `// Build set of reactants and products`
$r_{\mathrm{Stoichiometry}} \leftarrow p_{\mathrm{Stoichiometry}} \leftarrow 0$ `// Lumped stoichiometry of reactants and products`
$y_{\mathrm{allInteger}} \leftarrow$ **true** `// Only integer stoichiometry`
$r_{\mathrm{allGenes}} \leftarrow$ **true** `// All reactants represent genes`
$r_{\mathrm{allRNA}} \leftarrow$ **true** `// All reactants represent RNA molecules`
$p_{\mathrm{allRNA}} \leftarrow$ **true** `// All products represent RNA molecules`
$p_{\mathrm{allPolypeptides}} \leftarrow$ **true** `// All products represent polypeptide chains`
foreach *Reaction participant y in Y* **do**
    $x \leftarrow$ `species(`y`)` `// Lookup the species object for this participant`
    $K_x \leftarrow$ `getKEGGIDs(`x`)` `// Collect all compound identifiers attached to` x.
    if $K_x \cap K_{\mathrm{I}} = \varnothing$ **then**
        `// Neglect the contribution of this participant to the rate law.`
        if $y \in R_r$ **then**
            $R_r \leftarrow R_r \setminus \{y\}$ `// Remove` y `from reactants.`
        else
            $P_r \leftarrow P_r \setminus \{y\}$ `// Remove` y `from products.`
    else
        if $y \in R_r$ **then**
            $r_{\mathrm{Stoichiometry}} \leftarrow r_{\mathrm{Stoichiometry}} +$ `stoichiometry(`y`)`
            $y_{\mathrm{allInteger}} \leftarrow y_{\mathrm{allInteger}} \wedge \left(\{r_{\mathrm{Stoichiometry}}\} \cap \mathbb{Z} \neq \varnothing\right)$
            $r_{\mathrm{allGenes}} \leftarrow r_{\mathrm{allGenes}} \wedge$ `isGene(`x`)`
            $r_{\mathrm{allRNA}} \leftarrow r_{\mathrm{allRNA}} \wedge$ `isRNA(`x`)`
        else
            $p_{\mathrm{Stoichiometry}} \leftarrow p_{\mathrm{Stoichiometry}} +$ `stoichiometry(`y`)`
            $y_{\mathrm{allInteger}} \leftarrow y_{\mathrm{allInteger}} \wedge \left(\{p_{\mathrm{Stoichiometry}}\} \cap \mathbb{Z} \neq \varnothing\right)$
            $p_{\mathrm{allRNA}} \leftarrow p_{\mathrm{allRNA}} \wedge$ `isRNA(`x`)`
            $p_{\mathrm{allPolypeptides}} \leftarrow p_{\mathrm{allPolypeptides}} \wedge$ `isPolypeptide(`x`)`
$E \leftarrow A \leftarrow I \leftarrow C \leftarrow \{\}$
foreach *Modifier m in r* `// Analyze the SBO term of the modifier`
do
    if `isEnzyme(`m`)` **then**
        $E = E \cup \{m\}$
    else if `isCatalyst(`m`)` **then**
        $C = C \cup \{m\}$
    else if `isStimulator(`m`)` **then**
        $A = A \cup \{m\}$
    else if `isInhibitor(`m`)` **then**
        $I = I \cup \{m\}$
    return $R_{\mathrm{Features}} \leftarrow \{r_{\mathrm{Stoichiometry}}, p_{\mathrm{Stoichiometry}}, r_{\mathrm{allGenes}}, r_{\mathrm{allRNA}}, p_{\mathrm{allRNA}}, p_{\mathrm{allPolypeptides}}, E, A, I, C\}$

input a reaction r, its features R_{Features}, and the user's preference e that decides whether or not in the absence of an explicitly declared enzyme the reaction could still be considered enzyme-catalyzed. This algorithm assumes that the reversibility preference ρ has already been incorporated into the model in an earlier step, i.e., as part of the construction of the submodel (see algorithm 1 on page 1). The output is the set K of all applicable rate laws for r. The three major types of reactions are highlighted with background colors: the blue area contains decisions over non-enzyme and non-regulatory reactions, the yellow area comprises all regulatory rate laws, and the green area displays all decisions made about enzyme kinetics. In order to ensure that the set K can never be empty, the algorithm only adds rate laws to K but never removes any rate laws. In all situations, in which rate laws could be removed again, the set B is used as a temporary set of applicable rate laws. All remaining elements of B are finally added to K. The functions $\text{A}(),\ldots,\text{L}()$ return the set of all rate laws in their corresponding category (see Table 1 and Figure 4 of the main article). algorithm 5 on page 6 shows how rate laws for gene-regulatory and enzyme-catalyzed reactions are selected.

Algorithm 5 on page 6 shows how the program selects rate laws for gene regulation and enzyme-catalyzed reactions. These routines are utilized by the function `selectRateLaws` and cannot be used independently. The functions $\text{A}(),\ldots,\text{L}()$ return all rate laws in their respective category (see Figure 4 of the main article).

Algorithm 4: Selection of rate laws based on a reaction and its features

Input : Reaction r

Parameter: Set R_{Features} of reaction features, Boolean e to decide if reactions without explicit catalyst should be considered enzymatic reactions

Output : Set K of applicable rate laws

```
Algorithm selectRateLaws()
    K ← B ← {} // Initialize sets of applicable rate laws
    if R_r ← ∅ ∨ (isReversible(r) ∧ P_r = ∅) then
        if R_r ≠ ∅ // Any reactants present?
        then
            if ¬p_allRNA // RNA produced?
            then
                K ← K ∪ K() // Zeroth order reactant
        else
            if ¬p_allRNA // RNA produced?
            then
                K ← K ∪ (J() ∩ L) // Reversible zeroth order products
        B ← geneRegulation(r, R_Features, e, B)
    else
        if ((E = ∅) ∧ ¬e) ∨ (C ≠ ∅) // Non-enzyme catalyst?
        then
            K ← K ∪ A() // Non-enzyme kinetics
        else
            e ← true
        if (r_Stoichiometry = 1) ∧ (p_Stoichiometry = 1) // Uni-uni stoichiometry?
        then
            B ← enzymeKinetics(r, R_Features, e, B)
        else
            B ← geneRegulation(r, R_Features, e, B)
    if isReversible(r) then
        B ← (B \ H()) ∪ (B ∩ J()) // Remove irreversible kinetics
    else
        B ← (B \ J()) ∪ (B ∩ H()) // Remove reversible kinetics
    return K ← K ∪ B
```

Algorithm 5: Selection of rate laws for gene regulation and enzyme-catalyzed reactions

Input : Reaction r, Set B of potentially applicable rate laws
Parameter: Set R_{Features} of reaction features, Boolean e to decide if reactions without explicit catalyst should be considered enzymatic reactions
Output : Set B of potentially applicable rate laws

Procedure `geneRegulation`(r, R_{Features}, e, B)
- **if** $r_{\text{allGenes}} \vee r_{\text{allRNA}} \vee p_{\text{allRNA}} \vee p_{\text{allPolypeptides}}$ `// Genes, RNA, or polypeptides involved?`
- **then**
  - $B \leftarrow$ `B()` $\setminus$ (`K()` $\cup$ `L()`) `// Gene regulation without zeroth order kinetics`
- **if** $((E = \varnothing) \wedge \neg e) \vee ((C \neq \varnothing) \vee (R_r = \varnothing))$ `// Non-enzyme catalyst or no reactants?`
- **then**
  - $B \leftarrow$ `enzymeKinetics`(r, R_{Features}, e, B)
- **else**
  - $B \leftarrow$ `enzymeKinetics`(r, R_{Features}, **true**, B)
- **return** B

Procedure `enzymeKinetics`(r, R_{Features}, e, B)
- **if** e `// Enzyme involved?`
- **then**
  - $B \leftarrow B \cup$ `F()` `// Arbitrary enzyme kinetics`
  - **if** $(r_{\text{Stoichiometry}} = 1) \wedge (\neg$`isReversible`$(r) \vee (p_{\text{Stoichiometry}} = 1))$ **then**
    - $B \leftarrow B \cup$ `C()` `// Uni-uni enzyme kinetics`
  - **else if** $(r_{\text{Stoichiometry}} = 2) \wedge (\neg$`isReversible`$(r) \vee (p_{\text{Stoichiometry}} = 1))$ **then**
    - $B \leftarrow B \cup$ `D()` `// Bi-uni enzyme kinetics`
  - **else if** $(r_{\text{Stoichiometry}} = 2) \wedge (\neg$`isReversible`$(r) \vee (p_{\text{Stoichiometry}} = 2))$ **then**
    - $B \leftarrow B \cup$ `E()` `// Bi-bi enzyme kinetics`
  - **if** $\neg y_{\text{allInteger}}$ `// Non-integer stoichiometry?`
  - **then**
    - $B \leftarrow B \setminus$ `G()` `// Remove integer kinetics`
  - **if** $(A \neq \varnothing) \vee (I \neq \varnothing)$ `// Any activators or inhibitors?`
  - **then**
    - $B \leftarrow B \cap$ `I()` `// Retain only modulated kinetics`
- **return** B

List of abbreviations and symbols

KEGG Kyoto Encyclopedia of Genes and Genomes

RNA Ribonucleic Acid

SABIO-RK System for the Analysis of Biochemical Pathways – Reaction Kinetics

SBO Systems Biology Ontology

A Set of activators and stimulators

A() . . . L() Functions that return all rate laws in the respective reaction category A. . . L.

B Temporary set of applicable rate laws

C Set of non-enzyme catalysts

D Set of default rate laws for each category

e Boolean variable that is true if all reactions should be assumed enzyme-catalyzed

E Set of enzymes

I Set of inhibitors

k Selected rate law for the current reaction

K Set of potentially applicable rate laws for a reaction

K_{I} Set of KEGG compound identifiers that identify species whose contribution to rate laws should be neglected

K_x Set of KEGG identifiers of a species x

m modifier

M Model

M' Trimmed model copy

o Boolean variable that is true if already existing rate laws should be overwritten

$p_{\text{allPolypeptides}}$ Boolean variable that is true if all products represent polypeptides

$p_{\text{Stoichiometry}}$ Accumulated stoichiometry value of all products

P_r Set of products

Q Set of query terms for SABIO-RK

r Current reaction

r_{allGenes} Boolean variable that is true if all reactants represent genes

r_{allRNA} Boolean variable that is true if all reactants represent RNA molecules

$r_{\text{Stoichiometry}}$ Accumulated stoichiometry value of all reactants

R Set of all reactions in a model

R_{Features} Set of characteristic reaction features

R_r Set of reactants

x Species from vector $\vec{x}$

y Reaction participant

$y_{\text{allInteger}}$ Boolean variable that is true if all participants of a reaction have an integer stoichiometry

Y Set reaction participants

ρ Boolean variable that is true if all reactions should be assumed to be reversible
